# Clinical effectiveness of an online supervised group physical and mental health rehabilitation programme for adults with post-covid-19 condition (REGAIN study): multicentre randomised controlled trial

**DOI:** 10.1136/bmj-2023-076506

**Published:** 2024-02-07

**Authors:** Gordon McGregor, Harbinder Sandhu, Julie Bruce, Bartholomew Sheehan, David McWilliams, Joyce Yeung, Christina Jones, Beatriz Lara, Sharisse Alleyne, Jessica Smith, Ranjit Lall, Chen Ji, Mariam Ratna, Stuart Ennis, Peter Heine, Shilpa Patel, Charles Abraham, James Mason, Henry Nwankwo, Vivien Nichols, Kate Seers, Martin Underwood

**Affiliations:** 1 University Hospitals Coventry and Warwickshire NHS Trust, Coventry, UK; 2 Warwick Clinical Trials Unit, Warwick Medical School, University of Warwick, Coventry, UK; 3 Research Institute for Health and Wellbeing, Coventry University, Coventry, UK; 4 Oxford Psychological Medicine Centre, John Radcliffe Hospital, Oxford, UK; 5 Centre for Care Excellence, University Hospitals Coventry and Warwickshire NHS Trust and Coventry University, Coventry, UK; 6 Department of Anaesthesia and Critical Care, University Hospitals Birmingham NHS Foundation Trust, Birmingham, UK; 7 ICUsteps, Kemp House, London, UK; 8 School of Psychology, Deakin University, Geelong, Australia; 9 Warwick Research in Nursing, Warwick Medical School, University of Warwick, Coventry, UK

## Abstract

**Objective:**

To evaluate whether a structured online supervised group physical and mental health rehabilitation programme can improve health related quality of life compared with usual care in adults with post-covid-19 condition (long covid).

**Design:**

Pragmatic, multicentre, parallel group, superiority randomised controlled trial.

**Setting:**

England and Wales, with home based interventions delivered remotely online from a single trial hub.

**Participants:**

585 adults (26-86 years) discharged from NHS hospitals at least three months previously after covid-19 and with ongoing physical and/or mental health sequelae (post-covid-19 condition), randomised (1:1.03) to receive the Rehabilitation Exercise and psycholoGical support After covid-19 InfectioN (REGAIN) intervention (n=298) or usual care (n=287).

**Interventions:**

Best practice usual care was a single online session of advice and support with a trained practitioner. The REGAIN intervention was delivered online over eight weeks and consisted of weekly home based, live, supervised, group exercise and psychological support sessions.

**Main outcome measures:**

The primary outcome was health related quality of life using the patient reported outcomes measurement information system (PROMIS) preference (PROPr) score at three months. Secondary outcomes, measured at three, six, and 12 months, included PROMIS subscores (depression, fatigue, sleep disturbance, pain interference, physical function, social roles/activities, and cognitive function), severity of post-traumatic stress disorder, general health, and adverse events.

**Results:**

Between January 2021 and July 2022, 39 697 people were invited to take part in the study and 725 were contacted and eligible. 585 participants were randomised. Mean age was 56 (standard deviation (SD) 12) years, 52% were female participants, mean health related quality of life PROMIS-PROPr score was 0.20 (SD 0.17), and mean time from hospital discharge was 323 (SD 144) days. Compared with usual care, the REGAIN intervention led to improvements in health related quality of life (adjusted mean difference in PROPr score 0.03 (95% confidence interval 0.01 to 0.05), P=0.02) at three months, driven predominantly by greater improvements in the PROMIS subscores for depression (1.39 (0.06 to 2.71), P=0.04), fatigue (2.50 (1.19 to 3.81), P<0.001), and pain interference (1.80 (0.50 to 3.11), P=0.01). Effects were sustained at 12 months (0.03 (0.01 to 0.06), P=0.02). Of 21 serious adverse events, only one was possibly related to the REGAIN intervention. In the intervention group, 141 (47%) participants fully adhered to the programme, 117 (39%) partially adhered, and 40 (13%) did not receive the intervention.

**Conclusions:**

In adults with post-covid-19 condition, at least three months after hospital discharge for covid-19, an online, home based, supervised, group physical and mental health rehabilitation programme was clinically effective at improving health related quality of life at three and 12 months compared with usual care.

**Trial registration:**

ISRCTN registry ISRCTN11466448.

## Introduction

Across the World Health Organization European Region during the first two years of the covid-19 pandemic, more than 17 million people may have experienced covid-19 symptoms lasting more than four weeks.^1^ As of March 2023, 1.9 million people in the UK reported covid-19 symptoms persisting beyond 12 weeks, 1.3 million beyond one year, and 762 000 beyond two years.^2^ Common debilitating symptoms of this complex multisystem condition—post-covid-19 condition (long covid)—include fatigue, shortness of breath, cognitive dysfunction, and muscle ache, all of which can profoundly affect quality of life, participation in society, and economic productivity.^3^ Post-covid-19 condition can result in prolonged and unpredictable disability.

Biomedical research has not fully characterised the underlying pathophysiology of post-covid-19 condition; symptom phenotypes are exceptionally diverse.^3^ Consequently, existing medical management and drug treatments are limited in effectiveness and generalisability. The biopsychosocial model of care may contribute to improved outcomes for people with post-covid-19 condition. Multicomponent physical and mental health rehabilitation can improve breathlessness, fatigue, and quality of life in other long term conditions.^4^ ^5^ ^6^ To date, only small quasi-experimental studies have investigated exercise based rehabilitation interventions for people with post-covid-19 condition, and no high quality definitive evidence exists as to the potential benefits or harms of physical and mental health rehabilitation interventions.

We assessed the clinical effectiveness of an eight week live online group rehabilitation programme—the Rehabilitation Exercise and psycholoGical support After COVID-19 InfectioN (REGAIN) intervention—versus a single online session of advice and support for people with post-covid-19 condition.

## Methods

### Trial design and setting

The REGAIN trial was a pragmatic, multicentre, parallel group, superiority randomised controlled trial with embedded process evaluation, recruiting throughout England and Wales. Each participant identification centre was granted NHS Trust site specific approval. The NHS Digital “Digi-Trials” service was approved to identify and invite potential participants in accordance with Regulation 3(4) of the Health Service (Control of Patient Information, COPI) Regulations 2002, requiring NHS Digital to share confidential patient information with organisations entitled to process this under COPI for the purposes of covid-19 research.^36^ The trial protocol and details of the intervention’s development have been published previously^7^ ^8^ and are reported in accordance with the template for intervention description and replication (TIDieR)^9^ (see supplementary material).

### Participants and procedures

Participants were adults (26-86 years) who had been discharged from hospital three or more months previously after hospital admission with covid-19 and who had ongoing substantial (as defined by participants) covid-19 related physical and/or mental health sequelae. In the absence of agreed diagnostic criteria or clinical coding for post-covid-19 condition, participants were asked to self-report any substantial lasting effects that they attributed to their hospital admission with covid-19. This was confirmed during an eligibility telephone call with the clinical trial team before study enrolment. Exclusion criteria were contraindication to exercise training; severe mental health problems preventing engagement with study procedures; previous randomisation in the present study; already engaged, or planning to engage, in an alternative NHS rehabilitation programme in the next three months; or a household member had been randomised into the REGAIN trial previously.

### Randomisation and masking

After completion of the online consent and baseline questionnaires (to ensure allocation concealment), participants were randomly allocated (1:1.03) to the REGAIN intervention or to usual care by a centralised computer generated randomisation sequence using a bespoke web based system, administered independently by Warwick Clinical Trials Unit. We used a minimisation algorithm, stratified by age (<65 years *v* ≥65 years), level of hospital care (intensive care unit (ICU) or high dependency unit (HDU) *v* ward), and case level mental health symptomatology (impact of event scale-6 (IES-6) post-traumatic stress disorder score ≥11/24, or hospital anxiety and depression scale (HADS) anxiety subscore ≥11/21, or HADS depression subscore ≥11/21; compared with IES-6 post-traumatic stress disorder score <11/24, or HADS anxiety subscore <11/21, or HADS depression subscore <11/21) (see supplementary material). Participants and practitioners delivering REGAIN could not be masked to group allocation. Follow-up outcome assessments were completed by participants online, or, in a small number of cases, over the telephone by a member of the trial team, blind to treatment allocation.

### Procedures

Potential participants were contacted by post, either locally through secondary care NHS Trusts or, for England and Wales, through an NHS Digital “Digi-Trials” mailout. Self-referral to the trial was also possible. Those with persistent physical or mental health sequelae, or both (estimated at 10% of people discharged from hospital after covid-19)^10^ were invited to register their interest by completing a brief online questionnaire for suitability. On confirmation of suitability, the clinical research team contacted participants by telephone to complete further eligibility checks. For those who self-referred, eligibility was confirmed through their general practitioner. Participants then completed an online consent form and baseline outcomes questionnaire before randomisation. We informed general practitioners in writing of participants whose baseline (or follow-up) scores met any of our predefined criteria for case level mental health (see supplementary material). These people continued in the trial. Trial and intervention materials were translated into the five most spoken non-English languages in the UK (Bengali, Gujarati, Urdu, Punjabi, and Mandarin) and a non-English speaking pathway developed to allow access to the trial.

Clinical exercise physiologists or physiotherapists trained in the REGAIN intervention and supported by health psychologists delivered interventions exclusively online from a central trial hub. The hub was based at Atrium Health, a non-profit rehabilitation centre, subcontracted to University Hospitals of Coventry and Warwickshire NHS Trust. Intervention staff included NHS and Atrium Health employees, with some delivering both intervention and usual care treatments. Interventions were informed by a rapid review of existing literature relating to rehabilitation programmes for people affected by chronic obstructive pulmonary disease, chronic fatigue syndrome, and the 2003 severe acute respiratory distress syndrome pandemic. Details on co-development of the intervention are provided elsewhere.^8^

### Interventions

Participants in the usual care group received best practice usual care, consisting of a 30 minute, online, one-to-one consultation with a trained practitioner. A trial booklet was provided that incorporated components of the NHS England “your covid recovery” programme,^11^ information and advice that is freely available online. During the one-to-one consultation, practitioners used the NHS England your covid recovery programme as a template to discuss hospital admission with covid-19, resulting physical and mental health sequalae, and other relevant medical history with participants. The consultation covered generic advice as to how participants might facilitate recovery and undertake self-directed physical activity. A structured physical activity plan was not provided, and no specific psychological techniques were used.

The REGAIN intervention comprised an eight week, online, home based, supervised, group rehabilitation programme (see supplementary figure S1), supported by a workbook for participants (https://wrap.warwick.ac.uk). Participants received a one hour, online, one-to-one consultation with a REGAIN practitioner, which provided an opportunity to discuss hospital admission with covid-19 and sequelae, medical history, and practical ways in which physical and mental health recovery could be supported. Participants subsequently enrolled on weekly practitioner led live (ie, synchronous) online group exercise sessions and six live online group psychological support sessions (one hour each) delivered through Zoom using the Beam platform (https://www.beamfeelgood.com). Individualised, equipment-free exercise sessions performed at home in online groups under the supervision of a REGAIN practitioner aimed to improve cardiovascular fitness, strength, balance, and fatigue while restoring confidence in completing activities of daily living. Semistructured facilitated psychological support sessions were designed to enhance psychological capability and increase knowledge and understanding of covid-19 and its impact on daily living, while giving participants the opportunity to share their own experiences with the group (≤12 participants). Topics of discussion, supported by short introductory videos, included motivation, fear avoidance, activity pacing, managing emotions and set-backs, sleep and fatigue, and stress and anxiety management. Finally, a library of prerecorded, on-demand physical activity videos was made available for participants to access independently online as required. Sessions ranged in duration and intensity from simple breathing exercises, Pilates, and yoga to light seated activity and upright moderate to high intensity exercise.

### Outcomes

Outcomes were measured at three, six, and 12 months. The primary outcome was health related quality of life using the patient reported outcomes measurement information system (PROMIS) 29+2 Profile v2.1 at three months post-randomisation.^12^ This measure is one of a portfolio of outcomes from the US National Institutes of Health. The system is reliable, generic, and validated for online use, generating a single overall preference based score (absolute score rather than effect size)—the PROMIS preference (PROPr) score (range −0.022 to 1.0, where 0 indicates a health state equivalent to death and 1.0 indicates perfect health).^12^ The overall score is generated from seven subscores for depression, fatigue, sleep disturbance, pain interference, physical function, social roles or activities, and cognitive function. As with other preference based measures such as the EuroQol 5 dimension 5 level (EQ-5D-5L) instrument, a difference of 0.03 to 0.05 is considered to be clinically important.^13^

For analysis purposes, we rescaled raw data from the seven PROMIS subscores to standardised T scores with a mean of 50 and a standard deviation (SD) of 10. Therefore, a person with a T score of 40 is 1 SD below the mean. Higher T scores represent more of the concept being measured. For negatively worded concepts such as pain interference, a T score of 60 is 1 SD worse than the mean, whereas for positively worded concepts such as physical function, a T score of 60 is 1 SD better than the mean. For anxiety, depression, fatigue, pain interference, and sleep disturbance, higher scores indicate more severe symptoms. For physical function and social participation, higher scores indicate better health outcomes. A change in T score of between 2.0 and 6.0 is considered clinically important in the PROMIS subscores and subscales.^14^ A further two PROMIS subscales independently measured anxiety and pain intensity. All subscores and subscales were rated over the preceding seven days, apart from physical function and social roles or activities, which do not have a specified timeframe.

Our secondary outcomes were dyspnoea (PROMIS dyspnoea severity short form), cognitive function (PROMIS Neuro-QoL short-form v2.0-cognitive function), quality of life (EQ-5D-5L),^15^ physical activity (international physical activity questionnaire short-form, IPAQ),^16^ severity of post-traumatic stress disorder (impact of events scale-revised, IES-R),^17^ anxiety and depression (hospital anxiety and depression scale, HADS),^18^ general health (self-report of current overall health compared with baseline), and mortality.

Adverse events and serious adverse events were recorded in both trial arms, in line with the principles of good clinical practice.^19^ Additionally, participants in the intervention group were asked to report any events before each exercise session through a confidential, secure online poll. The trial team routinely reviewed responses and contacted participants for further information as required. As the presentation of post-covid-19 condition and chronic fatigue syndrome/myalgic encephalomyelitis overlaps,^3^ we prospectively monitored for post-exertional symptom exacerbation^20^ in the intervention arm during each contact of participants with the intervention team.

Full adherence to the REGAIN intervention was defined as attendance at the initial one-to-one session along with completion of four or more of six support sessions and five or more of eight exercise sessions. Partial adherence was defined as attendance at the initial one-to-one session and completion of fewer than four of six support sessions and fewer than five of eight exercise sessions. To assess fidelity to the intervention, we reviewed a randomly selected 5% subsample of video recorded one-to-one sessions and group exercise and support sessions against predetermined checklist criteria. Results of this and the rest of our process evaluation will be reported elsewhere.

### Sample size

The sample size was calculated based on identifying a small to moderate standardised mean difference of 0.3. No data exist on which to base a sample size estimation, nor normative data for the PROPr health related quality of life score in a covid-19 population. Also, there is no indication of what a worthwhile benefit might be from the intervention. We inflated the size of the intervention group to compensate for any clustering effect owing to the delivery of the group intervention. We assumed, based on our experience with other rehabilitation interventions, that groups would comprise a maximum of eight participants. Assuming an intracluster coefficient of 0.01, 90% power, and type I error rate of 5%, with a 10% loss to follow-up, we determined that 535 participants would be required. This equated to 272 participants in the intervention arm (up to 34 intervention groups) and 263 participants in the usual care arm (allocation intervention to usual care ratio of 1.03:1).^21^ To compensate for the slightly higher than anticipated loss to follow-up in the observed data, we recruited a total of 585 participants.

### Statistical analysis

Statistical analysis followed a predefined plan.^22^ Our primary analysis was done on an intention-to-treat basis, which included all participants randomly assigned to a treatment group. For the primary outcome (PROPr score) we performed a partially nested heteroscedastic model^23^ to compare health related quality of life at three months between the REGAIN intervention group and usual care group, producing unadjusted and adjusted estimates. Adjustments were made using age, level of hospital care, level of mental health disorder, baseline PROPr score, and therapist effect as a random effect. Deaths were included with a score of zero. The only ordinal categorical outcome was the overall health score. This outcome was fitted using linear regression models (for unadjusted and adjusted variables). We checked normality assumptions and used the Mann-Whitney test to test the treatment effect (unadjusted). To accommodate for non-adherence, we did a complier average causal effect analysis based on a single equation instrumental variable regression model. The complier average causal effect estimates the treatment effect in people randomly assigned to the intervention who actually received it by comparing participants who fully or partially adhered in the intervention group with participants in the control group who would have been classed as adherent had they been allocated to the intervention group.

We performed unadjusted analyses of subgroups defined according to age (<65 years *v* ≥65 years), level of hospital care (critical care *v* standard ward), HADS depression and HADS anxiety score (<11 *v* ≥11), severity of post-traumatic stress disorder (impact of events scale-6 (six item subscale of the impact of events scale-6-revised) score (<11 *v* ≥11), ethnicity (white *v* non-white), wave of pandemic (first, second, third, or fourth), and method of recruitment (NHS Digital *v* NHS Trusts or self-referral). In a sensitivity analysis, we used the multiple imputation by chained equations procedure,^24^ imputing the primary outcome. To aid interpretation, we report the number needed to treat based on the responses on the global health transition question. These responses are presented as a number needed to treat to be “much better” and at least “somewhat better.” All analyses were conducted using Stata version 17 and R version 4.

### Study monitoring

The data monitoring and trial steering committees reviewed the progress of the trial and safety periodically (see supplementary material).

### Patient and public involvement

The concept for the trial and grant funding application was driven by our patient partner working group early in the covid-19 pandemic. Patient representatives were involved as co-applicants in the grant funding application. On receipt of the award, our patient and stakeholder working group were integral to the rapid design, co-creation, and pilot testing of the REGAIN intervention and trial processes.^8^ Subsequently, patient partners participated as members of the trial management group and trial steering committee.

## Results

**Fig 1:**
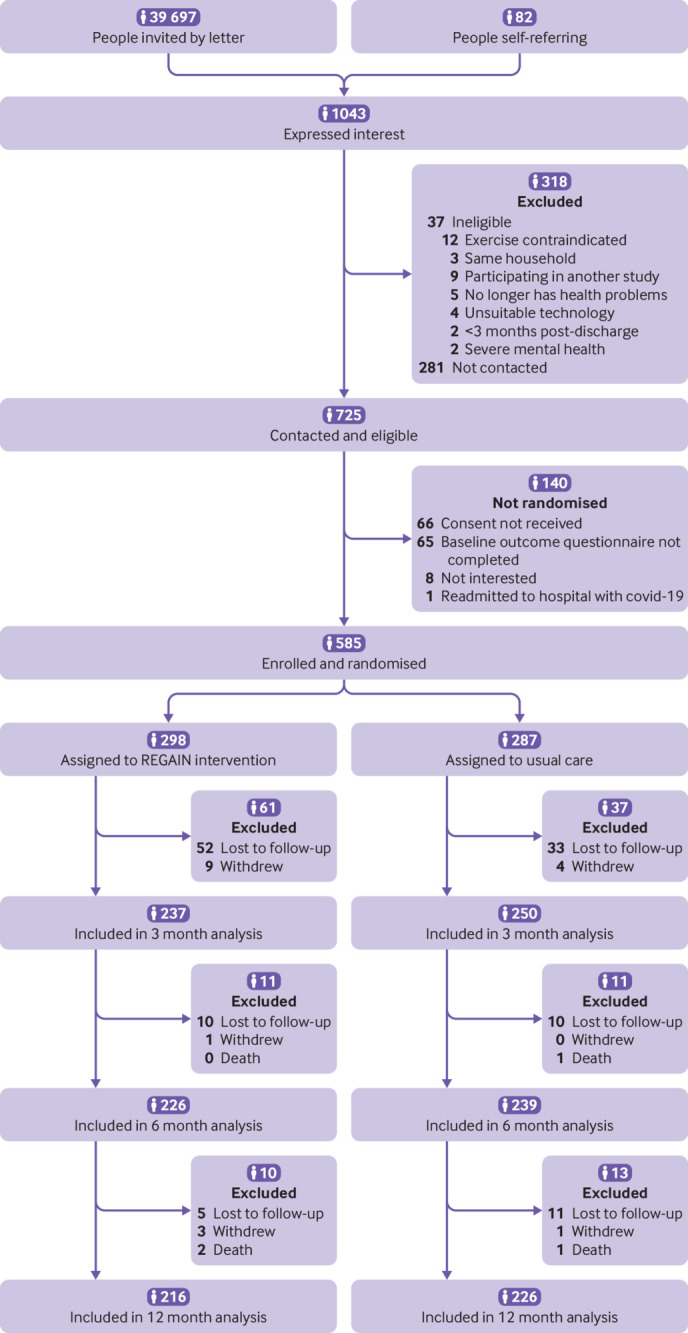
Flow of participants through study

Between January 2021 and July 2022, 39 697 people were invited to take part in the study (about 10% (n=4000) were anticipated to meet our definition of post-covid-19 condition)^10^ and 82 self-referred (fig 1). Of 1043 people expressing an interest to participate in the study, 725 (70%) people were contacted and eligible. Overall, 140/725 (19%) people were not randomised for the following reasons: not interested (n=8), consent not received (n=66), baseline outcome questionnaire not completed (n=65), and readmission to hospital with covid-19 (n=1). We randomised 585 people: 298 (51%) to the REGAIN intervention and 287 (49%) to usual care.

Mean age of the study sample was 56 (SD 12) years, more than half were female participants (305/585; 52%), and most were of white ethnicity (517/585, 88%) (table 1). Overall, 508/585 (88%) participants had overweight or obesity, and one third (201/585; 34%) had been admitted to ICU or HDU during their hospital admission with covid-19. Mean time from hospital discharge to randomisation was 323 (SD 144) days (10.6 months). Baseline health related quality of life was low (mean PROPr score 0.20 (SD 0.17))^12^ with a high prevalence of case level mental health disorder (n=251/585; 43%) (table 1; also see supplementary material). Physical activity levels were low (<600 MET (metabolic equivalent of task) mins/week)^16^ for less than half of the participants (235/585; 40%) (table 1). The most common pre-existing medical conditions related to chest or breathing (444/585; 76%) and musculoskeletal conditions (275/585; 47%), and more than one third of participants were unable to work owing to post-covid-19 condition (222/585; 38%) (table 1).

**Table 1:** Baseline characteristics of participants assigned to receive an eight week online group rehabilitation programme* or usual care. Values are number (percentage) unless stated otherwise

Characteristics	Intervention (n=298)	Usual care (n=287)	Total (n=585)
Mean (SD) age (years)	56.1 (12.1)	56.2 (12.3)	56.1 (12.2)
Female sex	162 (54)	143 (50)	305 (52)
Non-white ethnicity	33 (11)	35 (12)	68 (11)
Mean (SD) body mass index	33.0 (7.7)	32.8 (8.0)	32.9 (7.8)
Smoking status:			
Current smoker	8 (3)	4 (1)	12 (2)
Former smoker	118 (40)	121 (42)	239 (41)
Never smoker	172 (58)	162 (56)	334 (57)
Employment status:			
Post-school training	230 (77)	233 (81)	463 (79)
Full time work	160 (54)	162 (57)	322 (55)
Part time work	45 (15)	37 (13)	82 (14)
Full/part time education	3 (1)	0 (0)	3 (0)
Unemployed	10 (3)	4 (1)	14 (2)
Retired	49 (16)	62 (22)	111 (19)
Unable to work, health	27 (9)	20 (7)	47 (8)
Other	4 (1)	2 (1)	6 (1)
Unable to work, covid-19	125 (42)	97 (34)	222 (38)
Comorbidities:			
Heart or circulation	77 (26)	99 (35)	176 (30)
Chest or breathing	226 (76)	218 (76)	444 (76)
Kidney or bladder	50 (17)	53 (19)	103 (18)
Stomach, bowel, or abdomen	93 (31)	83 (29)	176 (30)
Endocrine	92 (31)	83 (29)	175 (30)
Musculoskeletal	143 (48)	132 (46)	275 (47)
Brain or nervous system	67 (23)	67 (23)	134 (23)
Blood or clotting	48 (16)	62 (22)	110 (19)
Other health problem	117 (39)	123 (43)	240 (41)
Admission to ICU/HDU	102 (34)	99 (35)	201 (34)
Mean (SD) time from discharge (days)	331 (151)	314 (137)	323 (144)
Mean (SD) PROPr score	0.20 (0.17)	0.20 (0.17)	0.20 (0.17)
EQ-5D-5L:			
Mean (SD) index score	0.55 (0.27)	0.55 (0.25)	0.55 (0.26)
Mean (SD) VAS score	55.6 (19.7)	53.2 (19.9)	54.4 (19.8)
Mean (SD) PTSD IES-R total score	30.3 (19.7)	31.0 (19.7)	30.6 (19.7)
PTSD IES-6:			
Mean (SD) total score†	9.06 (6.0)	9.25 (6.0)	9.15 (6.0)
Score ≥11†	114 (38)	116 (40)	230 (39)
HADS anxiety:			
Mean (SD) score	9.0 (5.3)	9.4 (4.9)	9.2 (5.1)
Score ≥11†	122 (41)	120 (42)	242 (41)
HADS depression:			
Mean (SD) score	8.8 (4.7)	9.0 (4.5)	8.9 (4.6)
Score ≥11†	101 (34)	109 (38)	210 (36)
Mean (SD) PROMIS:			
Dyspnoea	55.1 (8.7)	55.4 (8.6)	55.2 (8.7)
Cognitive	39.4 (9.2)	39.0 (9.2)	39.2 (9.2)
IPAQ-SF (MET mins/week):			
<600 (low)	130 (44)	105 (37)	235 (40)
≥600-3000 (moderate)	89 (30)	98 (34)	187 (32)
≥3000 (high)	78 (26)	84 (29)	162 (28)

SD=standard deviation; ICU=intensive care unit; HDU=high dependency unit; PROPr=patient reported outcomes measurement information system (PROMIS) preference score; EQ-5D-5L=EuroQol 5 dimension 5 level; VAS=visual analogue scale; PTSD=post-traumatic stress disorder; IES-R=index of event scale-revised; IES-6=index of event scale-6; HADS=hospital anxiety and depression scale; IPAQ-SF=international physical activity questionnaire-short form; MET=metabolic equivalent of task (1 MET is equivalent to resting energy expenditure (ie, 3.5 mL/kg/min)).

Percentages may add up to >100% as participants could be included in multiple categories.

* Rehabilitation Exercise and psycholoGical support After COVID-19 InfectioN (REGAIN) intervention.

† Threshold for case level mental health disorder.

**Fig 2:**
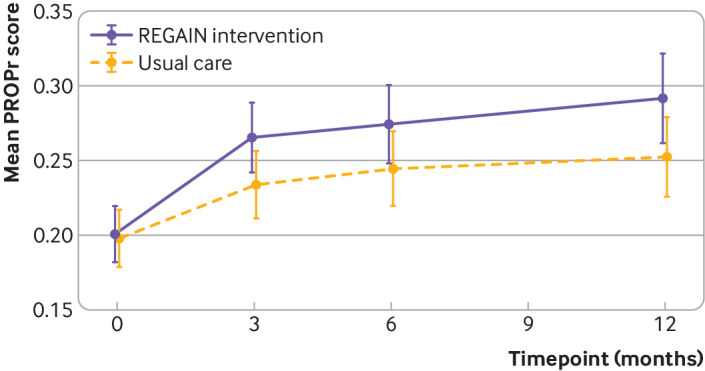
Mean patient reported outcomes measurement information system preference (PROPr) score at three, six, and 12 months by treatment arm. Higher scores indicate better quality of life. Whiskers represent 95% confidence intervals

**Fig 3:**
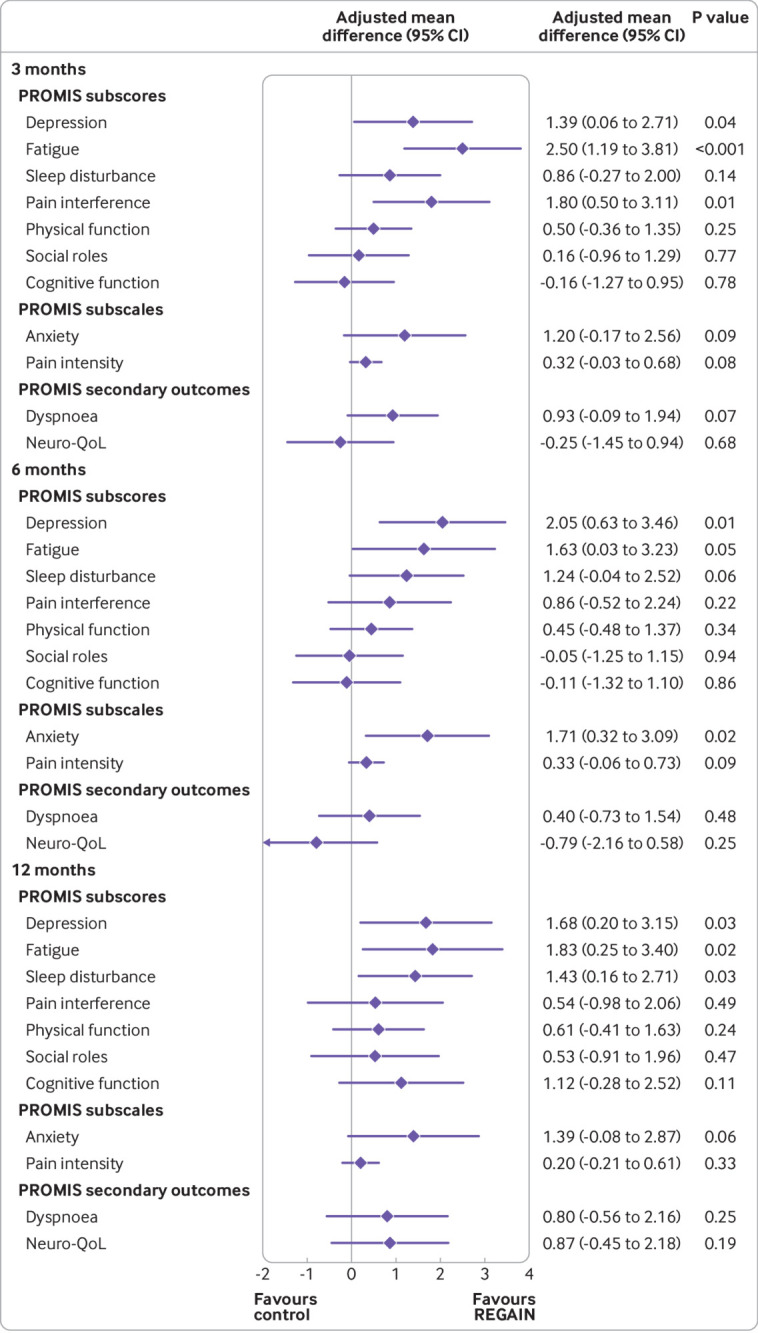
Adjusted mean difference (95% confidence interval) between groups in PROMIS subscores, subscales, and secondary outcomes at three, six, and 12 months. PROMIS= patient reported outcomes measurement information system. Neuro-Qol=PROMIS Neuro-QoL short-form v2.0-cognitive function

Primary outcome data were collected from 237/298 (80%) in the REGAIN intervention group and 248/287 (86%) participants in the usual care group (table 2). At three months, health related quality of life improved more for participants in the intervention group (mean PROPr score 0.27 (SD 0.18); n=237) than the usual care group (0.23 (SD 0.18); n=248) (also see supplementary tables S9 and S12). We observed a statistically significant difference in health related quality of life between groups at three months (adjusted mean difference in PROPr score 0.03 (95% confidence interval 0.01 to 0.05), P=0.02) (table 2 and fig 2). This was driven predominantly by between group differences in three PROMIS subscores: depression (1.39 (0.06 to 2.71), P=0.04), fatigue (2.50 (95% confidence interval 1.19 to 3.81), P<0.001), and pain interference (1.80 (0.50 to 3.11), P=0.01), favouring the REGAIN intervention (fig 3). The effect of the intervention was also evident at 12 months (adjusted mean difference in PROPr score 0.03 (95% confidence interval 0.01 to 0.06), P=0.02), but not at six months (0.02 (−0.003 to 0.05), P=0.08) (table 2 and fig 2; also see supplementary tables S9-S11). At 12 months, improvements in the subscores for depression (1.68 (0.20 to 3.15), P=0.03) and fatigue (1.83 (95% confidence interval 0.25, to 3.40), P=0.02) were sustained (see supplementary table S11).

**Table 2:** Estimates of treatment difference for primary outcome (patient reported outcomes measurement information system preference (PROPr) score at three, six, and 12 months

PROPr	Intervention (n=298)	Usual care (n=287)	Total (n=585)	Estimate (95% CI)*; P value		
				ITT	CACE (full)†	CACE (full+partial)†
**3 months**						
No of participants	237	248	485	0.03 (0.01 to 0.05); 0.02	0.05 (0.01 to 0.09); 0.01	0.03 (0.01 to 0.05); 0.01
Mean (SD)	0.27 (0.18)	0.23 (0.18)	0.25 (0.18)			
Median (IQR)	0.24 (0.13-0.37)	0.21 (0.10-0.34)	0.22 (0.11-0.36)			
**6 months**						
No of participants	225	237	462‡	0.02 (−0.003 to 0.05); 0.08	0.04 (−0.004 to 0.08); 0.08	0.02 (−0.003 to 0.05); 0.08
Mean (SD)	0.27 (0.20)	0.24 (0.20)	0.26 (0.20)			
Median (IQR)	0.24 (0.12-0.39)	0.21 (0.11-0.34)	0.225 (0.11-0.37)			
**12 months**						
No of participants	217	227	444§	0.03 (0.01 to 0.06); 0.02	0.06 (0.01 to 0.10); 0.02	0.04 (0.01 to 0.07); 0.02
Mean (SD)	0.29 (0.22)	0.25 (0.20)	0.27 (0.22)			
Median (IQR)	0.25 (0.12-0.43)	0.21 (0.09-0.37)	0.23 (0.10-0.40)			

ITT=intention to treat; CACE=complier average causal effect; SD=standard deviation; IQR=interquartile range.

* Based on a partially nested heteroscedastic model adjusted for baseline overall health and stratification variables (age, level of hospital care, and level of mental health disorder); the therapist effect was included as a random effect to account for partial clustering.

† Based on a single equation instrumental variable regression model with outcome adjusted for baseline overall health and stratification variables (age, level of hospital care, and level of mental health disorder).

‡ Includes one participant who died at this time point.

§ Includes four participants who died at this time point.

Figure 3 and table 3 show the secondary outcomes at three months, and supplementary table S3 shows descriptions of the scale ranges. Supplementary tables S14 and S15 show results at six and 12 months. At three months, all PROMIS subscores and subscales, apart from cognitive function (PROMIS cognitive function subscore; PROMIS Neuro-QoL short-form) were positively influenced to a greater extent by the REGAIN intervention compared with usual care (fig 3). By 12 months, all PROMIS subscores and subscales were improved more in the intervention group. Greater improvements were also noted in favour of the REGAIN intervention for the EQ-5D-5L visual analogue scale at three months (3.37 (95% confidence interval 0.23 to 6.51), P=0.04) and 12 months (3.77 (0.32 to 7.22), P=0.04); and the post-traumatic stress disorder impact of events scale-revised total score at three months (2.61 (0.08 to 5.14), P=0.04) and 12 months. (4.37 (1.66 to 7.07), P=0.002).

**Table 3:** Secondary outcomes at three months’ follow-up by treatment arm. Values are number (percentage) unless stated otherwise

Outcomes	Intervention (n=298)	Usual care (n=287)	Total (n=585)	Adjusted estimate (95% CI)*; P value
**EQ-5D-5L index**				
No of participants	237	245	482	0.02 (−0.01 to 0.05); 0.26
Mean (SD)	0.6 (0.3)	0.6 (0.25)	0.6 (0.3)	
Median (IQR)	0.8 (0.5-0.8)	0.65 (0.5-0.7)	0.65 (0.5-0.7)	
**EQ-5D-5L VAS**				
No of participants	236	245	481	3.37 (0.23 to 6.51); 0.04
Mean (SD)	62.3 (19.1)	57.6 (21.6)	59.9 (20.5)	
Median (IQR)	61.5 (50.0-77.5)	60.0 (41.0-75.0)	60.0 (46.0-76.0)	
**PTSD IES-R total score**				
No of participants	192	188	380	2.61 (0.08 to 5.14); 0.04
Mean (SD)	24.6 (18.0)	28.1 (20.1)	26.4 (19.1)	
Median (IQR)	21.0 (11.0-35.5)	26.0 (11.0-42.5)	23.5 (11.0-39.5)	
**PTSD IES-6 total score**				
No of participants	237	248	485	
Score ≥11	79 (33)	114 (46)	193 (40.0)	
**HADS anxiety**				
No of participants	212	214	426	0.29 (−0.37 to 0.94); 0.38
Mean (SD)	8.0 (4.8)	8.6 (4.8)	8.3 (4.8)	
Median (IQR)	8.0 (4.0-11.0)	8.0 (5.0-12.0)	8.0 (5.0-12.0)	
Score ≥11	77 (34)	85 (38)	162 (36)	
**HADS depression**				
No of participants	206	216	422	0.46 (−0.14 to 1.05); 0.13
Mean (SD)	7.7 (4.5)	8.4 (4.8)	8.1 (4.6)	
Median (IQR)	7.0 (4.0-10.0)	8.0 (5.0-11.0)	8.0 (4.0-11.0)	
Score ≥11	65 (29)	78 (35)	143 (32)	
**IPAQ-SF (MET min/week)**				
No of participants	221	222	443	1.66‡ (1.14 to 2.41); 0.01
<600 (low)	59 (27)	76 (34)	135 (31)	
≥600-3000 (moderate)	77 (35)	66 (30)	143 (32)	
≥3000 (high)	85 (38)	80 (36)	165 (37)	
**Health *v* 3 months ago**				
No of participants	216	220	436	0.30 (0.13 to 0.46); 0.001
Much better now	39 (17)	20 (8)	59 (12)	
Somewhat better now	81 (34)	60 (24)	141 (29)	
About the same	72 (30)	108 (44)	180 (37)	
Somewhat worse	19 (8)	27 (11)	46 (10)	
Much worse	5 (2)	5 (2)	10 (2)	

EQ-5D-5L=EuroQol 5 dimension 5 level; VAS=visual analogue scale; PTSD=post-traumatic stress disorder; IES-R=index of event score-revised; HADS=hospital anxiety and depression score; PROMIS=patient reported outcomes measurement information system; IPAQ-SF=international physical activity questionnaire-short form.

* Based on a partially nested heteroscedastic model adjusted for baseline overall health and stratification variables (age, level of hospital, and level of mental health disorder). The therapist effect was included as a random effect to account for partial clustering.

‡ Odds ratio; mixed effect ordered logistic regression model. A score ≥11 for PTSD-IES-6, HADS anxiety, or HADS depression was the threshold for case level mental health disorder—these categorical data have not been statistically analysed.

In addition to continuous outcome data, we present categorical data for IES-6 post-traumatic stress disorder, HADS anxiety, and HADS depression as these were used to identify case level mental health disorder, defined as a score ≥11 for any of the three measures (table 3). We have not tested the statistical significance of these categorical data, as this was not specified in our statistical analysis plan. However, given a statistical difference between groups in the continuous post-traumatic stress disorder symptom severity data (IESR-R) at three months, it is perhaps noteworthy that 33% of participants in the REGAIN intervention group compared with 46% in the usual care group, exceeded the threshold for case level post-traumatic stress disorder (IES-6).

Participants in the REGAIN intervention group had higher odds (odds ratio 1.66, 95% confidence interval 1.14 to 2.41; P=0.01) of being more physically active compared with participants in the usual care group (table 3). At three months, compared with usual care, 7% more people in the REGAIN intervention group were achieving the UK Chief Medical Officers’ physical activity guidelines^25^ of >150 minutes of moderate intensity activity per week (>600 MET min/week). No effect was seen at six or 12 months. For the category of “overall health compared to three months ago,” a higher proportion of participants in the REGAIN intervention group reported feeling “much better now” (39/237; 17% *v* 20/250; 8%) and “much better now” or “somewhat better now” combined (120/237; 51% *v* 80/250; 32%). This equates to a number needed to treat of 11.9 and 5.4, respectively. The remainder of the secondary outcomes showed no statistically significant differences between groups.

In the usual care and REGAIN intervention groups, we recorded several adverse events (n=16 (6%); n=28 (9%), respectively) and serious adverse events (n=7 (2%); 14 (5%), respectively) (see supplementary material). Of the 21 serious adverse events, 19 concerned admission to hospital or prolongation of admission, and two involved persistent or major disability or incapacity. Only one serious adverse event (syncope with vomiting 24 hours after a live exercise session) was possibly related to the REGAIN intervention. Two adverse events were definitely related (unilateral knee pain during a live exercise session; severe anxiety before a live exercise session) and two were probably related (anxiety before a live exercise session; headache during a live exercise session) to the REGAIN intervention. No instances of post-exertional symptom exacerbation were identified during weekly monitoring.

Adherence to the REGAIN intervention was good. Of 298 participants, 141 (47%) fully adhered, 117 (39%) partially adhered, and 40 (13%) did not receive the intervention (see supplementary material). Median attendance was 5.0 (interquartile range 2.0-7.0) at live group exercise sessions and 5.0 (2.0-6.0) at live group support sessions. At three months, the complier average causal effect analysis for the primary outcome (PROPr) in the groups that fully and fully plus partially adhered was 0.05 (95% confidence interval 0.01 to 0.09), P=0.01) and 0.03 (0.01 to 0.05), P=0.01), respectively (table 2). The supplementary material presents a detailed breakdown of adherence.

No difference in effect was identified in prespecified subgroup analyses relating to age, level of hospital care, HADS depression or anxiety scores, severity of post-traumatic stress disorder, ethnicity, wave of pandemic, or method of recruitment (see supplementary material). In our sensitivity analyses, we observed a statistically significant difference in health related quality of life between groups at three months after adjusting for additional covariates such as sex, body mass index, and ethnicity (adjusted mean difference 0.03 (95% confidence interval 0.01 to 0.05), P=0.01) (see supplementary material), and after multiple imputation for data missingness (0.03 (0.01 to 0.05), P=0.02) (see supplementary material).

## Discussion

For adults who experienced post-covid-19 condition after hospital admission with covid-19, a structured programme of physical and mental health rehabilitation (REGAIN), delivered in groups online was clinically effective compared with usual care for improving health related quality of life (PROPr) in our primary analysis at three months post-randomisation. Predominantly, this effect was driven by significantly greater improvements in the PROMIS fatigue, depression, and pain interference subscores with the REGAIN intervention. The intervention was acceptable and safe, as indicated by a single serious adverse event considered to be possibly related to the intervention. Furthermore, the effects of the intervention were also evident at 12 months.

We observed improvements in overall quality of life and in other indices of wellbeing with both the REGAIN intervention and usual care. The relative contributions of the brief intervention, the natural recovery from postviral illness, and regression to the mean in the control group is unclear. Most likely natural recovery played an important part in the improvements witnessed in both groups, as identified in recent observational data.^26^ The REGAIN intervention did, however, show an additional benefit above that which could be attributed to natural recovery and the best practice usual care intervention. Research completed since we started this study suggests a minimally important difference of 0.04 on the PROPr score between groups.^13^ Our observed differences of 0.03 (95% confidence interval 0.01 to 0.05) at three months and 0.03 (0.01 to 0.06) at 12 months are smaller than this suggestion. However, the complier average causal effect analysis showed a larger effect of 0.05 (0.01 to 0.09) at three months and 0.06 (0.01 to 0.10) at 12 months, suggesting that the true effect, in those fully complying with the intervention, might exceed this threshold. Our post hoc analysis showing numbers needed to treat of 11.9 for “much better now” and 5.4 for “much better now” or “somewhat better now” combined at three months will help to interpret the clinical importance of our findings.

The PROPr score for health related quality of life is calculated from seven PROMIS subscores, and in addition we measured four separate PROMIS subscales. At three months, nine of 11 scores were influenced more favourably by the REGAIN intervention compared with usual care. Only two constructs (PROMIS cognitive function subscore; PROMIS Neuro-QoL short-form), both assessing cognitive function, were not improved more by the intervention compared with usual care. By 12 months, all 11 scores were influenced more favourably by the REGAIN intervention. The clinically important improvements we witnessed in the PROMIS fatigue, depression, and pain interference subscores may be important. Fatigue is one of the most prevalent and debilitating symptoms associated with post-covid-19 condition. As with other postviral and autoimmune conditions, post-covid-19 condition is pervasive and enduring. The pathogenesis of the condition is thought to include components of immune dysregulation, disruption to microbiota, autoimmunity, abnormality of clotting and endothelial function, and dysfunctional neurological signalling.^3^ It is beyond the scope of this trial to determine the mechanism of action of the REGAIN intervention, but the reduction in fatigue is likely to be multifactorial. Multiple components of the rehabilitation intervention are likely to have contributed to a reduction in fatigue, which is notoriously complex and treatment resistant. However, carefully prescribed and supervised physical activity along with group education and psychological therapies has been shown to have an impact on fatigue in other clinical populations,^5^ albeit not postviral. The complexity of post-covid-19 condition requires that interventions such as REGAIN are adjuvant—that is, they should be combined with appropriate medical treatment targeted at specific symptom clusters as required.

We were conscious of the potential for post-exertional symptom exacerbation further to physical and mental tasks, as seen in people presenting with chronic fatigue.^20^ Our intervention was tailored and individualised to mitigate this risk. We routinely monitored for signs and symptoms of post-exertional symptom exacerbation but did not observe any instances during the trial or follow-up period, indicating that the intervention was well tolerated. Indeed, the intervention was safe and acceptable overall. The safety profile was such that we did not identify any specific symptom clusters that were exacerbated by physical and mental health rehabilitation. In 33 different intervention groups, totalling more than 1000 participant hours of live exercise and support sessions, only one serious adverse event was possibly related to the intervention, two adverse events were definitely related, and two were probably related.

The REGAIN trial population was severely affected by post-covid-19 condition. At baseline, 43% reported a case level mental health disorder, scoring above accepted clinical thresholds for one or more of anxiety, depression, or post-traumatic stress disorder. Moreover, 38% of participants were unable to work owing to post-covid-19 condition. In addition to improvements in the PROMIS depression subscore, we also witnessed a clinically meaningful reduction in severity of post-traumatic stress disorder with the REGAIN intervention compared with usual care, which was sustained at 12 months. Although the severity of post-traumatic stress disorder reduced in both groups, the magnitude of improvement in the REGAIN intervention group was twofold greater. This is an important finding given the high levels of post-traumatic stress disorder witnessed in this population, and the known impact of this on health related quality of life and social and economic productivity.^27^ Despite observing a reduction in the PROMIS depression subscore at three months, we did not observe a statistically greater effect of the REGAIN intervention in our other measure of depression (HADS). It might be that HADS depression score is less sensitive to change than the PROMIS subscore in post-covid-19 condition. However, both measures indicated a significantly greater effect in favour of the REGAIN intervention at 12 months.

Adherence to the REGAIN intervention was similar to supervised exercise rehabilitation programmes in other clinical conditions.^28^ ^29^ ^30^ Nearly half (47%) of participants attended the initial one-to-one session in addition to at least four of six support sessions and five of eight exercise sessions (full adherence), which resulted in a measurable effect on the outcome. In the complier average causal effect model compared with the intention-to-treat model, there was a 40% greater difference between groups in favour of the REGAIN intervention at three months. Adherence to rehabilitation interventions is known to affect outcomes, with a dose-response relationship seen for both physical and psychological interventions.^29^ ^31^ The larger effect size observed in the complier average causal effect analysis may relate directly to the greater dose of physical and mental health therapies received, or, alternatively, simply to the greater exposure to other participants with similar experiences. Group interaction is a prominent feature in successful lifestyle interventions.^32^

### Comparison with other studies

The REGAIN individually randomised trial was adequately powered to report on the safety and effectiveness of online group physical and mental health rehabilitation for people with post-covid-19 condition at least three months after hospital discharge for covid-19. International guidelines can be informed by this high quality empirical evidence. Although intuitively physical and mental health rehabilitation in a condition characterised by breathlessness, fatigue, reduced physical capacity, and poor emotional wellbeing might be beneficial, no previous empirical data have supported this, particularly in relation to people admitted to hospital with covid-19 or to remotely supervised online interventions. In survivors (n=50) of severe and critical covid-19 (five months post-hospital discharge), 16 weeks of semi-supervised home based rehabilitation was more effective than control for improving physical function and health related quality of life.^33^ A multicentre cohort study (n=582) comparing recovery trajectory after hospital discharge for covid-19 across different care pathways reported improved physical function in two supervised rehabilitation cohorts compared with two cohorts receiving limited or no rehabilitation.^34^ Although informative, data from these studies are not definitive and the many differences with our methodology and participants’ characteristics prevent comparison.

Post-covid-19 condition (with or without hospital admission) is a global public health challenge, with a sizeable effect on societal participation and economic productivity. The REGAIN intervention was delivered online from a single trial hub to a diverse post-covid-19 population across England and Wales. This easily accessible, resource and logistics efficient strategy lends itself to implementation at scale. Physical and mental health recovery is, to an extent, spontaneous in some but not all people with post-covid-19 condition.^26^ Our trial shows that the REGAIN intervention can aid short term and long term recovery in this population.

### Strengths and limitations of this study

The REGAIN intervention was co-created by our patient partners with post-covid-19 condition alongside a multidisciplinary clinical and academic stakeholder group.^8^ Although the content and delivery of the REGAIN intervention was individualised, the programme was sufficiently standardised and thus reproducible, aided by the intervention team being located in a single trial hub supported by manuals for practitioners and participants, regular supervision, and quality assurance. Online delivery ensured accessibility for participants who would otherwise not have been able to take part in centre based rehabilitation programmes because of poor heath, costs, transport, and time pressures. Recruitment using a nationwide (England and Wales) database to identify suitable participants ensured invitations could be sent to areas of high disease burden, thus targeting hard to reach groups and communities. Our trial was individually randomised, and follow-up at the primary outcome time point exceeded 80%, ensuring sufficient statistical power. We can be confident that our primary outcome analysis is robust. The pragmatic nature of a complex intervention delivered by an NHS and community clinical (not research) team ensures high external validity. Assessments of outcomes were completed by participants almost exclusively online, and the data were stored in a bespoke online database to which the research team did not have access. Outcome assessor bias was therefore negligible.

Limitations include the inability of trial participants or practitioners delivering the intervention to be masked to treatment allocation. As a threshold for clinically meaningful change in the PROPr score has not yet been firmly established, we used existing recommendations, which are under investigation.^13^ Nevertheless, the trend towards the benefit of the REGAIN intervention was consistent for most of the outcome measures, indicating a tangible effect. Given that our usual care group received arguably more intervention (ie, best practice usual care) than might have been offered in clinical practice, it is possible that the true effect of the REGAIN intervention is masked, as reported previously in other clinical trials.^35^ As such, the true effect may possibly be larger than reported when compared with no treatment. We report short term and long term clinical effectiveness, and we will report on cost effectiveness elsewhere. Despite the trial and intervention materials being translated into multiple languages, we only recruited one participant through our non-English speaking pathway. Only 11% of the trial population were of non-white ethnicity, which may limit generalisability. Translation of materials appears to be insufficient to attract participants from minority ethnic groups. Targeted work at the community level is likely needed to fulfil this goal. The effectiveness of the REGAIN intervention does, however, mean it can be adapted specifically for delivery in hard-to-reach communities.

### Conclusions

Among adults with post-covid-19 condition at least three months after hospital discharge for covid-19, an individualised online, group physical and mental health rehabilitation intervention improved overall heath related quality of life more than usual care at three and 12 months post-randomisation. REGAIN is an accessible, resource efficient programme that can be delivered at scale, contributing to a reduction in the global burden of post-covid-19 condition.

**What is already known on this topic**

Post-covid-19 condition (long covid) includes many debilitating symptoms, such as breathlessness, fatigue, pain, reduced physical capacity, and poor emotional wellbeing
Exercise and psychological rehabilitation can support recovery in clinical conditions with similar symptom profiles
Rehabilitation programmes may help people with post-covid-19 condition; however, no empirical data exist to indicate benefit or harm, and existing literature exclusively reports consensus recommendations

**What this study adds**

In adults with post-covid-19 condition, at least three months after hospital discharge for covid-19, an eight week, live, online, home based, supervised group rehabilitation programme (REGAIN) was well tolerated and led to sustained improvements in health related quality of life at three months and one year compared with usual care
High quality evidence from the REGAIN randomised controlled trial confirmed the clinical benefit and lack of harm of online physical and mental health rehabilitation for adults with post-covid-19 condition at least three months after hospital discharge for covid-19
These findings should assist clinicians in the treatment of this complex condition

## Data Availability

Data are available on reasonable request from wctudataaccess@warwick.ac.uk.

## Web extra

Extra material supplied by authors

## Notes

Contributors: GM and MU conceived the trial. GM, HS, JB, BS, DM, JY, CJ, BL, CJi, JM, RL, KS, and MU are grant holders. All authors contributed to the trial protocol, implementation, and data interpretation: GM, SE, and PH (clinical exercise rehabilitation); MU and JB (clinical trials); HS, SP, and CA (health psychology/behaviour change); BS (psychiatry); JY, DM, and CJ (critical care medicine and rehabilitation); BL (respiratory medicine); RL, CJi, and MR (statistics); JM and HN (health economics); KS and VN (process evaluation, qualitative); and JS and SA (trial management). GM wrote the initial draft of the manuscript supported by MU, JB, RL, and MR. GM is the guarantor. All authors read, revised, and approved the final version of the manuscript. The corresponding author attests that all listed authors meet authorship criteria and that no others meeting the criteria have been omitted.

GM (the manuscript’s guarantor) affirms that the manuscript is an honest, accurate, and transparent account of the study being reported; that no important aspects of the study have been omitted; and that any discrepancies from the study as originally planned (and, if relevant, registered) have been explained.

Dissemination to participants and related patient and public communities: Dissemination of trial findings suitable for a lay audience will be conducted with people affected by post-covid-19 condition as integral members of the team through social media, newsletter, website, and professional and lay conferences.

Provenance and peer review: Not commissioned; externally peer reviewed.
