# Supplementary material for: Clinical effectiveness of an online supervised group physical and mental health rehabilitation programme for adults with post-covid-19 condition (REGAIN study): multicentre randomised controlled trial
Source: BMJ. 2024 Feb 7;384:e076506. doi: 10.1136/bmj-2023-076506 (PMC11134408; doi:10.1136/bmj-2023-076506)
Supplement: Supplementary file 1 — Supplementary information: Additional material, figures, and tables [file mcgg076506.ww.pdf]

Rehabilitation Exercise and psycholoGical support After covid-19 InfectioN (REGAIN): a multi-centre randomised controlled trial.

Supplementary material

	Page
Supplementary methods	2
Supplementary figures	3
Supplementary tables	4
CONSORT checklist	27
TIDieR checklist	31
Protocol	32
Statistical analysis plan	88

Supplementary methods

Identification and management of case-level mental health disorder

Outcome measures at baseline and three months included questionnaires to assess emotional well-being and mental health:

1) Hospital anxiety and depression scale (HADS)
2) Post-traumatic stress syndrome (PTSD) Impact of Event Scale-Revised (IES-R)
3) IES-6 – a six-item subscale of the IES-R

Conscious of the likely considerable mental health pathology in the hospitalised post-COVID syndrome population, we chose to implement cut-off points to identify case-level mental health disorder. We did this for two reasons:

1) to stratify the minimisation algorithm
2) to allow us to inform participants' general practitioner of any relevant clinical findings.

Cut-off points were derived from existing literature in consultation with the trial health psychologist and psychiatrist. The following were considered suggestive of case-level mental health disorder:

1) HADS anxiety sub-score ≥11/21
2) HADS depression sub-score ≥11/21
3) IES-6 score ≥11/24

For the HADS anxiety and depression scores individually, we used a cut-off point of 11, which is widely reported to be indicative of moderate anxiety or depression.[1]

In accordance with the literature, we used the full 22-item IES-R as an outcome measure for PTSD severity.[2] However, in the absence of an established cut-off point for case-level mental health disorder with the IES-R, we used the IES-6 for this purpose, as recommended in the literature. A mean score of 1.75 on the six questions of the IES-6 (total =10.5 (rounded to 11)) has previously been shown to identify PTSD in survivors of acute respiratory distress disorder (ARDS).[3]

We informed general practitioners in writing of participants whose baseline (or follow-up) scores met any of the pre-defined criteria for case level mental health. However, participants who met any of these criteria continued in the trial.

In line with our statistical analysis plan, HADs anxiety and HADS depression were analysed as continuous rather than categorical outcomes. We have analysed the PTSD IES-r as a continuous outcome measure. However in response to reviewer feedback, we have, to aid interpretation of any effects observed, added categorical data to our results table. We have not done any additional statistical analyses on these data as this was not in our statistical analysis plan.

References

1. Stern AF. The Hospital Anxiety and Depression Scale. *Occupational Medicine* 2014; **64**(5): 393-4.
2. Hosey MM, Bienvenu OJ, Dinglas VD, et al. The IES-R remains a core outcome measure for PTSD in critical illness survivorship research. *Critical Care* 2019; **23**(1): 362.
3. Hosey MM, Leoutsakos J-MS, Li X, et al. Screening for posttraumatic stress disorder in ARDS survivors: validation of the Impact of Event Scale-6 (IES-6). *Critical Care* 2019; **23**(1): 276.

Supplementary figures

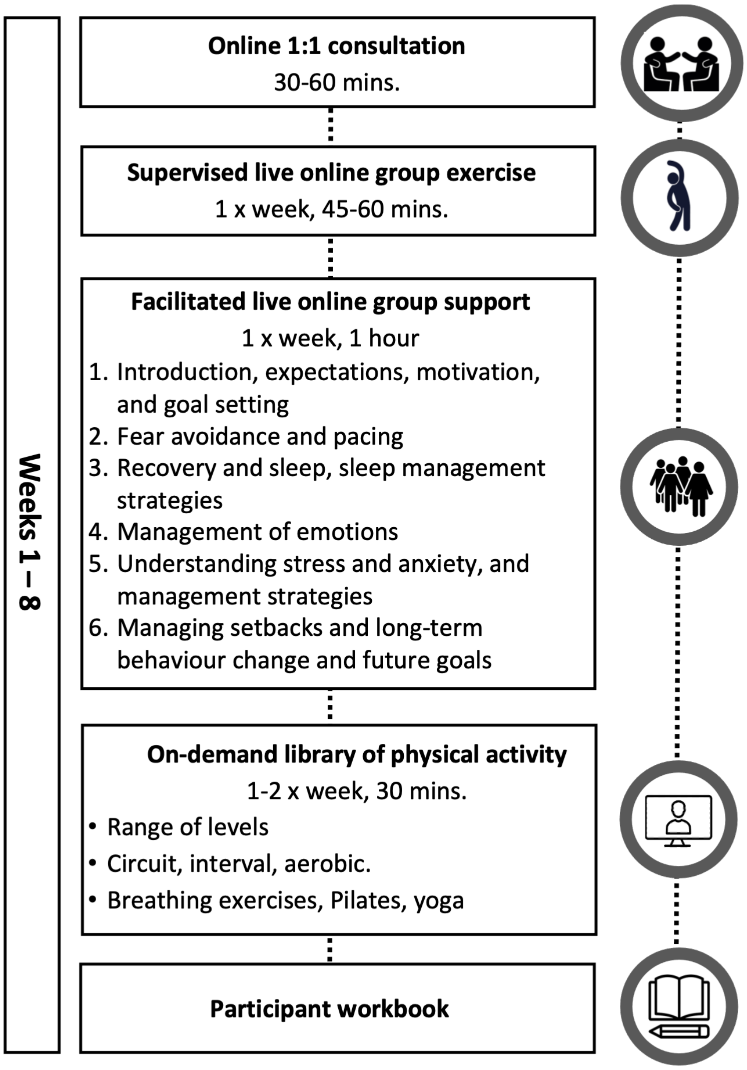

Figure S1. Final format and components of the 'Rehabilitation Exercise and psycholoGical support After covid-19 InfectioN' (REGAIN) intervention.

Supplementary tables

Table S1: Participants by randomisation strata

	Intervention<br>298 (50.9%)	Usual care<br>287 (49.1%)	Total<br>585
**Age, years**			
<65	225 (75.5%)	216 (75.3%)	441 (75.4%)
≥65	73 (24.5%)	71 (24.7%)	144 (24.6%)
**Hospital care**			
ICU/HDU	102 (34.2%)	99 (34.5%)	201 (34.4%)
Ward	196 (65.8%)	188 (65.5%)	384 (65.6%)
**Case level mental health disorder**			
Yes	128 (42.9%)	123 (42.9%)	251 (42.9%)
No	170 (57.1%)	164 (57.1%)	334 (57.1%)

Values are number (%). Data show randomisation allocation is 1.04:1. ICU/HDU, intensive care unit/high dependency unit.

Table S2: Baseline demographics

	Intervention	Usual care	TOTAL
**Age (years)**			
N	**298**	**287**	**585**
Mean (SD)	56.1 (12.1)	56.2 (12.3)	56.1 (12.2)
Median (IQR)	56.0 (48.0, 64.0)	56.0 (48.0, 65.0)	56.0 (48.0, 64.0)
<65	225 (75.5%)	216 (75.3%)	441 (75.4%)
≥65	73 (24.5%)	71 (24.7%)	144 (24.6%)
Missing	0 (0.0%)	0 (0.0%)	0 (0.0%)
**Gender**			
Male	136 (45.6%)	144 (50.2%)	280 (47.9%)
Female	162 (54.4%)	143 (49.8%)	305 (52.1%)
Other	0 (0.0%)	0 (0.0%)	0 (0.0%)
Prefer not to say	0 (0.0%)	0 (0.0%)	0 (0.0%)
Missing	0 (0.0%)	0 (0.0%)	0 (0.0%)
**Ethnicity**			
White	265 (88.9%)	252 (87.8%)	517 (88.4%)
Black Caribbean	3 (1.0%)	6 (2.1%)	9 (1.5%)
Black African	6 (2.0%)	3 (1.1%)	9 (1.5%)
Black Other	1 (0.3%)	0 (0.0%)	1 (0.2%)
Indian	6 (2.0%)	9 (3.1%)	15 (2.6%)
Pakistani	1 (0.3%)	2 (0.7%)	3 (0.5%)
Bangladeshi	0 (0.0%)	0 (0.0%)	0 (0.0%)
Chinese	1 (0.3%)	0 (0.0%)	1 (0.2%)
Prefer not to say	2 (0.7%)	2 (0.7%)	4 (0.7%)
Mixed- White/Black Caribbean	1 (0.3%)	0 (0.0%)	1 (0.2%)
Mixed – White/Black African	0 (0.0%)	1 (0.4%)	1 (0.2%)
Mixed – White/Asian	3 (1.0%)	1 (0.4%)	4 (0.7%)
Other	9 (3.0%)	11 (3.8%)	20 (3.4)
Missing	0 (0.0%)	0 (0.0%)	0 (0.0%)
**Body mass index** (kg/m^2)			
Mean (SD)	33.0 (7.7)	32.8 (8.0)	32.9 (7.8)
Median (IQR)	31.6 (27.7, 37.5)	31.1 (27.8, 36.7)	31.4 (27.8, 37.1)
Underweight (<18.5)	0 (0.0%)	1 (0.4%)	1 (0.2%)
Healthy weight (18.5 to 24.9)	38 (12.8)	38 (13.2%)	76 (13.0%)

	Intervention	Usual care	TOTAL
Overweight (25 to 29.9)	84 (28.2%)	75 (26.1%)	159 (27.2%)
Obese (30 to 39.9)	176 (59.1%)	173 (60.3%)	349 (59.7%)
Missing	0 (0.0%)	0 (0.0%)	0 (0.0%)
**Smoking status**			
Current smoker	8 (2.7%)	4 (1.4%)	12 (2.1%)
Ex-smoker	118 (39.6%)	121 (42.2%)	239 (40.8%)
Never smoked	172 (57.7%)	162 (56.4%)	334 (57.1%)
Missing	0 (0.0%)	0 (0.0%)	0 (0.0%)
**Further training after school**			
No	68 (22.8%)	54 (18.8%)	122 (20.9%)
Yes	230 (77.2%)	233 (81.2%)	463 (79.1%)
Missing	0 (0.0%)	0 (0.0%)	0 (0.0%)
If Yes:			
Qualification obtained through work*†	157 (52.7%)	163 (56.8%)	320 (54.7%)
Degree*†	115 (38.6%)	99 (34.5%)	214 (36.6%)
Other non-degree qualification*†	175 (58.7%)	177 (61.7%)	352 (60.2%)
**Employment status**			
Full-time work	160 (53.7%)	162 (56.5%)	322 (55.0%)
Part-time work	45 (15.1%)	37 (12.9%)	82 (14.0%)
Full time education	2 (0.7%)	0 (0.0%)	2 (0.3%)
Part-time education	1 (0.3%)	0 (0.0%)	1 (0.2%)
Unemployed	10 (3.4%)	4 (1.4%)	14 (2.4%)
Retired	49 (16.4%)	62 (21.6%)	111 (19.0%)
Unable to work for personal health reasons	27 (9.1%)	20 (7.0%)	47 (8.0%)
Other	4 (1.3%)	2 (0.7%)	6 (1.0%)
Missing	0 (0.0%)	0 (0.0%)	0 (0.0%)
**Unable to work because of health problems following COVID-19**			
Yes	125 (41.9%)	97 (33.8%)	222 (37.9%)
No	173 (58.1%)	190 (66.2%)	363 (62.1%)
Missing	0 (0.0%)	0 (0.0%)	0 (0.0%)
**Comorbidities:**			
**Heart or circulation**			
Yes	77 (25.8%)	99 (34.5%)	176 (30.1%)
No	221 (74.2%)	188 (65.5%)	409 (69.9)
Missing	0 (0.0%)	0 (0.0%)	0 (0.0%)
If yes:			
Taking medication	56 (72.7%)	76 (76.8%)	132 (75.0%)
Not taking medication	21 (27.3%)	23 (23.2%)	44 (25.0%)
Missing	0 (0.0%)	0 (0.0%)	0 (0.0%)
**Chest or breathing**			
Yes	226 (75.8%)	218 (76.0%)	444 (75.9%)
No	72 (24.2%)	69 (24.0%)	141 (24.1%)
Missing	0 (0.0%)	0 (0.0%)	0 (0.0%)
If yes:			
Taking medication	119 (52.7%)	105 (48.2%)	224 (50.5%)
Not taking medication	107 (47.4%)	113 (51.8%)	220 (49.6%)
Missing	0 (0.0%)	0 (0.0%)	0 (0.0%)
**Kidney or bladder**			
Yes	50 (16.8%)	53 (18.5%)	103 (17.6%)
No	248 (83.2%)	234 (81.5%)	482 (82.4%)
Missing	0 (0.0%)	0 (0.0%)	0 (0.0%)
If yes:			
Taking medication	27 (54.0%)	25 (47.2%)	52 (50.5%)
Not taking medication	23 (46.0%)	28 (52.8%)	51 (49.5%)
Missing	0 (0.0%)	0 (0.0%)	0 (0.0%)

	Intervention	Usual care	TOTAL
**Stomach, bowel or abdomen**			
Yes	93 (31.2%)	83 (28.9%)	176 (30.1%)
No	205 (68.8%)	204 (71.1%)	409 (69.9%)
Missing	0 (0.0%)	0 (0.0%)	0 (0.0%)
If yes:			
Taking medication	60 (64.5%)	54 (65.1%)	114 (64.8%)
Not taking medication	33 (35.5%)	29 (34.9%)	62 (35.2%)
Missing	0 (0.0%)	0 (0.0%)	0 (0.0%)
**Endocrine**			
Yes	92 (30.9%)	83 (28.9%)	175 (29.9%)
No	206 (69.1%)	204 (71.1%)	410 (70.1%)
Missing	0 (0.0%)	0 (0.0%)	0 (0.0%)
If yes:			
Taking medication	79 (85.9%)	62 (74.7%)	141 (80.6%)
Not taking medication	13 (14.1%)	21 (25.3%)	34 (19.4%)
Missing	0 (0.0%)	0 (0.0%)	0 (0.0%)
**Musculoskeletal**			
Yes	143 (48.0%)	132 (46.0%)	275 (47.0%)
No	155 (52.0%)	155 (54.0%)	310 (53.0%)
Missing	0 (0.0%)	0 (0.0%)	0 (0.0%)
If yes:			
Taking medication	74 (51.8%)	71 (53.8%)	145 (52.7%)
Not taking medication	69 (48.3%)	61 (46.2%)	130 (47.3%)
Missing	0 (0.0%)	0 (0.0%)	0 (0.0%)
**Brain or nervous system**			
Yes	67 (22.5%)	67 (23.3%)	134 (22.9%)
No	231 (77.5%)	220 (76.7%)	451 (77.1%)
Missing	0 (0.0%)	0 (0.0%)	0 (0.0%)
If yes:			
Taking medication	27 (40.3%)	31 (46.3%)	58 (43.3%)
Not taking medication	40 (59.7%)	36 (53.7%)	76 (56.7%)
Missing	0 (0.0%)	0 (0.0%)	0 (0.0%)
**Blood or clotting**			
Yes	48 (16.1%)	62 (21.6%)	110 (18.8%)
No	250 (83.9%)	225 (78.4%)	475 (81.2%)
Missing	0 (0.0%)	0 (0.0%)	0 (0.0%)
If yes:			
Taking medication	43 (89.6%)	55 (88.7%)	98 (89.1%)
Not taking medication	5 (10.4%)	7 (11.3%)	12 (10.9%)
Missing	0 (0.0%)	0 (0.0%)	0 (0.0%)
**Other health problem**			
Yes	117 (39.3%)	123 (42.9%)	240 (41.0%)
No	181 (60.7%)	164 (57.1%)	345 (59.0%)
Missing	0 (0.0%)	0 (0.0%)	0 (0.0%)
**Admission to ICU/HDU**			
Yes	102 (34.2%)	99 (34.5%)	201 (34.4%)
No	196 (65.8%)	188 (65.5%)	384 (65.6%)
Missing	0 (0.0%)	0 (0.0%)	0 (0.0%)
**Time since discharge (days)**			
Mean (SD)	331 (151)	314 (137)	323 (144)
Median (IQR)	316 (205, 440)	294 (199, 419)	307 (201, 433)
Range	93, 901	115, 732	93, 901

Values mean (SD, median (IQR) or number (%) as appropriate. *Percentages may add up to >100% as patients may fall into multiple categories; †The denominator is the number of patients that had an educational qualification after school. ICU/HDU, intensive care unit/high dependency unit.

Table S3: Baseline outcome data

	Intervention	Usual care	TOTAL
**PROMIS 29+2 Profile v2.1 (PROPr)** (A higher score reflects better health; range -0.022 to 1.0)			
N	298	287	585
Mean (SD)	0.201 (0.166)	0.198 (0.165)	0.199 (0.165)
Median (IQR)	0.177 (0.072, 0.294)	0.159 (0.067, 0.309)	0.168 (0.068, 0.301)
Missing	0	0	0
Range	-0.020, 0.820	-0.019, 0.733	-0.020, 0.820
**EQ5D-5L Index Score** (A higher score reflects better quality of life; range 0.224 to 1.0)			
N	298	287	585
Mean (SD)	0.548 (0.274)	0.551 (0.247)	0.550 (0.261)
Median (IQR)	0.633 (0.408, 0.735)	0.612 (0.407, 0.721)	0.624 (0.407, 0.728)
Missing	0	0	0
Range	-0.436, 1.00	-0.326, 1.00	-0.436, 1.00
**EQ5D-5L VAS -** (A higher score reflects overall health; range 0 to 100)			
N	298	287	585
Mean (SD)	55.6 (19.7)	53.2 (19.9)	54.4 (19.8)
Median (IQR)	56.5 (43.0, 70.0)	50.0 (40.0, 70.0)	53.0 (40.0, 70.0)
Missing	0	0	0
Range	2, 96	5, 100	2, 100
**PTSD Symptom Severity (IES-R)** (A higher score reflects higher severity symptoms; range 0 to 88)			
N	298	286	584
Mean (SD)	30.3 (19.7)	31.0 (19.7)	30.6 (19.7)
Median (IQR)	27.0 (15.0, 44.0)	29.0 (14.0, 46.0)	28.0 (14.0, 45.0)
Missing	0	1	1
Range	0.0, 86.0	0.0, 81.0	0.0, 86.0
Score<11	56 (18.8%)	54 (18.8%)	110 (18.8%)
Score ≥11	242 (81.2%)	233 (81.2%)	475 (81.2%)
**HADS anxiety** (A higher score reflects greater level of anxiety; range 0 to 21)			
N	298	287	585
Mean (SD)	9.0 (5.3)	9.4 (4.9)	9.2 (5.1)
Median (IQR)	9.0 (5.0, 13.0)	10.0 (6.0, 13.0)	9.0 (5.0, 13.0)
Missing	0	0	0
Range	0.0, 21.0	0.0, 21.0	0.0, 21.0
Score <11	176 (59.1%)	167 (58.2%)	343 (58.6%)
Score ≥11	122 (40.9%)	120 (41.8%)	242 (41.4%)
**HADS depression** (A higher score reflects greater level of depression; range 0 to 21)			
N	298	287	585
Mean (SD)	8.8 (4.7)	9.0 (4.5)	8.9 (4.6)
Median (IQR)	9.0 (5.0, 12.0)	9.0 (6.0, 13.0)	9.0 (5.0, 12.0)
Missing	0	0	0
Range	0.0, 21.0	0.0, 20.0	0.0, 21.0
Score <11	197 (66.1%)	178 (62.0%)	375 (64.1%)
Score ≥11	101 (33.9%)	109 (38.0%)	210 (35.9%)
**PROMIS dyspnoea severity short form (10-item)** (A higher score reflects greater level of breathlessness; population mean=50 and SD=10)			
N	295	285	580
Mean (SD)	55.1 (8.7)	55.4 (8.6)	55.2 (8.7)
Median (IQR)	55.2 (49.5, 61.4)	55.6 (50.2, 61.0)	55.5 (49.6, 61.3)
Missing	3	2	5
Range	31.7, 77.8	31.7, 77.8	31.7, 77.8

	Intervention	Usual care	TOTAL
**Cognitive function (PROMIS Neuro-QoL) (8 items)** (A higher score reflects better cognitive function; population mean=50 and SD=10)			
N	297	287	584
Mean (SD)	39.4 (9.2)	39.0 (9.2)	39.2 (9.2)
Median (IQR)	39.3 (33.0, 44.6)	38.8 (32.1, 44.6)	38.9 (32.6, 44.6)
Missing	1	0	1
Range	17.7, 64.2	17.7, 64.2	17.7, 64.2
**Overall health:** compared to three months ago			
Much better now	21 (7.1%)	23 (8.0%)	44 (7.5%)
Somewhat better now	83 (28.0%)	70 (24.4%)	153 (26.2%)
About the same	116 (38.9%)	126 (43.9%)	242 (41.4%)
Somewhat worse now	57 (19.1%)	43 (15.0%)	100 (17.1%)
Much worse now	20 (6.7%)	23 (8.0%)	43 (7.4%)
Missing	1 (0.3%)	2 (0.7%)	3 (0.5%)

Values are mean (SD), median (IQR), number (%) as appropriate. PROPr, PROMIS preference score; VAS, visual analogue scale; PTSD, post-traumatic stress; IES-R, index of event scale – revised; HADS, hospital anxiety and depression score; PROMIS, patient-reported outcomes measurement information system. A score ≥11 on any of the PTSD-IES-6, HADS anxiety, or HADS depression scales was the threshold for case level mental health disorder.

Table S4: Overall summary of withdrawals by treatment arm*

Withdrawal from	Intervention	Usual care	TOTAL
**Total RANDOMISED**	**298 (100%)**	**287 (100%)**	**585 (100%)**
Intervention only	50 (16.8%)	13 (4.5%)	63 (10.8%)
Follow-up questionnaires	28 (9.4%)	16 (5.6%)	44 (7.5%)
GP medical records consent	16 (5.4%)	10 (3.5%)	26 (4.4%)
Interview consent	29 (9.7%)	16 (5.6%)	45 (7.7%)
**Complete withdrawal/ Withdrawal from trial**	13 (4.4%)	5 (1.7%)	**18 (3.1%)**

Values are number (%). *% out of total randomised. Some participants have more than one withdrawal status.

Table S5: Reasons for withdrawal from trial (complete withdrawals)*

Reasons	Intervention	Usual care	TOTAL
Participant does not have time to take part/too burdensome	3 (23.1%)	1 (20.0%)	4 (22.2%)
Participant does not believe the study will benefit them	0 (0.0%)	0 (0.0%)	0 (0.0%)
Participant had a preference for the opposite study arm	0 (0.0%)	0 (0.0%)	0 (0.0%)
Withdrawal was practitioner decision	0 (0.0%)	0 (0.0%)	0 (0.0%)
No reason given	0 (0.0%)	0 (0.0%)	0 (0.0%)
Other	10 (76.9%)	4 (80.0%)	14 (77.8%)
Missing	0 (0.0%)	0 (0.0%)	0 (0.0%)
**Total**	**13 (100%)**	**5 (100%)**	**18 (100%)**

Values are number (%). *% out of total withdrawals

Table S6: Follow-up rates

	Intervention			Usual care		
**Time-point**	**Number of participants due questionnaire**	**Number of participants returned questionnaire**	**Reason**	**Number of participants due questionnaire**	**Number of participants returned questionnaire**	**Reason**
Baseline	298	298 (100%*)	Missing (being chased): 0	287	287 (100%)	Missing (being chased): 0
			Death: 0			Death: 0
			Withdrawal: 0			Withdrawal: 0
			Lost to follow-up (non-respondent): 0			Lost to follow-up (non-respondent): 0
3 months	298	237 (79.5%)	Missing (being chased): 0	287	250 (87.1%)	Missing (being chased): 0
			Death: 0			Death: 0
			Withdrawal: 9**			Withdrawal: 4
			Lost to follow-up (non-respondent): 52			Lost to follow-up (non-respondent): 33
6 months	298	226 (75.8%)	Missing (being chased): 0	287	239 (83.3%)	Missing (being chased): 0
			Death: 0			Death: 1
			Withdrawal: 10			Withdrawal: 4
			Lost to follow-up (non-respondent): 62			Lost to follow-up (non-respondent): 43
12 months	298	216 (72.5%)	Missing (being chased): 0	287	226 (78.7%)	Missing (being chased): 0
			Death: 2			Death: 2
			Withdrawal: 13			Withdrawal: 5
			Lost to follow-up (non-respondent): 67			Lost to follow-up (non-respondent): 54

Values are number (%). *% out of follow-up due; **withdrawals are counted cumulatively: missing cases are not counted in the calculation of follow-up completion percentages.

Table S7: Summary of compliance with trial intervention

	**Intervention N=298**	**Usual care N=287**	**Total N=585**
**Fully complied**	141 (47.3%)	259 (90.2%)	400 (68.4%)
**Partially complied**	117 (39.3%)	-	117 (20.0%)
**Did not receive**	40 (13.4%)	28 (9.8%)	68 (11.6%)
Reasons *DNA*	6	6	12
declined	26	13	39
Non-responder	8	9	17

Values are number (%). Full compliance is defined as completion of the initial assessment, at least 4/6 of the live support sessions and at least 5/8 of the live exercise sessions. Partial compliance is defined as completion of the initial assessment, and <4/6 of the live support sessions and at <5/8 of the live exercise sessions.

Table S8: Summary of active intervention compliance

	**Intervention N=298**	**Usual care N=287**
Time from randomisation to initial 1:1 consultation (days)		
N	258	259
Mean (SD)	20.0 (15.2)	30.0 (18.5)
Median (IQR)	16.5 (8.0, 28.0)	27.0 (14.0, 43.0)
Didn't attend first one-to-one	40	28
Missing	0	0
Time from randomisation to first live group session (days)		
N	234	0
Mean (SD)	35.4 (23.6)	-
Median (IQR)	32.0 (19.0, 43.0)	-
Didn't attend first live session	64	-
Missing	0	-
Live exercise session attendance (sessions)		
N	258	0
Mean (SD)	4.4 (2.8)	-
Median (IQR)	5.0 (2.0, 7.0)	-
Attended none	45	-
Attended 1-5 sessions	97	-
Attended 6+ sessions	116	-
Attended 8 sessions	37	-
Missing	40	-
Exercise session attendance (On demand sessions)		
N	258	0
Mean (SD)	0.7 (2.4)	-
Median (IQR)	0.0 (0.0, 0.0)	-
Watched 0 sessions	206	-
Watched 1-7 home sessions	46	-
Watched 8-16 home sessions	4	-
Watched 17+ home sessions	2	-
Missing	40	-
Psychological support session attendance (sessions)		
N	258	0
Mean (SD)	4.1 (2.1)	-
Median (IQR)	5.0 (2.0, 6.0)	-
Attended none	27	-
Attended 1-3 sessions	51	-
Attended at least 4 sessions	180	-
Attended all 6 sessions	95	-
Missing	40	-
Group size at randomisation		
N	33	0
Mean (SD)	7.5 (2.3)	-
Median (IQR)	7.0 (6.0, 9.0)	-
Missing	0	-

Values are mean (SD), median (IQR) as appropriate.

Table S9: Primary outcome (HRQoL score and sub-scores) results at 3 months follow-up

	Intervention	Usual care	TOTAL	Unadjusted estimate (95% CI); p-value	Adjusted estimate* (95% CI); p-value
**PROMIS 29+2 Profile v2.1 (PROPr) (HRQoL)**					
N	237	248	485		
Mean (SD)	0.265 (0.183)	0.234 (0.181)	0.249 (0.183)		
Median (IQR)	0.241 (0.126, 0.371)	0.214 (0.096, 0.338)	0.224 (0.109, 0.362)	0.030 (-0.004, 0.064); 0.088	0.028 (0.006, 0.050); 0.015
Missing**	61	37	98		
Missing***	0	2	2		
**PROMIS Emotional distress – Anxiety sub score** (a higher score indicate more severe symptoms)					
N	237	249	486		
Mean (SD)	55.8 (10.2)	57.4 (9.2)	56.6 (9.7)		
Median (IQR)	56.0 (48.5, 63.5)	58.0 (51.7, 63.5)	57.5 (51.4, 63.5)	1.662 (-0.091, 3.414); 0.063	1.197 (-0.170, 2.564); 0.085
Missing**	61	37	98		
Missing***	0	1	1		
**PROMIS Emotional distress – Depression sub score** (a higher score indicate more severe symptoms)					
N	237	248	485		
Mean (SD)	53.3 (10.5)	55.3 (9.7)	54.3 (10.1)		
Median (IQR)	54.1 (41.0, 62.2)	56.1 (48.9, 62.2)	55.5 (41.0, 62.2)	1.969 (0.055, 3.882); 0.044	1.386 (0.059, 2.713); 0.041
Missing**	61	37	98		
Missing***	0	2	2		
**PROMIS Fatigue sub score** (a higher score indicate more severe symptoms)					
N	237	248	485		
Mean (SD)	57.9 (9.2)	60.6 (9.5)	59.3 (9.4)		
Median (IQR)	57.2 (51.0, 64.7)	60.8 (53.2, 66.6)	59.2 (51.0, 64.7)	2.636 (0.952, 4.319); 0.002	2.499 (1.189, 3.809); <0.001
Missing**	61	37	98		
Missing***	0	2	2		
**PROMIS Sleep disturbance sub score** (a higher score indicate more severe symptoms)					
N	237	248	485		
Mean (SD)	55.5 (8.4)	56.6 (9.1)	56.1 (8.8)	1.185 (-0.385, 2.754); 0.138	0.863 (-0.271, 1.997); 0.135
Median (IQR)	55.5 (51.1, 61.4)	57.2 (51.3, 62.4)	56.0 (51.1, 61.9)		

	Intervention	Usual care	TOTAL	Unadjusted estimate (95% CI); p-value	Adjusted estimate* (95% CI); p-value
Missing**	61	37	98		
Missing***	0	2	2		
**PROMIS Pain interference sub score** (higher score indicate more severe symptoms)					
N	237	248	485	2.134 (0.330, 3.938); 0.021	1.801 (0.497, 3.105); 0.007
Mean (SD)	56.8 (10.1)	59.0 (9.9)	57.9 (10.1)		
Median (IQR)	56.9 (52.0, 63.6)	60.0 (53.9, 66.7)	57.5 (53.9, 65.2)		
Missing**	61	37	98		
Missing***	0	2	2		
**PROMIS Pain intensity** (higher score indicate more severe symptoms)					
N	237	247	484	0.392 (-0.085, 0.869); 0.107	0.320 (-0.034, 0.675); 0.076
Mean (SD)	3.8 (2.7)	4.2 (2.6)	4.0 (2.7)		
Median (IQR)	4.0 (2.0, 6.0)	4.0 (2.0, 7.0)	4.0 (2.0, 6.0)		
Missing**	61	37	98		
Missing***	0	3	3		
**PROMIS Physical function sub score** (higher score indicates better health)					
N	237	249	486	0.082 (-1.301, 1.465); 0.906	0.495 (-0.357, 1.346); 0.253
Mean (SD)	40.3 (7.6)	40.2 (7.6)	40.2 (7.6)		
Median (IQR)	40.2 (35.3, 45.1)	38.6 (34.9, 43.3)	39.6 (35.3, 43.7)		
Missing**	61	37	98		
Missing***	0	1	1		
**PROMIS Social roles and activities sub score** (higher score indicates better health)					
N	237	248	485	0.253 (-1.285, 1.791); 0.745	0.163 (-0.960, 1.286); 0.774
Mean (SD)	44.3 (8.6)	44.1 (8.5)	44.2 (8.5)		
Median (IQR)	44.2 (38.6, 49.6)	44.2 (38.5, 50.1)	44.2 (38.6, 49.9)		
Missing**	61	37	98		
Missing***	0	2	2		
**PROMIS Cognitive function abilities sub score** (higher score indicates better health)					
N	237	248	485	-0.477 (-1.857, 0.904); 0.495	-0.159 (-1.272, 0.953); 0.777
Mean (SD)	45.9 (7.4)	46.3 (7.1)	46.1 (7.3)		
Median (IQR)	43.5 (40.5, 50.0)	46.3 (41.0, 50.0)	45.0 (40.5, 50.0)		

	Intervention	Usual care	TOTAL	Unadjusted estimate (95% CI); p-value	Adjusted estimate* (95% CI); p-value
Missing**	61	37	98		
Missing***	0	2	2		

Values are mean (SD), median (IQR) as appropriate. HRQoL, health-related quality of life; PROPr, PROMIS preference score; PROMIS, patient-reported outcomes measurement information system. Missing data are caused by partially missing questionnaire items; *Based on a partially nested heteroscedastic model adjusted for baseline overall health and stratification variables (age, level of hospital, and level of mental health disorder); The therapist effect was included as a random effect to account for partial clustering; ** missing due to lost to follow-up and withdrawals; ***participants who provided their records, but the form was incomplete (fully or partially) and therefore their record couldn't be included in the analysis.

Table S10: Primary outcome (HRQoL score and sub-scores) results at 6 months follow-up

	Intervention	Usual care	TOTAL	Unadjusted estimate (95% CI); p-value	Adjusted estimate* (95% CI); p-value
**PROMIS 29+2 Profile v2.1 (PROPr) (HRQoL)**					
N	225	237	462***	0.029 (-0.008, 0.067); 0.127	0.023 (-0.003, 0.048); 0.081
Mean (SD)	0.274 (0.200)	0.244 (0.200)	0.259 (0.200)		
Median (IQR)	0.244 (0.119, 0.387)	0.209 (0.108, 0.340)	0.225 (0.112, 0.370)		
Missing**	1	3	4		
**PROMIS Emotional distress – Anxiety sub score**					
N	226	238	464	2.186 (0.305, 4.067); 0.023	1.709 (0.323, 3.094); 0.016
Mean (SD)	56.1 (10.7)	58.3 (9.6)	57.2 (10.2)		
Median (IQR)	57.5 (48.1, 63.5)	59.6 (53.8, 65.2)	57.8 (51.4, 63.5)		
Missing**	0	1	1		
**PROMIS Emotional distress – Depression sub score**					
N	226	236	462	2.420 (0.482, 4.358); 0.015	2.048 (0.634, 3.462); 0.005
Mean (SD)	53.6 (10.8)	56.1 (10.2)	54.9 (10.5)		
Median (IQR)	54.2 (41.0, 62.2)	57.1 (48.9, 62.2)	55.9 (41.0, 62.2)		
Missing**	0	3	3		
**PROMIS Fatigue sub score**					
N	226	236	462	1.982 (0.011, 3.954); 0.049	1.629 (0.027, 3.230); 0.046
Mean (SD)	58.1 (10.5)	60.1 (10.2)	59.1 (10.4)		
Median (IQR)	58.9 (48.7, 64.7)	60.8 (53.2, 66.8)	59.8 (51.0, 64.8)		
Missing**	0	3	3		

	Intervention	Usual care	TOTAL	Unadjusted estimate (95% CI); p-value	Adjusted estimate* (95% CI); p-value
**PROMIS Sleep disturbance sub score**					
N	226	236	462	1.717 (0.032, 3.402); 0.046	1.240 (-0.041, 2.521); 0.058
Mean (SD)	55.2 (9.1)	57.0 (8.7)	56.1 (9.0)		
Median (IQR)	54.6 (49.6, 61.4)	57.4 (52.1, 62.6)	56.2 (51.1, 61.9)		
Missing**	0	3	3		
**PROMIS Pain Interference sub score**					
N	226	236	462	1.035 (-0.862, 2.933); 0.282	0.858 (-0.521, 2.237); 0.220
Mean (SD)	56.9 (10.1)	58.0 (9.9)	57.4 (10.0)		
Median (IQR)	56.3 (51.7, 64.2)	57.5 (53.9, 65.5)	57.1 (52.4, 65.2)		
Missing**	0	3	3		
**PROMIS Pain Intensity**					
N	225	236	461	0.401 (-0.099, 0.901); 0.115	0.334 (-0.058, 0.726); 0.094
Mean (SD)	3.7 (2.7)	4.1 (2.8)	3.9 (2.7)		
Median (IQR)	3.0 (2.0, 6.0)	4.0 (2.0, 7.0)	4.0 (2.0, 6.0)		
Missing**	1	3	4		
**PROMIS Physical function sub score**					
N	226	238	464	0.108 (-1.427, 1.644); 0.889	0.446 (-0.475, 1.367); 0.340
Mean (SD)	40.8 (7.7)	40.5 (8.0)	40.7 (7.8)		
Median (IQR)	40.2 (35.4, 45.1)	39.7 (35.3, 43.3)	39.8 (35.3, 45.1)		
Missing**	0	1	1		
**PROMIS Social roles and Activities sub score**					
N	226	236	462	0.166 (-1.474, 1.807); 0.841	-0.046 (-1.246, 1.153); 0.939
Mean (SD)	44.6 (9.1)	44.4 (8.6)	44.5 (8.9)		
Median (IQR)	44.2 (38.6, 49.9)	44.2 (37.2, 50.2)	44.2 (38.5, 50.2)		
Missing**	0	3	3		
**PROMIS Cognitive Function Abilities sub score**					
N	225	236	461	-0.208 (-1.553, 1.137); 0.760	-0.110 (-1.317, 1.097); 0.857
Mean (SD)	46.7 (7.4)	46.9 (7.2)	46.8 (7.3)		
Median (IQR)	46.3 (41.0, 50.0)	46.3 (41.0, 52.0)	46.3 (41.0, 50.0)		
Missing**	1	3	4		

Values are mean (SD), median (IQR) as appropriate. HRQoL, health-related quality of life; PROPr, PROMIS preference score; PROMIS, patient-reported outcomes measurement information system. Missing data are caused by partially missing questionnaire items; *Based on a partially nested heteroscedastic model adjusted for baseline overall health and stratification variables (age, level of hospital, and level of mental health disorder); The therapist effect was included as a random effect to account for

partial clustering; **participants who provided their records, but the form was incomplete (fully or partially) and therefore their record couldn't be included in the analysis; *** includes one participant who died at this timepoint.

Table S11: Primary outcome (HRQoL score and sub-scores) results at 12 months follow-up

	Intervention	Usual care	TOTAL	Unadjusted estimate (95% CI); p-value	Adjusted estimate* (95% CI); p-value
**PROMIS 29+2 Profile v2.1 (PROPr) (HRQoL)**					
N	217	227	444***	0.037 (-0.006, 0.080); 0.090	0.034 (0.006, 0.063); 0.019
Mean (SD)	0.292 (0.22)	0.252 (0.20)	0.272 (0.22)		
Median (IQR)	0.253 (0.124, 0.426)	0.214 (0.091, 0.371)	0.226 (0.101, 0.396)		
Missing**	1	1	2		
**PROMIS Emotional distress – Anxiety sub score**					
N	216	225	441	1.840 (-0.171, 3.851); 0.072	1.394 (-0.077, 2.866); 0.063
Mean (SD)	55.3 (10.9)	57.1 (10.2)	56.2 (10.6)		
Median (IQR)	55.8 (47.9, 63.5)	57.5 (49.0, 63.5)	57.5 (48.1, 63.5)		
Missing**	0	1	1		
**PROMIS Emotional distress – Depression sub score**					
N	215	225	440	2.084 (0.033, 4.136); 0.046	1.676 (0.200, 3.153); 0.026
Mean (SD)	53.0 (10.6)	55.2 (10.4)	54.1 (10.5)		
Median (IQR)	52.1 (41.0, 62.2)	55.9 (41.0, 62.2)	55.3 (41.0, 62.2)		
Missing**	1	1	2		
**PROMIS Fatigue sub score**					
N	215	225	440	2.063 (0.086, 4.040); 0.041	1.825 (0.254, 3.397); 0.023
Mean (SD)	57.3 (10.9)	59.3 (10.0)	58.3 (10.5)		
Median (IQR)	57.1 (48.6, 66.5)	60.7 (51.0, 64.7)	58.9 (48.7, 64.8)		
Missing**	1	1	2		
**PROMIS Sleep disturbance sub score**					
N	215	225	440	1.649 (-0.033, 3.331); 0.055	1.434 (0.159, 2.710); 0.028
Mean (SD)	55.1 (8.4)	56.8 (9.0)	56.0 (8.7)		
Median (IQR)	53.0 (49.6, 59.5)	57.4 (51.4, 63.6)	55.7 (51.1, 62.1)		
Missing**	1	1	2		
**PROMIS Pain Interference sub score**					
N	215	225	440	0.750 (-1.207, 2.707);	0.536 (-0.983, 2.055);

	Intervention	Usual care	TOTAL	Unadjusted estimate (95% CI); p-value	Adjusted estimate* (95% CI); p-value
Mean (SD)	57.5 (10.4)	58.3 (10.3)	57.9 (10.3)	0.449	0.486
Median (IQR)	56.9 (52.0, 65.5)	57.5 (53.9, 66.7)	57.5 (52.4, 66.7)		
Missing**	1	1	2		
**PROMIS Pain Intensity**					
N	215	225	440	0.272 (-0.228, 0.773); 0.283	0.204 (-0.207, 0.614); 0.328
Mean (SD)	3.8 (2.7)	4.0 (2.6)	3.9 (2.6)		
Median (IQR)	3.0 (1.0, 6.0)	4.0 (2.0, 6.0)	4.0 (2.0, 6.0)		
Missing**	1	1	2		
**PROMIS Physical function sub score**					
N	216	225	441	0.340 (-1.30, 1.979); 0.682	0.607 (-0.413, 1.628); 0.241
Mean (SD)	41.1 (8.1)	40.6 (8.1)	40.9 (8.1)		
Median (IQR)	39.8 (35.3, 45.1)	39.8 (34.9, 45.1)	39.8 (35.0, 45.1)		
Missing**	0	1	1		
**PROMIS Social roles and Activities sub score**					
N	215	225	440	0.536 (-1.217, 2.290); 0.545	0.529 (-0.907, 1.964); 0.466
Mean (SD)	45.4 (9.7)	44.9 (8.8)	45.1 (9.2)		
Median (IQR)	44.2 (38.5, 51.8)	44.2 (38.5, 50.2)	44.2 (38.5, 51.8)		
Missing**	1	1	2		
**PROMIS Cognitive Function Abilities sub score**					
N	215	225	440	0.832 (-0.804, 2.468); 0.315	1.120 (-0.275, 2.516); 0.114
Mean (SD)	47.7 (7.6)	46.7 (7.8)	47.2 (7.8)		
Median (IQR)	47.2 (41.0, 53.7)	46.3 (40.5, 52.0)	46.3 (41.0, 53.7)		
Missing**	1	1	2		

Values are mean (SD), median (IQR) as appropriate. HRQoL, health-related quality of life; PROPr, PROMIS preference score; PROMIS, patient-reported outcomes measurement information system. Missing data are caused by partially missing questionnaire items; *Based on a partially nested heteroscedastic model adjusted for baseline overall health and stratification variables (age, level of hospital, and level of mental health disorder); The therapist effect was included as a random effect to account for partial clustering; ** participants who provided their records, but the form was incomplete (fully or partially) and therefore their record couldn't be included in the analysis; *** includes four participants who died at this timepoint.

Table S12: Difference in HRQoL scores from baseline to 3-month follow-up

	Intervention difference	Usual care difference	TOTAL difference
**PROMIS 29+2 Profile v2.1 (PROPr) (HRQoL)**			
N	237	248	485
Mean (SD)	0.06 (0.13)	0.03 (0.13)	0.04 (0.13)
Median (IQR)	0.06 (-0.01, 0.13)	0.02 (-0.03, 0.08)	0.03 (-0.02, 0.10)
Missing	0	2	2
**PROMIS Emotional distress – Anxiety sub score**			
N	237	249	486
Mean (SD)	-2.28 (7.81)	-1.32 (7.59)	-1.79 (7.70)
Median (IQR)	-1.60 (-7.20, 1.80)	-0.60 (-6.0, 2.10)	-1.35 (-6.20, 1.90)
Missing	0	1	1
**PROMIS Emotional distress – Depression sub score**			
N	237	248	485
Mean (SD)	-2.05 (7.44)	-0.89 (7.48)	-1.46 (7.47)
Median (IQR)	0.00 (-5.40, 0.00)	0.00 (-5.05, 2.15)	0.00 (-5.30, 1.70)
Missing	0	2	2
**PROMIS Fatigue sub score**			
N	237	248	485
Mean (SD)	-4.34 (8.10)	-1.92 (7.59)	-3.10 (7.93)
Median (IQR)	-4.0 (-9.10, 0.00)	-0.15 (-6.55, 2.10)	-2.10 (-7.60, 0.90)
Missing	0	2	2
**PROMIS Sleep disturbance sub score**			
N	237	248	485
Mean (SD)	-2.29 (6.60)	-1.55 (6.90)	-1.91 (6.76)
Median (IQR)	-2.10 (-6.30, 1.50)	-1.70 (-5.45, 2.25)	-1.90 (-5.90, 1.80)
Missing	0	2	2
**PROMIS Pain interference sub score**			
N	237	248	485
Mean (SD)	-2.34 (7.30)	-0.68 (8.28)	-1.49 (7.85)
Median (IQR)	-0.80 (-5.90, 1.40)	0.00 (-4.55, 1.80)	0.00 (-5.50, 1.60)
Missing	0	2	2
**PROMIS Pain intensity**			
N	237	247	484
Mean (SD)	-0.46 (1.88)	-0.17 (2.30)	-0.31 (2.11)
Median (IQR)	0.00 (-2.0, 0.00)	0.00 (-1.0, 1.0)	0.00 (-1.0, 1.0)
Missing	0	3	3
**PROMIS Physical function sub score**			
N	237	249	486
Mean (SD)	1.29 (4.80)	0.70 (5.14)	0.99 (4.98)
Median (IQR)	1.00 (-0.90, 3.70)	0.10 (-1.50, 2.90)	0.60 (-1.30, 3.40)
Missing	0	1	1
**PROMIS Social roles and activities sub score**			
N	237	248	485
Mean (SD)	2.26 (6.44)	2.15 (7.19)	2.20 (6.83)
Median (IQR)	1.90 (-1.70, 5.90)	1.75 (-1.55, 6.0)	1.80 (-1.60, 6.0)
Missing	0	2	2
**PROMIS Cognitive function abilities sub score**			
N	237	248	485
Mean (SD)	0.777 (7.071)	0.654 (6.712)	0.714 (6.883)
Median (IQR)	0.00 (-3.20, 4.90)	0.00 (-3.40, 4.90)	0.00 (-3.40, 4.90)
Missing	0	2	2

Values are mean (SD), median (IQR) as appropriate. HRQoL, health-related quality of life; PROPr, PROMIS preference score; PROMIS, patient-reported outcomes measurement information system. Missing data are caused by partially missing questionnaire items.

Table S13: Secondary outcomes at 3 months follow-up

	Intervention	Usual care	TOTAL	Unadjusted estimate (95% CI); p-value	Adjusted estimate (95% CI)*; p-value
**EQ5D-5L Index Score**					
N	237	245	482	0.020 (-0.028, 0.068); 0.407	0.017 (-0.013, 0.048); 0.256
Mean (SD)	0.597 (0.273)	0.577 (0.254)	0.586 (0.263)		
Median (IQR)	0.666 (0.533, 0.755)	0.648 (0.473, 0.735)	0.654 (0.497, 0.736)		
Missing**	61	37	98		
Missing***	0	5	5		
**EQ5D-5L VAS**					
N	236	245	481	4.571 (0.797, 8.346); 0.018	3.373 (0.232, 6.515); 0.036
Mean (SD)	62.3 (19.1)	57.6 (21.6)	59.9 (20.5)		
Median (IQR)	61.5 (50.0, 77.5)	60.0 (41.0, 75.0)	60.0 (46.0, 76.0)		
Missing**	61	37	98		
Missing***	1	5	6		
**PTSD Symptom Severity (IES-r)**					
N	192	188	380	3.380 (-0.564, 7.325); 0.092	2.613 (0.083, 5.143); 0.043
Mean (SD)	24.6 (18.0)	28.1 (20.1)	26.4 (19.1)		
Median (IQR)	21.0 (11.0, 35.5)	26.0 (11.0, 42.5)	23.5 (11.0, 39.5)		
Missing**	61	37	98		
Missing***	45	62	107		
**HADS Anxiety Score**					
N	212	214	426	0.593 (-0.384, 1.571); 0.231	0.289 (-0.366, 0.944); 0.384
Mean (SD)	8.0 (4.8)	8.6 (4.8)	8.3 (4.8)		
Median (IQR)	8.0 (4.0, 11.0)	8.0 (5.0, 12.0)	8.0 (5.0, 12.0)		
Missing**	61	37	98		
Missing***	25	36	61		
**HADS Depression Score**					
N	206	216	422	0.755 (-0.136, 1.646); 0.096	0.458 (-0.135, 1.050); 0.128
Mean (SD)	7.7 (4.5)	8.4 (4.8)	8.1 (4.6)		
Median (IQR)	7.0 (4.0, 10.0)	8.0 (5.0, 11.0)	8.0 (4.0, 11.0)		
Missing**	61	37	98		
Missing***	31	34	65		
**PROMIS Dyspnoea Severity Short Form**					
N	219	224	443	0.963 (0.746, 2.673); 0.267	0.926 (-0.089, 1.940); 0.073
Mean (SD)	53.5 (8.6)	54.4 (9.5)	54.0 (9.1)		

	Intervention	Usual care	TOTAL	Unadjusted estimate (95% CI); p-value	Adjusted estimate (95% CI)*; p-value
Median (IQR)	53.1 (48.5, 58.9)	54.8 (48.5, 60.5)	53.9 (48.5, 60.1)		
Missing**	61	37	98		
Missing***	18	26	44		
**Cognitive Function (PROMIS Neuro-QoL)**					
N	221	223	444	-0.150 (-1.907, 1.606); 0.866	-0.251 (-1.446, 0.944); 0.678
Mean (SD)	40.5 (9.3)	40.7 (9.4)	40.6 (9.3)		
Median (IQR)	41.2 (33.5, 45.6)	40.3 (32.9, 46.7)	41.0 (33.4, 46.1)		
Missing**	61	37	98		
Missing***	16	27	43		
**Physical activity IPAQ-SF (MET mins.week^{-1})**					
N	221	222	443	1.24 (0.88, 1.75); 0.214	1.66[‡] (1.14, 2.41); 0.008
<600 (Low)	59 (26.7%)	76 (34.2%)	135 (30.5%)		
≥ 600 to 3000 (Moderate)	77 (34.8%)	66 (29.7%)	143 (32.3%)		
≥ 3000 (High)	85 (38.5%)	80 (36.0%)	165 (37.3%)		
Missing**	61	37	98		
Missing***	16	28	44		
**Overall health: Compared to three months ago**					
Much better now	39 (16.5%)	20 (8.1%)	59 (12.2%)	0.311 (0.127, 0.494); 0.001	0.296 (0.130, 0.461); 0.001
Somewhat better now	81 (34.2%)	60 (24.2%)	141 (29.1%)		
About the same	72 (30.4%)	108 (43.6%)	180 (37.1%)		
Somewhat worse now	19 (8.0%)	27 (10.9%)	46 (9.5%)		
Much worse now	5 (2.1%)	5 (2.0%)	10 (2.1%)		
Missing**	61	37	98		
Missing***	21 (8.9%)	30 (11.3)	51 (10.1%)		

Values are mean (SD), median (IQR), number (%) as appropriate. VAS, visual analogue scale; PTSD, post-traumatic stress; IES-R, index of event scale – revised; HADS, hospital anxiety and depression score; PROMIS, patient-reported outcomes measurement information system. IPAQ-SF, international physical activity questionnaire – short form; MET, metabolic equivalent (1 MET is equivalent to resting energy expenditure, i.e., 3.5 ml.kg.min^{-1}). *Based on a partially nested heteroscedastic model adjusted for baseline overall health and stratification variables (age, level of hospital, and level of mental health disorder). The therapist effect was included as a random effect to account for partial clustering; ** missing due to lost to follow-up and withdrawals; ***participants who provided their records, but the form was incomplete (fully or partially) and therefore their record couldn't be included in the analysis. [‡]Odds ratio; mixed effect ordered logistic regression model.

Table S14: Secondary outcomes at 6 months follow-up

	Intervention	Usual care	TOTAL	Unadjusted estimate (95% CI); p-value	Adjusted estimate (95% CI)*; p-value
**EQ5D-5L Index Score**					
N	225	233	458	0.014 (-0.034, 0.063); 0.558	0.017 (-0.019, 0.052); 0.351
Mean (SD)	0.604 (0.27)	0.589 (0.26)	0.596 (0.26)		
Median (IQR)	0.678 (0.533, 0.768)	0.652 (0.516, 0.743)	0.657 (0.523, 0.767)		
Missing**	1	6	7		
**EQ5D-5L VAS**					
N	225	233	458	0.651 (-3.627, 4.929); 0.764	-0.490 (-4.106, 3.126); 0.788
Mean (SD)	59.2 (21.0)	58.2 (21.8)	58.7 (21.4)		
Median (IQR)	60.0 (43.0, 75.0)	62.0 (41.0, 75.0)	60.0 (41.0, 75.0)		
Missing**	1	6	7		
**PTSD Symptom Severity (IES-r)**					
N	188	184	372	3.634 (-0.324, 7.591); 0.072	3.036 (0.304, 5.769); 0.030
Mean (SD)	22.7 (18.7)	26.4 (19.4)	24.6 (19.1)		
Median (IQR)	18.0 (9.0, 31.0)	23.5 (10.0, 40.0)	21.0 (9.0, 36.5)		
Missing**	38	55	93		
**PTSD Symptom Severity (IES-6)**					
N	225	236	461		
Score ≥11	60 (26.7%)	95 (40.3%)	155 (33.6%)		
**HADS Anxiety Score**					
N	209	214	423	0.871 (-0.099, 1.842); 0.078	0.559 (-0.111, 1.229); 0.101
Mean (SD)	7.9 (5.2)	8.8 (4.8)	8.3 (5.0)		
Median (IQR)	8.0 (4.0, 12.0)	9.0 (5.0, 12.0)	8.0 (4.0, 12.0)		
Missing**	17	25	42		
Score ≥11	77 (35.3%)	93 (41.2%)	170 (38.3%)		
**HADS Depression Score**					
N	205	219	424	1.049 (0.117, 1.981); 0.028	0.625 (-0.047, 1.298); 0.068
Mean (SD)	7.6 (5.0)	8.6 (4.7)	8.1 (4.8)		
Median (IQR)	7.0 (4.0, 11.0)	8.0 (5.0, 12.0)	8.0 (4.0, 11.0)		
Missing**	21	20	41		
Score ≥11	65 (29.8%)	74 (32.7%)	139 (31.3%)		
**PROMIS Dyspnoea Severity Short Form**					
N	217	225	442	0.563 (-1.360, 2.485); 0.563	0.403 (-0.731, 1.538); 0.482
Mean (SD)	53.3 (9.5)	54.0 (9.5)	53.6 (9.5)		

	Intervention	Usual care	TOTAL	Unadjusted estimate (95% CI); p-value	Adjusted estimate (95% CI)*; p-value
Median (IQR)	53.7 (46.6, 59.8)	54.1 (48.2, 59.8)	54.0 (47.8, 59.8)		
Missing**	9	14	23		
**Cognitive Function (PROMIS neuroQOL)**					
N	218	225	443	-0.406 (-2.360, 1.548); 0.681	-0.792 (-2.161, 0.576); 0.254
Mean (SD)	41.3 (9.6)	41.6 (9.9)	40.4 (9.8)		
Median (IQR)	41.2 (34.7, 46.7)	41.0 (34.0, 47.6)	41.1 (34.6, 47.3)		
Missing**	8	14	22		
**Physical activity IPAQ-SF (MET mins.week^{-1})**					
N	218	224	442	1.151‡ (0.816, 1.622); 0.424	1.376‡ (0.956, 1.980); 0.086
<600 (Low)	77 (35.3%)	92 (41.1%)	169 (38.2%)		
≥ 600 to 3000 (Moderate)	69 (31.7%)	59 (26.3%)	128 (29.0%)		
≥ 3000 (High)	72 (33.0%)	73 (32.6%)	145 (32.8%)		
Missing**	2	21	23		
**Overall Health: Compared to three months ago**					
Much better now	32 (14.7%)	15 (6.7%)	47 (10.6%)	0.332 (0.144, 0.520); 0.001	0.321 (0.139, 0.503); 0.001
Somewhat better now	65 (29.8%)	50 (22.3%)	115 (26.0%)		
About the same	80 (36.7%)	102 (45.5%)	182 (41.2%)		
Somewhat worse now	29 (13.3%)	45 (20.1%)	74 (16.7%)		
Much worse now	9 (4.1%)	12 (5.4%)	21 (4.8%)		
Missing**	3 (1.4%)	0 (0%)	3 (0.7%)		

Values are mean (SD), median (IQR), number (%) as appropriate. VAS, visual analogue scale; PTSD, post-traumatic stress; IES-R, index of event scale – revised; HADS, hospital anxiety and depression score; PROMIS, patient-reported outcomes measurement information system; IPAQ-SF, international physical activity questionnaire – short form; MET, metabolic equivalent (1 MET is equivalent to resting energy expenditure, i.e., 3.5 ml.kg.min^{-1}). *Based on a partially nested heteroscedastic model adjusted for baseline overall health and stratification variables (age, level of hospital, and level of mental health disorder). The therapist effect was included as a random effect to account for partial clustering. ** participants who provided their records, but the form was incomplete (fully or partially) and therefore their record couldn't be included in the analysis. ‡Odds ratio; mixed effect ordered logistic regression model.

Table S15: Secondary outcomes at 12 months follow-up

	Intervention	Usual care	TOTAL	Unadjusted estimate (95% CI); p-value	Adjusted estimate (95% CI)*; p-value
**EQ5D-5L Index Score**					
N	215	223	438	0.031 (-0.021, 0.083); 0.236	0.032 (-0.006, 0.069); 0.097
Mean (SD)	0.615 (0.27)	0.583 (0.25)	0.599 (0.26)		
Median (IQR)	0.659 (0.531, 0.768)	0.642 (0.426, 0.740)	0.654 (0.479, 0.767)		
Missing**	1	3	4		
**EQ5D-5L VAS**					
N	215	223	438	5.357 (1.235, 9.479); 0.011	3.768 (0.316, 7.219); 0.033
Mean (SD)	63.8 (21.0)	58.4 (22.9)	61.0 (21.9)		
Median (IQR)	68.0 (50.0, 80.0)	60.0 (40.0, 78.0)	65.0 (43.0, 80.0)		
Missing**	1	3	4		
**PTSD Symptom Severity (IES-r)**					
N	203	208	411	5.371 (1.640, 9.101); 0.005	4.366 (1.664, 7.069); 0.002
Mean (SD)	21.4 (17.6)	26.7 (20.6)	24.1 (19.3)		
Median (IQR)	17.0 (7.0, 32.0)	24.0 (8.0, 44.0)	21.0 (7.0, 36.0)		
Missing**	13	18	31		
**PTSD Symptom Severity (IES-6)**					
N	215	225	440		
Score ≥11	47 (21.7%)	82 (36.4%)	129 (29.3%)		
**HADS Anxiety Score**					
N	208	215	423	0.899 (-0.116, 1.913); 0.082	0.492 (-0.229, 1.213); 0.179
Mean (SD)	7.2 (5.3)	8.2 (5.0)	7.7 (5.2)		
Median (IQR)	7.0 (3.0, 11.0)	8.0 (5.0, 12.0)	7.0 (4.0, 11.0)		
Missing**	8	11	19		
Score ≥11	59 (27.7%)	73 (33.5%)	132 (30.6%)		
**HADS Depression Score**					
N	206	211	417	1.35 (0.357, 2.343); 0.008	0.952 (0.229, 1.675); 0.010
Mean (SD)	6.8 (4.8)	8.3 (4.7)	7.6 (4.8)		
Median (IQR)	6.0 (3.0, 10.0)	8.0 (5.0, 11.0)	8.0 (4.0, 11.0)		
Missing**	10	15	25		
Score ≥11	56 (26.3%)	78 (35.8%)	134 (31.1%)134		
**PROMIS Dyspnoea Severity Short Form**					
N	210	215	425	0.952 (-1.069, 2.972); 0.352	0.803 (-0.559, 2.164); 0.245
Mean (SD)	52.5 (9.7)	53.5 (10.0)	53.0 (10.0)		

	Intervention	Usual care	TOTAL	Unadjusted estimate (95% CI); p-value	Adjusted estimate (95% CI)*; p-value
Median (IQR)	52.3 (46.6, 58.7)	53.8 (47.4, 60.2)	52.6 (47.0, 59.7)		
Missing**	6	11	17		
**Cognitive Function (PROMIS neuroQOL)**					
N	210	217	427	0.952 (-1.035, 2.939); 0.344	0.865 (-0.449, 2.179); 0.194
Mean (SD)	42.5 (9.6)	41.4 (10.0)	42.0 (9.8)		
Median (IQR)	42.3 (36.4, 48.5)	41.2 (33.5, 48.3)	41.9 (34.4, 48.4)		
Missing**	6	9	15		
**Physical activity IPAQ-SF (MET mins.week^{-1})**					
N	211	217	428	1.06‡ (0.71, 1.59); 0.767	1.19‡ (0.78, 1.82); 0.415
<600 (Low)	61 (28.9%)	69 (31.8%)	130 (30.4%)		
≥ 600 to 3000 (Moderate)	73 (34.6%)	75 (34.6%)	148 (34.6%)		
≥ 3000 (High)	77 (36.5%)	73 (33.6%)	150 (35.1%)		
Missing**	5	9	14		
**Overall Health: Compared to three months ago**					
Much better now	44 (20.9%)	25 (11.6%)	69 (16.2%)	0.359 (0.131, 0.587); 0.002	0.362 (0.144, 0.581); 0.001
Somewhat better now	54 (25.6%)	41 (19.0%)	95 (22.3%)		
About the same	70 (33.2%)	87 (40.3%)	157 (36.8%)		
Somewhat worse now	36 (17.1%)	49 (22.7%)	85 (19.9%)		
Much worse now	6 (2.8%)	14 (6.5%)	20 (4.7%)		
Missing**	1 (0.5%)	0 (0%)	1 (0.2%)		

Values are mean (SD), median (IQR), number (%) as appropriate. VAS, visual analogue scale; PTSD, post-traumatic stress; IES-R, index of event scale – revised; HADS, hospital anxiety and depression score; PROMIS, patient-reported outcomes measurement information system; IPAQ-SF, international physical activity questionnaire – short form; MET, metabolic equivalent (1 MET is equivalent to resting energy expenditure, i.e., 3.5 ml.kg.min^{-1}). *Based on a partially nested heteroscedastic model adjusted for baseline overall health and stratification variables (age, level of hospital, and level of mental health disorder). The therapist effect was included as a random effect to account for partial clustering. ** participants who provided their records, but the form was incomplete (fully or partially) and therefore their record couldn't be included in the analysis. ‡Odds ratio; mixed effect ordered logistic regression model.

Table S16: Sub-group analyses of the 3-month primary outcome (PROPr)

Subgroups		Intervention N; mean (SD)	Usual care N; mean (SD)	Interaction effect (95% CI); p-value*
**Age, years**				
	<65	175; 0.248 (0.179)	184; 0.230 (0.180)	0.051 (-0.023, 0.125); 0.175
	≥65	62; 0.316 (0.187)	64; 0.246 (0.186)	
**Level of hospital care**				
	Critical care	83; 0.270 (0.198)	85; 0.206 (0.166)	-0.050 (-0.118, 0.019); 0.155
	Ward	154; 0.263 (0.176)	163; 0.248 (0.188)	
**HADS Depression**				
	<11	156; 0.326 (0.173)	147; 0.298 (0.175)	-0.019 (-0.080, 0.042); 0.541
	≥11	81; 0.149 (0.143)	101; 0.141 (0.148)	
**HADS Anxiety**				
	<11	144; 0.333 (0.176)	140; 0.294 (0.186)	-0.034 (-0.094, 0.026); 0.262
	≥11	93; 0.160 (0.140)	108; 0.155 (0.141)	
**PTSD (IES-6)**				
	Yes (≥11)	79; 0.145 (0.134)	114; 0.147 (0.149)	-0.020 (-0.080, 0.040); 0.508
	No (<11)	158; 0.326 (0.175)	134; 0.308 (0.174)	
**Ethnicity**				
	Non-White	25; 0.246 (0.152)	29; 0.261 (0.154)	-0.058 (-0.162, 0.046); 0.272
	White	212; 0.268 (0.187)	219; 0.230 (0.185)	
**Wave of pandemic**				
	1st wave (Before August 2020)	40; 0.255 (0.172)	36; 0.233 (0.178)	
	2nd wave (September 2020 to December 2020)	30; 0.282 (0.199)	34; 0.247 (0.176)	0.019 (-0.103, 0.142); 0.757
	3rd wave (January 2021 to May 2021)	93; 0.258 (0.188)	88; 0.234 (0.191)	0.001 (-0.099, 0.101); 0.980
	4th wave (June 2021 to July 2022)	74; 0.273 (0.179)	90; 0.229 (0.178)	0.016 (-0.086, 0.118); 0.751
**Method of recruitment**				
	NHS digital mailouts	129; 0.278 (0.184)	135; 0.239 (0.181)	-0.015 (-0.082, 0.053); 0.669
	Others	108; 0.250 (0.182)	113; 0.227 (0.182)	

Values are mean (SD). PTSD, post-traumatic stress; IES-R, index of event scale – revised; HADS, hospital anxiety and depression score; *Based on a partially nested heteroscedastic model adjusted for baseline overall health and stratification variables (age, level of hospital, and level of mental health disorder). The therapist effect was included as a random effect to account for partial clustering. A score ≥11 on any of the PTSD-IES-6, HADS anxiety, or HADS depression scales was the threshold for case level mental health disorder.

Table S17: Unadjusted and adjusted estimates of treatment effect at 3-month time point adjusting for patient level covariates such as gender, BMI, and ethnicity

PROMIS score at 3 months	Unadjusted estimate (95% CI); p-value	Adjusted estimate* (95% CI); p-value
Baseline score	-	0.723 (0.638, 0.809); <0.001
Intervention (REGAIN)	0.030 (-0.004, 0.064); 0.088	0.030 (0.007, 0.052); 0.010
Age	-	-0.008 (-0.034, 0.018); 0.549
Mental health	-	-0.034 (-0.062, -0.006); 0.017
Hospital level	-	0.025 (0.001, 0.049); 0.038
Gender	-	-0.026 (-0.049, -0.002); 0.032

PROMIS score at 3 months	Unadjusted estimate (95% CI); p-value	Adjusted estimate* (95% CI); p-value
Body mass index (BMI)	-	-0.0001 (-0.001, 0.000); 0.566
Ethnicity	-	0.007 (-0.029, 0.042); 0.708

*Based on a partially nested heteroscedastic model adjusted for baseline overall health and stratification variables (age, level of hospital, and level of mental health disorder); The therapist effect was included as a random effect to account for partial clustering.

Table S18: Sensitivity analysis - treatment effectiveness estimate based on imputed datasets for the primary outcome to account for all missing data at 3-months (lost to follow-up, withdrawals and incomplete questionnaire)

	Intervention (N = 298)	Usual care (N = 287)	Unadjusted estimate (95% CI); p-value	Adjusted estimate (95% CI); p-value*
**PROMIS score at 3 months**			0.029** (-0.002, 0.060); 0.067	0.027 (0.005, 0.049); 0.016
N	298	287		
Mean (SD)	0.261 (0.185)	0.227 (0.181)		
Median (IQR)	0.239 (0.122, 0.371)	0.204 (0.087, 0.333)		

Values are mean (SD), median (IQR) as appropriate. *Based on a partially nested heteroscedastic model adjusted for baseline overall health and stratification variables (age, level of hospital, and level of mental health disorder); The therapist effect was included as a random effect to account for partial clustering; **Pooled results reported. PROMIS, patient-reported outcomes measurement information system

Table S19: Adverse events and serious adverse events (SAE) summarised by treatment group

	Intervention N=298	Usual care N=287	Total N=585
**AEs**			
Number of AEs reported	28 (9.4%)	16 (5.6%)	44 (7.5%)
**SAEs**			
Number of SAEs reported	14 (4.7%)	7 (2.4%)	21 (3.6%)
**Reason SAE deemed serious**			
Death	0 (0%)	0 (0%)	0 (0%)
Life-threatening	0 (0%)	0 (0%)	1 (0.2%)
Hospitalisation or prolongation of hospitalisation	12 (4.0%)*	7 (2.4%)	19 (3.2%)
Persistent or significant disability or incapacity	2 (0.7%)	0 (0%)	2 (0.3%)
Congenital anomaly/birth defect	0 (0%)	0 (0%)	0 (0%)
Other	1 (0.3%)*	0 (0%)	1 (0.2%)

Values as number (% of total randomised). AE, adverse events; SAE, serious adverse events. *1 participant coded under 2 categories for ‘reason SAE deemed serious’

Table S20: Assessment of AEs summarised by treatment group

Assessment of AE's	Intervention N=298	Usual care N=287	TOTAL N=585
**AE related to trial intervention**			
Definitely	2 (0.7%)	0 (0%)	2 (0.3%)
Probably	2 (0.7%)	0 (0%)	2 (0.3%)
Possibly	0 (0%)	0 (0%)	0 (0%)
Unlikely	0 (0%)	0 (0%)	0 (0%)
Unrelated	24 (8.1%)	16 (5.6%)	40 (6.8%)
Missing	0 (0%)	0 (0%)	0 (0%)
**Total**	28 (9.4%)	16 (5.6%)	44 (7.5%)

Values as number (% of total randomised). AE, adverse events; SAE, serious adverse events.

Table S21: Assessment of SAEs summarised by treatment group

Assessment of SAE's	Intervention N=298	Usual care N=287	TOTAL N=585
**SAE related to trial intervention**			
Definitely	0 (0%)	0 (0%)	0 (0%)
Probably	0 (0%)	0 (0%)	0 (0%)
Possibly	1 (0.3%)	0 (0%)	1 (0.2%)
Unlikely	0 (0%)	0 (0%)	0 (0%)
Unrelated	13 (4.4%)	7 (2.4%)	20 (3.4%)
Missing	0 (0%)	0 (0%)	0 (0%)
**Total**	14 (4.7%)	7 (2.4%)	21 (3.6%)

Values as number (% of total randomised). AE, adverse events; SAE, serious adverse events.

Table S22: Common Terminology Criteria for Adverse Events (CTCAE) for SAEs

System organ class	Grade 1	Grade 2	Grade 3	Grade 4	Grade 5	Unknown	Total*
Blood and lymphatic system disorders	0	0	0	0	0	0	0 (0%)
Cardiac disorders	0	1	0	1	0	0	2 (9.5%)
Eye disorders	0	1	0	0	0	0	1 (4.8%)
Gastrointestinal disorders	0	0	1	0	0	0	1 (4.8%)
Infections and manifestations	0	0	1	0	0	0	1 (4.8%)
Investigations	0	0	3	0	0	0	3 (14.3%)
Surgical and medical procedures	0	0	0	0	0	0	0 (0%)
Musculoskeletal and connective disorders	0	0	1	0	0	0	1 (4.8%)
Renal and urinary disorders	0	1	1	0	0	0	2 (9.5%)
Respiratory, thoracic, and mediastinal	0	2	6	0	0	0	8 (38.1%)
Vascular disorders	0	0	1	1	0	0	2 (9.5%)
Injury and poisoning	0	0	0	0	0	0	0 (0%)
Unknown	0	0	0	0	0	0	0 (0%)
**Total***	0 (0%)	5 (23.8%)	14 (66.7%)	2 (9.5%)	0 (0%)	0 (0%)	21 (100%)

Values as number (*% of total SAEs). AE, adverse events; SAE, serious adverse events.

CONSORT checklist

		Reporting Item	Page Number
**Title and Abstract**			
Title	#1a	Identification as a randomized trial in the title.	1
Abstract	#1b	Structured summary of trial design, methods, results, and conclusions	2
**Introduction**			
Background and objectives	#2a	Scientific background and explanation of rationale	4
Background and objectives	#2b	Specific objectives or hypothesis	4
**Methods**			
Trial design	#3a	Description of trial design (such as parallel, factorial) including allocation ratio.	5
Trial design	#3b	Important changes to methods after trial commencement (such as eligibility criteria), with reasons	n/a
Participants	#4a	Eligibility criteria for participants	6
Participants	#4b	Settings and locations where the data were collected	5,6
Interventions	#5	The experimental and control interventions for each group with sufficient details to allow replication, including how and when they were actually administered	6,7
Outcomes	#6a	Completely defined prespecified primary and secondary outcome measures, including how and when they were assessed	7,8
Sample size	#7a	How sample size was determined.	8,9
Sample size	#7b	When applicable, explanation of any interim analyses and stopping guidelines	n/a

Randomization - Sequence generation	#8a	Method used to generate the random allocation sequence.	
5			
Randomization - Sequence generation	#8b	Type of randomization; details of any restriction (such as blocking and block size)	
5			
Randomization - Allocation concealment mechanism	#9	Mechanism used to implement the random allocation sequence (such as sequentially numbered containers), describing any steps taken to conceal the sequence until interventions were assigned	5
Randomization - Implementation	#10	Who generated the allocation sequence, who enrolled participants, and who assigned participants to interventions	5
Blinding	#11a	If done, who was blinded after assignment to interventions (for example, participants, care providers, those assessing outcomes) and how.	5,6
Blinding	#11b	If relevant, description of the similarity of interventions	n/a
Statistical methods	#12a	Statistical methods used to compare groups for primary and secondary outcomes	8,9
Statistical methods	#12b	Methods for additional analyses, such as subgroup analyses and adjusted analyses	8,9
Outcomes	#6b	Any changes to trial outcomes after the trial commenced, with reasons	n/a
**Results**			
Participant flow diagram (strongly recommended)	#13a	For each group, the numbers of participants who were randomly assigned, received intended treatment, and were analysed for the primary outcome	Fig 1
Participant flow	#13b	For each group, losses and exclusions after randomization, together with reason	Fig 1
Recruitment	#14a	Dates defining the periods of recruitment and follow-up	10

Recruitment	#14b	Why the trial ended or was stopped	10
Baseline data	#15	A table showing baseline demographic and clinical characteristics for each group	Tables 1,2
Numbers analysed	#16	For each group, number of participants (denominator) included in each analysis and whether the analysis was by original assigned groups	10
Outcomes and estimation	#17a	For each primary and secondary outcome, results for each group, and the estimated effect size and its precision (such as 95% confidence interval)	10
Outcomes and estimation	#17b	For binary outcomes, presentation of both absolute and relative effect sizes is recommended	10
Ancillary analyses	#18	Results of any other analyses performed, including subgroup analyses and adjusted analyses, distinguishing pre-specified from exploratory	11
Harms	#19	All important harms or unintended effects in each group (For specific guidance see CONSORT for harms)	11
**Discussion**			
Limitations	#20	Trial limitations, addressing sources of potential bias, imprecision, and, if relevant, multiplicity of analyses	15
Interpretation	#22	Interpretation consistent with results, balancing benefits and harms, and considering other relevant evidence	12-14
Registration	#23	Registration number and name of trial registry	9
Generalisability	#21	Generalisability (external validity, applicability) of the trial findings	15
**Other information**			
Interpretation	#22	Interpretation consistent with results, balancing benefits and harms, and considering other relevant evidence	12-14
Registration	#23	Registration number and name of trial registry	9

Protocol	#24	Where the full trial protocol can be accessed, if available	Supplement 20
Funding	#25	Sources of funding and other support (such as supply of drugs), role of funders	9

TIDieR checklist

TIDieR Template for Intervention Description and Replication

The TIDieR (Template for Intervention Description and Replication) Checklist*:

Information to include when describing an intervention and the location of the information

Item number	Item	Where located ** Primary paper (page or appendix number)	Other † (details)
	**BRIEF NAME**		
1.	Provide the name or a phrase that describes the intervention.	____p.5______	Int. devpt. paper
	**WHY**		
2.	Describe any rationale, theory, or goal of the elements essential to the intervention.	__p.8-9______	Int. devpt. paper
	**WHAT**		
3.	Materials: Describe any physical or informational materials used in the intervention, including those provided to participants or used in intervention delivery or in training of intervention providers. Provide information on where the materials can be accessed (e.g. online appendix, URL).	__p.8-9______	Int. devpt. paper
4.	Procedures: Describe each of the procedures, activities, and/or processes used in the intervention, including any enabling or support activities.	__p.8-9______	Int. devpt. paper_
	**WHO PROVIDED**		
5.	For each category of intervention provider (e.g. psychologist, nursing assistant), describe their expertise, background and any specific training given.	__p.8-9______	Int. devpt. paper_
	**HOW**		
6.	Describe the modes of delivery (e.g. face-to-face or by some other mechanism, such as internet or telephone) of the intervention and whether it was provided individually or in a group.	__p.8-9______	Int. devpt. paper_
	**WHERE**		
7.	Describe the type(s) of location(s) where the intervention occurred, including any necessary infrastructure or relevant features.	__p.8-9_______	Int. devpt. paper_
	**WHEN and HOW MUCH**		
8.	Describe the number of times the intervention was delivered and over what period of time including the number of sessions, their schedule, and their duration, intensity or dose.	___p.8-9______	Int. devpt. paper_
	**TAILORING**		
9.	If the intervention was planned to be personalised, titrated or adapted, then describe what, why, when, and how.	___p.8-9_____	Int. devpt. paper_
	**MODIFICATIONS**		
10.‡	If the intervention was modified during the course of the study, describe the changes (what, why, when, and how).	___p.22_____	______________
	**HOW WELL**		
11.	Planned: If intervention adherence or fidelity was assessed, describe how and by whom, and if any strategies were used to maintain or improve fidelity, describe them.	___p.11________	______________
12.‡	Actual: If intervention adherence or fidelity was assessed, describe the extent to which the intervention was delivered as planned.	___p.21,22______	______________

TIDieR checklist

** **Authors** - use N/A if an item is not applicable for the intervention being described. **Reviewers** – use '?' if information about the element is not reported/not sufficiently reported.

† If the information is not provided in the primary paper, give details of where this information is available. This may include locations such as a published protocol or other published papers (provide citation details) or a website (provide the URL).

‡ If completing the TIDieR checklist for a protocol, these items are not relevant to the protocol and cannot be described until the study is complete.

* We strongly recommend using this checklist in conjunction with the TIDieR guide (see *BMJ* 2014;348:g1687) which contains an explanation and elaboration for each item.

* The focus of TIDieR is on reporting details of the intervention elements (and where relevant, comparison elements) of a study. Other elements and methodological features of studies are covered by other reporting statements and checklists and have not been duplicated as part of the TIDieR checklist. When a **randomised trial** is being reported, the TIDieR checklist should be used in conjunction with the CONSORT statement (see www.consort-statement.org) as an extension of **Item 5 of the CONSORT 2010 Statement.** When a **clinical trial protocol** is being reported, the TIDieR checklist should be used in conjunction with the SPIRIT statement as an extension of **Item 11 of the SPIRIT 2013 Statement** (see www.spirit-statement.org). For alternate study designs, TIDieR can be used in conjunction with the appropriate checklist for that study design (see www.equator-network.org).

TIDieR checklist

REHABILITATION
EXERCISE AND
PSYCHOLOGICAL
SUPPORT AFTER
COVID-19 INFECTION

PROTOCOL

Rehabilitation Exercise and psycholoGical support After covid-19 InfectioN' (REGAIN): a multi-centre randomised controlled trial

ISRCTN Number: 11466448

Sponsor: University Hospitals Coventry & Warwickshire (UHCW) NHS Trust

Funding Body: National Institute for Health Research – COVID-19 Recovery & Learning Cross Programme, NIHR number (NIHR132046).

Ethics Approval date: Cambridge South REC – 08th December 2020

Sponsor Ref: GM497120

Version Number: 450

Date: 28Jun2022

Stage: Approved

This protocol has regard for current HRA guidance and content

Protocol Amendments:

**Amendment No.**		**Date of Amendment**		**Date of Approval**
Pre REC approval		16th October 2020		N/A
Pre REC approval		29th October 2020		06th November 2020
Amendment 1		06th November 2020		08th December 2020
Amendment 2		15th December 2020		17th December 2020
Amendment 3		19th February 2021		16th March 2021
Amendment 4		1st June 2021		25th June 2021
Amendment 5		14th Sep 2021		21st Oct 2021; Not in use following withdrawn CAG application
Amendment 6		18th Jan 2022		25th February 2022
Non substantial amendment 16		28Jun2022		14th July 2022

CONTACT NAMES AND NUMBERS

Role	Name, address, telephone
**Sponsor:**	UHCW NHS Trust<br>R & D Department<br>Tel: 02476 966195<br>Email: R&DSponsorship@uhcw.nhs.uk
**Chief Investigator:**	Dr Gordon McGregor<br>Department of Cardio-pulmonary rehabilitation<br>University Hospitals Coventry & Warwickshire<br>Coventry CV2 2DX<br>Email: gordon.mcgregor@uhcw.nhs.uk
**Senior Project Manager:**	Jessica Smith<br>Warwick Clinical Trials Unit<br>Tel: 024 76150088<br>Email: Jessica.Smith.1@warwick.ac.uk
**Trial Manager:**	Sharisse Alleyne<br>Warwick Clinical Trials Unit<br>Tel: 024 76151719<br>Email: regain@warwick.ac.uk

**Co-investigators:**	Professor Martin Underwood (Co-applicant)<br>Professor of Primary Care Research<br>Warwick Clinical Trials Unit<br>Email: m.underwood@warwick.ac.uk<br><br>Professor Julie Bruce (Co-applicant)<br>Professor of Clinical Trials<br>Warwick Clinical Trials Unit<br>Email: Julie.Bruce@warwick.ac.uk<br><br>Professor Kate Seers (Co-applicant)<br>Professor of Health Research<br>Warwick Research in Nursing, Health Sciences Division<br>Email: kate.seers@warwick.ac.uk<br><br>Dr Harbinder Sandhu (Co-applicant)<br>Associate Professor of Health Psychology<br>Warwick Clinical Trials Unit<br>Email: harbinder.K.Sandhu@warwick.ac.uk<br><br>Dr Bart Sheehan (Co-applicant)<br>Consultant Psychiatrist<br>Coventry and Warwickshire Partnership NHS Trust<br>Email: bart.sheehan@ouh.nhs.uk<br><br>Dr David McWilliams (Co-applicant)<br>Associate Clinical Professor Physiotherapy<br>University Hospitals Coventry and Warwickshire<br>Email: David.mcwilliams@uhcw.nhs.uk<br><br>Associate Professor Joyce Yeung (Co-applicant)<br>Consultant Critical Care Physician<br>UHCW NHS Trust<br>Email: j.yeung.4@warwick.ac.uk<br><br>Dr Beatriz Lara (Co-applicant)<br>Consultant Respiratory Physician<br>UHCW NHS Trust<br>Email: beatriz.lara@uhcw.nhs.uk<br><br>Dr Christina Jones (Co-applicant)<br>Research Manager<br>ICUsteps Peer Support Group Charity<br>Email: christina.jones@icusteps.org<br><br>Professor Ranjit Lall (Co-applicant)<br>Professor of Clinical Trials and Biostatistics<br>Warwick Clinical Trials Unit

Role	Name, address, telephone
	Email: R.Lall@warwick.ac.uk<br><br>Professor James Mason (Co-applicant)<br>Professor of Health Economics<br>Warwick Medical School<br>Email: J.Mason@warwick.ac.uk<br><br>Rachel Potter<br>Senior Research Fellow<br>Warwick Clinical Trials Unit<br>Email: r.potter@warwick.ac.uk<br><br>Stuart Ennis<br>Research Fellow<br>Warwick Clinical Trials Unit<br>Email: stuart.ennis@warwick.ac.uk<br><br>Mrs Elaine Fairbrother (Co-applicant)<br>PPI<br>PPI based in England
**Statisticians:**	Professor Ranjit Lall (Co-applicant)<br>Professor of Clinical Trials and Biostatistics<br>Warwick Clinical Trials Unit<br>Email: R.Lall@warwick.ac.uk<br><br>Dr Chen Ji (Co-applicant)<br>Senior Research Fellow - Statistics<br>Warwick Clinical Trials Unit<br>Email: C.Ji.3@warwick.ac.uk<br><br>Mariam Ratna<br>Research Associate (Statistician)<br>Warwick Clinical Trials Unit<br>Email: Mariam.Ratna@warwick.ac.uk
**Health Economists:**	Professor James Mason (Co-applicant)<br>Professor of Health Economics<br>Warwick Medical School<br>Email: J.Mason@warwick.ac.uk<br><br>Dr Alastair Canaway<br>Health Economist<br>Warwick Medical School<br>Email: A.canaway@warwick.ac.uk

Role	Name, address, telephone
**Trial Steering Committee Membership**:	John Saxton – Chair<br>Professor of Clinical Exercise Physiology<br>University of Hull<br>Email: john.saxton@hull.ac.uk<br><br>Prof Reuben Ogollah – Independent Member<br>Associate Professor of Medical Statistics and Clinical Trials<br>Nottingham Clinical Trials Unit<br>Email: Reuben.Ogollah@nottingham.ac.uk<br><br>Dr Tamas Szakmany – Independent Member<br>Consultant in Intensive Care and Anaesthesia<br>Aneurin Bevan University Health Board<br>Email: szakmanyt1@cardiff.ac.uk<br><br>Dr Ben Creagh-Brown – Independent Member<br>Consultant Physician – Critical Care<br>Royal Surrey NHS Foundation Trust<br>Email: bencb@nhs.net<br><br>Professor Tamar Pincus – Independent Member<br>Professor of Health Psychology<br>Royal Holloway University of London<br>Email: t.pincus@rhul.ac.uk<br><br>Mr Matthew Cox – Independent Member<br>Respiratory Physiotherapist<br>Sheffield Teaching Hospitals NHS Foundation Trust<br>Email: matthew.cox3@nhs.net<br><br>Dr Paul Chazot – PPI Member<br>Associate Professor<br>Durham University<br>paul.chazot@durham.ac.uk

Role	Name, address, telephone
**Data Monitoring Committee Membership:**	Prof Dawn Teare - Chair<br>Professor of Biostatistics<br>PHSI Biostatistics Research Group<br>Tel: 0191 222 7045<br>Email: dawn.teare@newcastle.ac.uk<br><br>Professor Christopher Armitage – Independent Member<br>Manchester Centre for Health Psychology, University of Manchester<br>Tel: +44 (0)161 275 2556<br>E-mail: chris.armitage@manchester.ac.uk<br><br>Prof. Nicholas Hart – Independent Member<br>Professor of Respiratory & Critical Care Medicine<br>Guy's and St Thomas' NHS Foundation Trust<br>Email: Nicholas.Hart@gstt.nhs.uk
For general queries and supply of study materials please contact the coordinating centre:<br>Warwick Clinical Trials Unit (WCTU)<br>The University of Warwick<br>Gibbet Hill Road<br>Coventry<br>CV4 7AL<br>Tel: 02476 151719<br>Email: REGAIN@warwick.ac.uk	

TABLE OF CONTENTS PAGE

TABLE OF CONTENTS 7
STUDY SUMMARY 10
LIST OF ABBREVIATIONS/GLOSSARY 12
1. BACKGROUND 14
1.1 Epidemiology and burden of the condition 14
1.2 Existing knowledge 14
1.3 Hypothesis 15
1.4 Need for a study 16
1.5 Ethical considerations 16
1.6 CONSORT 18
2. STUDY DESIGN 18
2.1 Study summary and flow diagram 18
2.2 Aims and objectives 21
2.2.1 Primary objective 21
2.2.2 Secondary objective 21
2.2.3 Symptoms sub-study objective 21
2.2.4 Process Evaluation objective 21
2.3 Outcome measures 21
2.3.1 Efficacy 21
2.3.2 Symptoms sub-study 23
2.4 Eligibility criteria 24
2.4.1 Inclusion criteria 24
2.4.2 Exclusion criteria 24
2.5 Participant identification / Screening 24
2.6 Eligibility and informed consent 25
2.6.1 Consent for qualitative interviews 27
2.6.2 Consent for photographs and video clips 28
2.6.3 Consent for qualitative practitioner interviews 28
2.7 Site Staff Training 28
2.8 Randomisation 29
2.8.1 Randomisation 29
2.8.2 Post-randomisation withdrawals and exclusions 29
2.9 Study interventions 30
2.9.1 Study treatment(s) / intervention(s) 30
2.9.2 Best practice usual care (Control) intervention 30

2.9.3 REGAIN Intervention 30
2.9.4 Compliance 33
2.10 Concomitant illness and medication 34
2.10.1 Concomitant illness 34
2.10.2 Concomitant medication 34
2.11 Co-enrolment into other trials 34
2.12 End of study 34
3. METHODS AND ASSESSMENTS 35
3.1 Schedule of delivery of intervention and data collection 35
3.2 Long term follow-up assessments 36
3.3 Symptoms sub-study 36
3.4 Embedded process evaluation 36
4. ADVERSE EVENT MANAGEMENT 37
4.1 Definitions 37
4.1.1 Adverse Events (AE) 37
4.1.2 Serious Adverse Events (SAEs) 37
4.2 Recording Adverse Events and Reporting Serious Adverse Events 38
4.2.1 Recording and reporting period 38
4.2.2 Recording Adverse Events 38
4.2.3 Reporting Serious Adverse Events 38
4.2.3.1 SAEs that are exempt from reporting 39
4.2.4 Determination of causality and expectedness for SAEs 39
4.2.4.1 Expected Serious Adverse Events 39
4.2.5 Follow-up of reported SAEs 40
4.3 Responsibilities 40
4.4 Notification of deaths 41
4.5 Reporting urgent safety measures 42
5. DATA MANAGEMENT 42
5.1 Data collection and management 42
5.2 Database 43
5.3 Online video platform 43
5.4 One-to-one consultation platform 43
5.5 Data storage 44
5.6 Data access and quality assurance 44
5.7 Data Shared with Third Parties 44
5.8 Archiving 44
6. STATISTICAL ANALYSIS 45
6.1 Power and sample size 45

6.2 Statistical analysis of efficacy and harms 45
6.2.1 Statistics and data analysis 45
6.2.2 Planned recruitment rate 45
6.3 Subgroup analyses 46
6.4 Health economic evaluation 46
6.5 Qualitative data analysis 46
7. STUDY ORGANISATION AND OVERSIGHT 47
7.1 Sponsorship and governance arrangements 47
7.2 Ethical approval 48
7.3 Peer Review 48
7.4 Study Registration 48
7.5 Notification of serious breaches to GCP and/or study protocol 48
7.6 Indemnity 48
7.7 Study timetable and milestones 49
7.8 Administration 49
7.9 Trial Management Group (TMG) 49
7.10 Trial Steering Committee (TSC) 49
7.11 Data Monitoring Committee (DMC) 50
7.12 Essential Documentation 50
7.13 Financial Support 50
8. MONITORING, AUDIT AND INSPECTION 50
9. PATIENT AND PUBLIC INVOLVEMENT (PPI) 51
10. DISSEMINATION AND PUBLICATION 52
11. REFERENCES 53
12. APPENDICES 56
12.1 Appendix 1 - Logic model for the REGAIN psychological intervention. 56

LIST OF TABLES PAGE

Table 1 Study assessments 35
Table 2 SAE Causal relationship 39

LIST OF FIGURES PAGE

Figure 1 Study flow diagram 20

STUDY SUMMARY

Study Title	Rehabilitation Exercise and psycholoGical support After covid-19 InfectioN' (REGAIN): a multi-centre randomised controlled trial
Short study title	REGAIN
Clinical Phase	Phase III
Study Design	Multi-centre randomised controlled trial with embedded process evaluation and health economic evaluation
Study Participants	UK adults aged 18 years and older with ongoing COVID-19 sequelae more than three months after UK hospital discharge
Planned sample size	535 people randomly allocated to receive the REGAIN intervention or control; 1.03: 1 allocation
Treatment Duration	Eight weeks post randomisation
Follow-up Duration	12 months post randomisation
Planned Study Period	01 Nov 2020 to 31 August 2022
Objective	To run a multi-centre RCT testing the clinical and cost-effectiveness of an intensive, on-line, supervised, group, home-based rehabilitation programme to support long-term physical and mental health recovery (REGAIN) vs. best-practice usual care discharged from hospital (>3/12) after COVID-19 infection.
Outcomes<br><br>Primary<br><br>Secondary	Assessed at baseline pre-randomisation, three, six and 12 months post-randomisation.<br><br>Health-related quality of life (HRQoL): PROMIS® 29+2 Profile v2.1 (PROPr) measured at three months post-randomisation<br><br>1. HRQoL: PROMIS® 29+2 Profile v2.1 (PROPr) at six and 12 months post randomisation.<br><br>2. Dyspnoea: PROMIS dyspnoea severity short form v1.0<br><br>3. Cognitive Function: PROMIS Neuro-QoL Short Form v2.0

	4. Health Utility: Euroqol (EQ-5D-5L)<br><br>5. Physical activity. International Physical Activity Questionnaire short form (IPAQ-SF)<br><br>6. PTSD symptom severity: Impact of Event Scale - Revised (IES-R)<br><br>7. Depressive and Anxiety Symptoms: Hospital anxiety and depression scale (HADS)<br><br>8. Work Status: Time lost from work (paid/unpaid) and patient-borne health care costs.<br><br>9. Health and Social Care resource use: Participant self-report, NHS and GP records<br><br>10. General health – Participant self-reported assessment of overall health<br><br>11. Death – NHS and GP records	
Sub-studies	Objectives	Outcome Measures
Symptoms Sub Study	To explore the relationship between personal characteristics and in-hospital care, and subsequent ongoing COVID-19 symptoms and other health problems	Ongoing COVID-19 symptoms,<br>Ethnicity,<br>Age,<br>Gender,<br>Duration of hospital stay,<br>Need for high flow oxygen/continuous positive airways pressure/ventilation
Process evaluation Qualitative	To explore and contextualise participant and practitioner experience of the study and intervention delivery, barriers and enablers, to inform interpretation of quantitative data and facilitate wider implementation	Semi-structured interviews with participants and practitioners

LIST OF ABBREVIATIONS/GLOSSARY

Abbreviation	Explanation
AE	Adverse Event
CACE	Compliers Average Causal Effect
CI	Chief Investigator
CONSORT	*Con*solidated *S*tandards *o*f *R*eporting *T*rials
CRF	Case Report Form
CTU	Clinical Trials Unit
DMC	Data Monitoring Committee
GCP	Good Clinical Practice
HADS	Hospital Anxiety and Depression Scale
HRA	Health Research Authority
HRQoL	Health-Related Quality of Life
ICF	Informed Consent Form
IES-6	Impact of Event Scale – 6
IES-R	Impact of Event Scale – Revised
IPAQ-SF	International Physical Activity Questionnaire short form
IRAS	Integrated Research Application System
ISRCTN	International Standard Randomised Controlled Trial Number
MRC	Medical Research Council
NHS	NHS
NIHR	National Institute for Health Research
ORCHA	Organisation Review of Care and Health Apps (ORCHA)
PI	Principal Investigator
PIS	Participant Information Sheet
PPI	Patient & Public Involvement
PROMIS	PROMIS – add as this is primary outcome
QoL	Quality of Life
RCT	Randomised Controlled Trial
REC	Research Ethics Committee
R&D	Research and Development
SAE	Serious Adverse Event
SARS	Severe Acute Respiratory Syndrome
SOP	Standard Operating Procedure

TMG	Trial Management Group
TSC	Trial Steering Committee
UHCW	University Hospitals Coventry and Warwickshire NHS Trust
WCTU	Warwick Clinical Trials Unit

1. BACKGROUND

1.1 Epidemiology and burden of the condition

At least 80 thousand people in the UK have been discharged from hospital by the NHS after treatment for COVID-19. Many will return relatively quickly to good health and a normal life [1]. However, a substantial proportion of people will have ongoing health problems. These problems are multi-systemic [2] including motor, cognitive, neurological, musculoskeletal, respiratory and cardiovascular as well as depression, anxiety, and post-traumatic stress disorder (PTSD) [3]. In April 2020, the NHS predicted that 45% of people discharged from hospital would need some ongoing support from health and/or social care [4]. In June 2020, Public Health England confirmed that the virus, and its treatment, would have a lasting impact on the health of survivors [1]. The actual proportion with long-term health problems after the initial recovery phase remains unknown. However, the scale of the COVID-19 pandemic means that many thousands of people globally will require long-term multi-disciplinary support and rehabilitation. Our COVID-19 patient partners highlighted issues including protracted recovery, multiple sequelae and perception of little post-discharge support.

There is little specific provision to support short-term recovery at home for COVID-19 survivors. Moreover, there are few, rehabilitation or structured support programmes for COVID-19 survivors who continue to have physical and mental health problems several months after hospital discharge. Where programmes exist, their potential benefit is unproven. Research is needed now to find out how best to help long-term COVID-19 survivors who have ongoing physical and mental health problems. Multi-disciplinary physical and psychological rehabilitation may be beneficial in improving people's quality of life. However, the size of the problem, now considered by some to be a rehabilitation pandemic [5], requires the testing of approaches to multi-disciplinary rehabilitation that can be delivered at scale.

Traditional centre-based NHS rehabilitation services do not have the capacity to support the numbers of people recovering from COVID-19 [1]. Resources are insufficient to deliver rehabilitation services within a traditional intensely supervised and facility dependent model of care. This, in combination with issues relating to continued restrictions on movement and extended closure of existing rehabilitation services, means it is imperative that alternative long-term support strategies are explored. 'Virtual' (on-line) rehabilitation may offer an alternative to traditional face-to-face rehabilitation. However, existing virtual rehabilitation platforms are not sufficiently specialised or developed to treat people recovering from COVID-19, and their clinical and cost-effectiveness has not been tested in randomised controlled trials (RCTs). Our patient partners, most of whom were not previously active on-line, said they had become confident in the use of on-line video technology during the pandemic.

1.2 Existing knowledge

People recovering from acute respiratory distress syndrome frequently develop substantial long-term morbidity [6]. Physical and psychological sequelae can affect quality of life (QoL) for years [7] with almost half of people not returning to work within 12 months of discharge [6]. Multiple studies investigating the 2002-2004 Severe Acute Respiratory Syndrome (SARS) epidemic showed reduced walking distance at three and six months compared to population norms [8]. One in six survivors had impaired pulmonary function at 24 months and SF-36 QoL domain scores were reduced [9]. Another study (N=189) found the prevalence of depression, anxiety and PTSD to be 14%, 18%, and

6% respectively [10]. A chronic post-SARS syndrome has been described, characterised by persistent fatigue, diffuse myalgia, weakness, depression, and sleep disturbance [11].

Early data from COVID-19 survivors shows a broadly similar pattern along with persistent cognitive impairment, and pulmonary hypertension in those with thromboembolic problems [1]. For the 45% of people hospitalised with COVID-19 in the UK who are estimated to require prolonged support from health and social care [4], a multitude of physical, psychological and social needs have been identified [1]. For hospitalised, but less severely affected patients, long-term physical and psychological consequences are also prominent [3]. A further feature is the disproportionate infection rate and progression to severe illness in Black, Asian and minority ethnic groups [12]. We have no data on whether ethnicity affects the prevalence or pattern of long-term sequelae from COVID-19.

Targeted exercise-based rehabilitation is beneficial for people with COPD [13] and survivors of SARS [14]. A quasi-experimental study (N=72) in COVID-19 survivors reported positive results on multiple outcomes [15]. On international trial registries, small RCTs (N=30-50) are assessing centre-based and on-line rehabilitation protocols for COVID 19 survivors. The majority aim to recruit participants immediately post-discharge, and none are UK-based. There are no large multi-centre RCTs assessing the clinical and cost-effectiveness of comprehensive, supervised, on-line, home-based physical and mental health rehabilitation. Choosing the optimum time to intervene to improve long-term outcomes is important. Early intervention targeting mental health problems is likely to be ineffective due to a high rate of spontaneous resolution [16]. Moreover, international guidance does not support early pulmonary rehabilitation for COVID-19 [1].

To tackle the multiple long-term physical and mental health consequences of COVID-19, it is clear that a complex, multi-disciplinary, physical and psychological rehabilitation intervention should be tested. Importantly, this must be delivered at the appropriate point in the recovery timeline. It must also be cost-effective and deliverable at scale whilst adhering to continued general population infection control measures. Further, it must address ethnic and cultural health inequalities.

1.3 Hypothesis

Research question: What is the clinical and cost effectiveness of an intensive, on-line, supervised, group, home-based rehabilitation programme that supports long-term physical and mental health recovery for people discharged from hospital (>3/12) after COVID-19 infection?

Aim: To assess the clinical and cost-effectiveness of the 'Rehabilitation Exercise and psycholoGical support After covid-19 InfectioN' (REGAIN) intervention compared to best-practice usual care (single session of advice only) for people recovering from COVID-19.

Objectives: To run a definitive multicentre RCT testing the clinical and cost-effectiveness of REGAIN vs. a single session of advice, including:

1. A intervention development phase to confirm feasibility, refine online intervention delivery and manualised practitioner training, and prepare study set-up;
2. An internal pilot, with formative process evaluation, to test recruitment and study procedures
3. A main study with embedded process evaluation.

1.4 Need for a study

To date, research has understandably focused on the immediate need for life-saving health interventions. Research has addressed the basic biology and epidemiology of COVID-19 and concentrated on early efforts to develop evidence-based treatments and vaccination. Early evidence that some treatments, such as dexamethasone, effectively reduce mortality in selected patients, emphasises the importance of longer-term support for the increasing proportion of those affected who survive to hospital discharge [17].

The large number of people affected over a short time frame means that many people in the UK are now facing a rehabilitation challenge. This has physical, psychological and economic consequences at individual and societal levels. While interventional research rapidly develops, the proposed REGAIN intervention has the potential to guide recovery and re-entry to economic productivity for those living with the longer-term consequences of COVID-19.

Long-term rehabilitation interventions are not currently offered to COVID-19 survivors. To our knowledge, there are no rehabilitation interventions currently being tested in the UK for people who have not fully recovered more than three months after hospital discharge. This group are likely to require intensive support as they may be at high risk of chronic physical and mental health problems.

Many COVID-19 survivors return to normal activities within a few weeks [4]. Thus, universal early intensive rehabilitation only has the potential to help a sub-set of people. Selecting those people who have not recovered after three months is likely to be a more efficient and cost-effective approach to rehabilitation. Furthermore, it may only be after a protracted recovery that many people, who were previously well, are likely to require, and be accepting of, a psychologically informed intervention.

We need to deliver this study rapidly to inform long-term care for COVID-19 survivors and to achieve the greatest benefit for patients and society. To do this efficiently at a time when restrictions to normal life are likely to continue for some time, and to take advantage of the recent shift in acceptability of virtual health care, REGAIN will be run completely on-line. On-line recruitment, outcomes assessment and intervention delivery mean we can have a national sampling frame, approaching very large numbers of potential participants in a short period.

If either of the interventions tested in this trial are effective, we will have an intervention suitable for immediate implementation nationally and internationally. Implementation of a successful programme has the potential to substantially reduce the chronic burden of COVID-19 in a large number of survivors, who, in the current unique pandemic environment, may not have access to normal social and primary/community care support. Apart from the direct benefits for those concerned, improving the general health of survivors has the potential to reduce demand on health and social services more widely and improve economic productivity.

1.5 Ethical considerations

The study will be conducted in full conformance with the principles of the Declaration of Helsinki and to Good Clinical Practice (GCP) guidelines. It will also comply with all applicable UK legislation and University of Warwick Standard Operating Procedures (SOPs). All data will be stored securely and held in accordance with the Data Protection Act 2018.

Study participants will be enrolled via two routes of entry, either by direct approach of the patient by UHCW NHS Trust or a participant identification centre (PIC) or by self-referral. Before approaching potential study participants, each site will ensure that the local conduct of the study has the agreement of the relevant NHS Trust Research & Development (R&D) department and written confirmation is received by Warwick Clinical Trials Unit (WCTU).

Direct approaches to potential participants made by study sites will be from clinical care teams which may be supported by members of the REGAIN team should site capacity deem this necessary. All identifiable data will be held within NHS sites.

Relevant data, including identifiable data, will be entered directly by participants into a secure online database provided by WCTU, although in some instances, data may be entered into the database by study staff at UHCW or WCTU during telephone calls with study participants. These data will be considered as source data for the study.

We will ensure that staff undertaking study recruitment are trained in GCP and consent procedures.

For the symptoms sub-study, identification sites will collate a spreadsheet, pseudonymised by screening ID, of routinely collected hospital data in relation to COVID-19 admission, (length of stay and ventilation type). Consent will be sought from all study participants prior to this data being sent to WCTU.

Any routine data collected for the symptoms sub-study and GP records (if collected for particular participant) will also be considered source data. Direct access to source data will be granted to authorised representatives from the sponsor, host institutions and the regulatory authorities to permit study related monitoring, audits and inspections.

Study staff will ensure that participants' anonymity is maintained. Participant identifiable information collected for the study will be stored securely on the electronic database. REGAIN practitioners at UHCW will also keep paper records of participant contact details and medical notes. All data will be stored securely and will only be accessed by study staff and authorised personnel. Paper records at UHCW will be stored securely in locked filing cabinets. The study will comply with relevant UK data protection legislation, which requires data to be pseudonymised as soon as it is practical to do so. Identifiable data will be deleted 12 months after the completion of the study (last follow-up for last participant).

One ethical consideration is that people of different ethnicities can take part in the study. Participants who are not fluent in spoken or written English will be eligible to take part. Participant information sheets and consent forms will be translated into the following languages; Bengali, Gujarati, Urdu, Punjabi and Mandarin. When confirming consent for those not fluent in English, a bilingual researcher will speak to the participant to ensure a full explanation of the study and to confirm understanding. An NHS accredited translator will be included in the one-to-one advice consultation (control arm) and the individual assessment (intervention arm). On-demand online videos will also be translated in those languages mentioned above. Participants who are not fluent in English will be encouraged to attend live online exercise sessions with a friend or relative who can translate for them. For the psychological support sessions we will arrange bespoke small online group sessions with a REGAIN practitioner and NHS accredited translator. A core data outcome set including the PROMIS® 29+2 Profile v2.1 (PROPr) and EQ-5D-5L questionnaires will be collected orally by a bilingual researcher, where necessary, to ensure that those not fluent in English are able to contribute participant reported outcomes to the study.

Mindful of the likely high prevalence of case level mental health symptomology in this population, REGAIN practitioners seeing people in both arms of the study will be provided with selected findings from the baseline questionnaires. Those with suspected mental health symptoms (depression/anxiety/PTSD), based on high scores reported on one or more of the HADS Anxiety sub-scale, HADS Depression sub-scale, and IES-6 within the baseline questionnaire, will be flagged and patients will be directed to their GP for advice as per the participant information sheet and also by the REGAIN practitioner. Participants with suspected mental health disorders (based on symptom score cut-points) who do not attend their first treatment session will be contacted by the REGAIN study team via email or letter and advised to see their GP, even if they no longer wish to take part in the study. We will provide all GPs with a letter via email or post explaining their patient is taking part in the study and notification of their treatment allocation. This letter will also provide the baseline screening scores for two measures only, the HADS and IES-6 questionnaires. Additional reports will be provided to GPs at the 3, 6 and 12 month follow-up time points for those patients who score highly on one or more of the HADS Anxiety sub-scale, HADS Depression sub-scale or IES-6 questionnaires. Any participants who have not been previously contacted to discuss their questionnaire screening scores will be called by a member of the REGAIN study team.

1.6 CONSORT

The study will be reported in line with the CONSORT (*Con*solidated *S*tandards *o*f *R*eporting *T*rials) statement [18].

2. STUDY DESIGN

2.1 Study summary and flow diagram

REGAIN is a multi-centre, randomised controlled study testing the clinical and cost-effectiveness of the REGAIN intervention vs. best practice usual care, including:

1. An intervention development phase, to confirm feasibility, refine online intervention delivery and manualised practitioner training, and prepare study set-up
2. An internal pilot, with formative process evaluation, to test recruitment and study procedures
3. A main study with embedded process evaluation.

Around 20 NHS trusts, prioritising ethnically diverse localities, will be set up as Participant Identification Centres (PIC). Participants will also be identified by UHCW NHS Trust.

Participants may also be identified by NHS Digital; At the time of writing, the Secretary of State for Health and Social Care has issued NHS Digital with a Notice under Regulation 3(4) of the Health Service (Control of Patient Information) Regulations 2002 (COPI) to require NHS Digital to share confidential patient information with organisations entitled to process this under COPI for COVID-19 purposes. As such, whilst this COPI Notice is in effect, participants may be identified by NHS Digital for inclusion in the REGAIN study in support of the pandemic response. .

The study team based at UHCW and Warwick Clinical Trials Unit (WCTU) will recruit participants who have registered their interest. The intervention and control sessions will be led by staff at the UHCW community exercise rehabilitation centre (Atrium Health, Coventry).

Study overview: UK based adults admitted to hospital with COVID-19 who were discharged more than three months previously will be identified from hospital records at UHCW NHS Trust or PIC

sites or may be identified by NHS Digital who have access to secondary care records UK wide under the COPI Notice. Contact will be made with patients discharged over three months previously; the REGAIN team will support mailout activities at site as needed based on capacity and timelines. Confirmation of clinical status via hospital and NHS systems will be performed immediately prior to the mail out to ensure that patients have not died since their hospital discharge. People with substantial ongoing health problems after COVID-19 will also be able to self-refer to the study. Self-referrals may be identified through provision and/or display of REGAIN flyers in primary and secondary care NHS COVID clinics, GP practices and pharmacies. Self-referrals may also be identified by promotion of study through local/national media/social media, relevant charities and on the study website. The study will not recruit two patients from the same household.

Those with substantial ongoing COVID -19 sequelae, as defined by the participant, who are eligible for the study will be invited to participate.

We aim to recruit 535 participants, who will be randomised to the REGAIN intervention or best practice usual care only on a 1.03:1 basis using a computer-generated randomisation sequence, performed by minimisation and stratified by age, level of hospital care (ICU/HDU or ward), and case level mental health symptomology based on scoring of the HADS Anxiety sub-scale, HADS Depression sub-scale and IES-6.

Outcomes will be assessed at baseline pre-randomisation and at three, six and 12 months post-randomisation. The primary outcome will be HRQoL measured using the PROMIS® 29+2 Profile v2.1 (PROPr) at three months post-randomisation. Data will be collected directly from study participants using online data collection via the secure REGAIN database.

Intervention development phase: A small number of PPI volunteers will be involved in the study intervention development in advance of delivering the study to participants. These PPI volunteers will not be allocated a REGAIN study number and no data from these participants will be analysed as part of the study outcomes. The purpose of this intervention development phase will be to refine and test the online delivery of intervention and control materials, including participant and practitioner manuals, and staff training procedures, and to commence preparation for study set-up. This will allow us to confirm the viability of all aspects of the study and make final alterations prior to the internal pilot.

Internal pilot: In a one-month internal pilot (n=35), recruiting from UHCW NHS Trust and multiple PIC sites and running seamlessly into the main study, participant recruitment and retention will be confirmed. This will also provide provisional data on the fidelity of the intervention, its safety, and participant compliance and experiences.

Figure 1 Study flow diagram

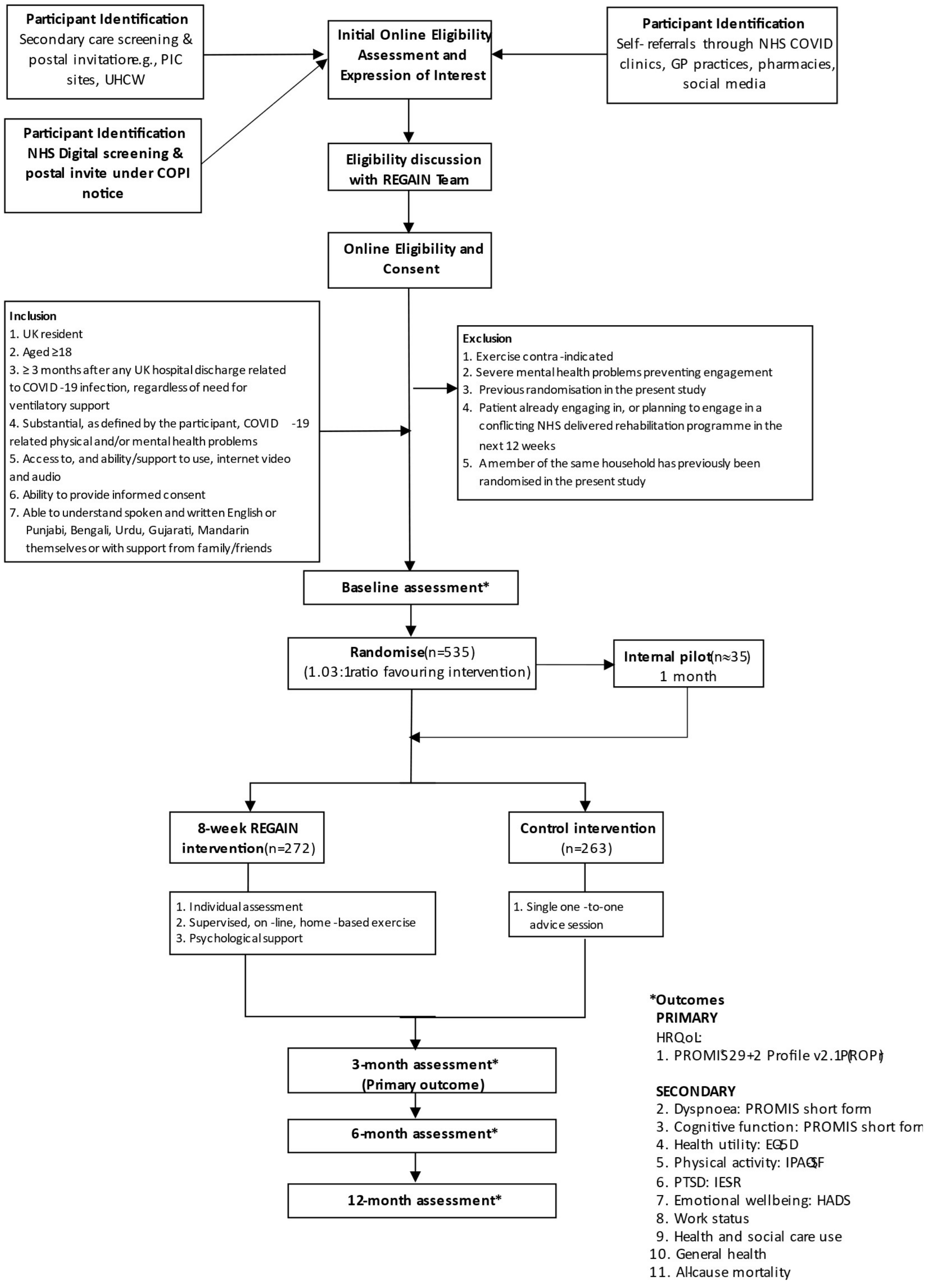

2.2 Aims and objectives

The aim of this study is to assess the clinical and cost effectiveness of an intensive, on-line, supervised, group, home-based rehabilitation programme (the REGAIN intervention) compared to best practice usual care (single advice session only), to support long-term physical and mental health recovery for people discharged from hospital more than three months after COVID-19 infection.

2.2.1 Primary objective

The primary objective of this study is to determine if the REGAIN rehabilitation intervention improves HRQoL at three months post-randomisation compared to best-practice usual care in patients with ongoing COVID-19 symptoms.

2.2.2 Secondary objective

Secondary objectives of the study are to determine if the REGAIN intervention compared to best-practice usual care in patients with ongoing COVID-19 symptoms impacts on the following outcomes over 12 months:

1. HRQoL
2. Dyspnoea
3. Cognitive function
4. Health utility
5. Physical activity
6. PTSD symptom severity
7. Depressive and anxiety symptoms
8. Work status
9. Health and social care resource use
10. General health
11. All-cause mortality.

2.2.3 Symptoms sub-study objective

To explore the relationship between personal characteristics and in-hospital care, and subsequent ongoing COVID-19 symptoms and other health problems.

2.2.4 Process Evaluation objective

1) To explore the experiences of participants in the intervention and control groups, including enablers of, and barriers to, lifestyle change amongst participants.

2) To highlight any contextual issues that may affect the outcome or delivery of the study and/or intervention.

2.3 Outcome measures

2.3.1 Efficacy

Primary Outcome:

Health-related quality of life (HRQoL) measured using the PROMIS® 29+2 Profile v2.1 (PROPr) at three months post-randomisation. This measure is part of a portfolio of outcomes developed and validated by the National Institute for Health (NIH) (USA); the Patient-Reported Outcomes Measurement Information System. It is a reliable generic outcome measure validated for on-line use [19-21] generating a single overall score plus physical function, anxiety, depression, fatigue, sleep disturbance, social roles/activities, pain interference, cognitive function and pain intensity sub-scales.

Justification for timing of primary outcome

Long-term outcomes are important, however, any intervention effects will be maximal soon after completion of the intervention. We have set our short-term follow-up at three months as we are confident that those randomised to the REGAIN intervention will complete the eight-week treatment phase in this time period. If there is no evidence of effect at three months, then a meaningful effect at one year is unlikely. Assessing the primary outcome at three months after randomisation is more efficient than seeking an effect at one year, as attrition will be lower.

Secondary Outcomes:

The following outcomes will be measured at three, six and 12 months post-randomisation.

1. HRQoL: PROPr
2. Dyspnoea: PROMIS dyspnoea severity Short Form [21]. Exertional dyspnoea is a commonly reported symptom in COVID-19 survivors, so we have added specific questions to the longer HRQoL PROMIS measure.
3. Cognitive function: PROMIS Neuro-QoL Short Form v2.0 - Cognitive Function [21]. In light of the apparent high incidence of cognitive impairment in COVID-19 survivors we have added additional PROMIS questions, to obtain a specific measure of cognitive function.
4. Health utility: Euroqol EQ-5D-5L [22]. Validated, generic HRQoL measure consisting of five dimensions, each with five levels. Each combination of answers can be converted into a health utility score. It has good test-retest reliability, is simple to use, and gives a single preference-based index value for health status that can be used for cost-effectiveness analysis.
5. International Physical Activity Questionnaire (IPAQ short-form). A well-established activity measure reported as metabolic equivalent task (MET)-minutes per week derived from duration of walking, moderate and vigorous exercise [23]
6. PTSD symptom severity: The Impacts of Events Scale-Revised (IES-R) a 22 item self-report measure of difficulties people sometimes face after stressful life events. It has been widely used in studies of survivors of ICU admission, including COVID admissions. It is part of recommended outcomes for studies of respiratory failure survivors [24-26]. A score of ≥11 on the IES-6, an abbreviated version extracted from the longer 22-item IES-R, will be taken to be indicative of case level disorder.
7. Depressive and anxiety symptoms: Hospital Anxiety and Depression Scale (HADS). A 14-item questionnaire from which anxiety and depression subscales can be derived. 7 item sub-score values ≥11 points identify case-level anxiety/depression. Commonly used and well validated measure in clinical populations [27].
8. Work status: Time lost from work (paid/unpaid) and patient-borne health costs.
9. Health and social care resource use: participant self-report and NHS records. The primary health-economic analysis will concentrate on direct intervention and healthcare/personal social services costs, while wider impact (societal) costs will be included within the sensitivity analyses. Participants will complete resource use questionnaires at all follow-up points, to

collect resource use data associated with the interventions under examination. We will request a copy of the participant's medical record from their GP at the end of the study follow-up if the participant has not responded to the 12-month follow-up or if we know the participant has died. This will provide information on GP consultations and include copies of any hospital discharge letters allowing us to accurately cost in-patient care costs. Where appropriate we will triangulate data from GP records and participant self-report to achieve a robust estimate of health service activity. Consent will be obtained for accessing GP records.

10. General health – Participant self-reported measurements of current overall health and comparison of current health to health 12 months prior.
11. Death measured using GP.

Follow-up: Patient reported outcomes will be collected online at baseline pre-randomisation, and at three, six months and 12 months post-randomisation. Participants will receive an email notification and/or text message to ask them to complete the online questionnaires at each follow-up time point. In the case of non-response, first a reminder message will be sent, second: a reminder call will be made and third: a data collection call will be made with priority on collecting the two key outcomes, the PROPr (primary outcome) and EQ-5D-5L.. Fluency in English is not an inclusion criterion for this study. For those not fluent in English, we will aim to collect all outcomes (or as many as possible) verbally at each follow-up. As a minimum, a core data outcome set including the PROMIS® 29+2 Profile v2.1 (PROPr) and EQ-5D-5L questionnaires will be collected orally by a bilingual researcher, where necessary, to ensure that those not fluent in English are able to contribute participant reported outcomes to the study. The EQ-5D-5L is well validated for verbal administration.

Long-term follow-up: Consent will be sought from participants to hold their personal data, and at the end of the 12-month follow-up period, to request a copy of the participant's medical record from their GP. This will only be requested if the participant has not responded to the 12-month follow-up or if we know the participant has died. This will provide information on GP consultations and include copies of any hospital discharge letters allowing us to accurately cost in-patient care costs. Where appropriate, we will triangulate data from GP records and any participant self-report to achieve a robust estimate of health service activity and mortality.

2.3.2 **Symptoms sub-study**

Study sites (PIC sites and UHCW) identifying patients will record information on patient hospital admission data including length of hospital stay and ventilation type. This will be pseudonymised using a screening ID number assigned to each patient by the study site. Any patients approached by a study site will provide their screening ID number and using this screening ID number, for those patients consenting to the study, WCTU will request pseudonymised data for that individual from the study site that approached the participant. Ongoing COVID-19 symptoms will be collected during the initial online eligibility assessment. This will allow us to compare selected factors including patient characteristics and COVID-19 admission characteristics, and ongoing COVID-19 symptoms profile of those who take part in the study. These data will not be collected from those patients entering the study via the self-referral route or those invited by NHS Digital.

2.4 Eligibility criteria

Patients are eligible to be included in the study if they meet the following criteria:

2.4.1 Inclusion criteria

1. UK resident
2. Aged ≥18;
3. ≥ 3 months after any UK hospital discharge related to COVID-19 infection, regardless of need for critical care or ventilatory support;
4. Substantial, as defined by the participant, COVID-19 related physical and/or mental health problems;
5. Access to, and ability/support to use, email, text message, internet video, including webcam and audio;
6. Ability to provide informed consent;
7. Able to understand spoken and written English or Bengali, Gujarati, Urdu, Punjabi, Mandarin themselves or with support from family/friends.

2.4.2 Exclusion criteria

1. Exercise contraindicated*
2. Severe mental health problems preventing engagement**
3. Previous randomisation in the present study
4. Patient already engaging in, or planning to engage in a conflicting NHS delivered rehabilitation programme in the next 12 weeks
5. A member of the same household has previously been randomised in the present study

* As advised by a clinical member of the research team or REGAIN practitioner
** Adjudged by a clinical member of the research team or the REGAIN practitioner

2.5 Participant identification / Screening

Patients will be identified via three routes: (i) screening of hospital discharge data to identify potential participants for contact by mail at PIC sites; (ii) via self-referral; (iii) screening of hospital discharge data to identify participants for contact by mail by NHS Digital under the COPI Notice

Patient Identification

Clinical care teams at UHCW NHS Trust and each PIC site (NHS hospital trust) will screen hospital discharge data and identify potential participants for contact by mail. NHS Digital will similarly screen hospital discharge data and identify potential participants for contact by mail. Both will send potential participants an infographic invitation flyer and invitation letter, with an allocated screening ID number, which will direct potential participants to the study website. These resources will be brief, providing only the most important detail required, and will be written in plain English. The REGAIN team will support mailout activities at site as needed based on capacity and timelines. The invitation letter and REGAIN study website will instruct potential participants to read the PIS, and if they are interested in taking part in the study, to access the online database via the 'suitability check link' to register. On each resource there will be a sentence in each of the five specified non-English languages directing the potential participant to the study website where the participant information sheet (PIS) and consent form will be available in their preferred language. For those whose first language is not English, there will be an option to request a phone call from a bilingual

research associate. This option will be written in five languages on the study website: Bengali, Gujarati, Urdu, Punjabi and Mandarin.

The online database will ask potential participants a series of screening questions to determine their initial eligibility for the study.

If a potential participant is not eligible for the study, a message will appear on screen to inform them that the REGAIN study is not suitable for them. These people will be advised to refer to the NHS ‘yourcovidrecovery’ website.

If the participant is initially considered eligible, they will be asked to enter their contact details including their first name, surname, address, post code, telephone number(s), email address, GP name and GP address. A GP address must be provided in order for the potential participant to register interest into the study. This is required so that the participant’s GP can be contacted if any medical concerns are raised during the initial eligibility and consent telephone call. The potential participant will be instructed that a member of the REGAIN team will be in touch via telephone to confirm their suitability for the study.

Self-referral
A REC-approved infographic invitation flyer will be used to promote the study. These infographic invitation flyers will be provided to relevant primary and secondary care NHS COVID clinics for staff to hand out to potential participants. The flyers will also be displayed and available at GP practices and pharmacies. The study will also be promoted though local/national media/social media, relevant charities and on the study website. People suffering from ongoing COVID-19 related symptoms following hospital discharge will be able to self-refer. Self-referred patients will be directed to the REGAIN website and will follow the same process as described above for site referrals. In the event that verification of hospitalisation with COVID-19 is needed for self-referred patients, the patients GP will be contacted with evidence of patient consent for this verification provided. If the participant is able to provide a hospital discharge letter for verification by the REGAIN team, this will be accepted as evidence and the GP will not be contacted. Copies of hospital discharge letters will not be held by the REGAIN team.

2.6 Eligibility and informed consent

When a potential participant has registered their eligibility and provided their contact details for the REGAIN study via the online database, the WCTU REGAIN study team and the REGAIN site team based at UHCW will receive an alert that a new potential participant has registered their interest. A clinical member of the REGAIN team, (listed on the study delegation log), will then telephone the potential participant on their main telephone contact number. The REGAIN team member will conduct a full eligibility screen with the potential participant and complete an online eligibility form for the potential participant. The REGAIN team member will ensure the potential participant has read the PIS, understands what is involved and has had the chance to ask any questions before starting the eligibility questions.

If the potential participant is eligible for the REGAIN study, they will automatically receive a link via text or email (whichever they have specified is their preference) to an electronic consent form. The team member will explain the purpose of the consent form and summarise the key points. The patient will be able to complete the consent form in their own time, although the link will only be active for three weeks from the date sent. Upon clicking the link to the consent form, the

participant will be issued with an authentication code via text or email, ensuring only the intended patient can access the consent form via the sent link. Potential participants will need to confirm they have read each of the consent items before agreeing to take part in the study. A copy of the completed consent form will then be sent to the patient via email. Once the consent form has been completed, the participant will be sent another link to access the baseline questionnaire.
If the potential participant has self-referred into the study however, they will be made aware that their eligibility can only be confirmed following GP verification of their hospitalisation. These patients will receive a link to an electronic consent form as above, but following its completion, a member of the REGAIN team will contact the patients GP for verification. These patients will only be sent a link to access the baseline questionnaire if their hospitalisation is verified by their GP. If not, they are ineligible to join the study and proceed no further. A member of the REGAIN team will call the patient to inform them of this. An email will be sent if the patient cannot be reached by telephone.

Once both the consent form and baseline questionnaires have been completed by the patient, they will be automatically randomised into the study by the online system. The participant will receive a notification confirming that they have been successfully randomised and will be informed that the REGAIN team will be in touch shortly to let them know their allocation and to arrange their first appointment.

Pregnancy is not an exclusion criterion for REGAIN. These potential participants will be recruited to the study if eligible and participants who confirm pregnancy following enrolment will remain in the study. All participants randomised to the intervention arm, including those who are pregnant, will receive a one-to-one consultation with a REGAIN practitioner where exercise will be tailored to their ability. The exercise intervention is highly adaptable thus deemed safe for those who are pregnant. All participants will be monitored for adverse events as per Section 4.

GP notification: After randomisation, the participant's GPs will be informed by letter that they are taking part in the study, informed of the participant's baseline HADS and IES-6 questionnaire scores, and notified of which treatment arm they have been allocated to.

Responsibility: The PI at UHCW or Trial Manager at CTU will retain overall responsibility for informed consent and will ensure that any person delegated responsibility to participate in the informed consent process is duly authorised, trained, qualified and competent.

When confirming consent for those not fluent in English, an NHS accredited translator or bilingual researcher will be present to ensure that participants receive a full explanation of the study and to confirm their understanding, according to Warwick SOP 7.

New information: Any new information that arises during the study will be reviewed by the TSC. If this new information may affect participants' willingness to take part in the study, it will be communicated to all participants. Participants will be contacted by a member of the REGAIN team and asked whether they still wish to continue participating in the study. Participants will be provided with an updated PIS and asked to complete a revised consent form as necessary.

Incidental findings: Incidental findings relating to participants' medical conditions or general health, will be discussed with the managing consultant, and communicated to the participant as required. We have a clearly defined process for handling incidental findings from our questionnaires that assess anxiety, depression and post-traumatic stress (potential case-level mental health diagnosis). Our psychiatric and health psychology co-investigators have identified cut-off points that are pre-

programed into the trial database. Should participants exceed these clinical cut-offs on completion of the online questionnaires, the trial staff and REGAIN intervention/control practitioners (clinical exercise physiologists/physiotherapists) will be alerted. This information will be discussed with the participant during their initial one-to-one appointment with a REGAIN practitioner at baseline.

The participant will be advised to make an appointment with their GP to discuss the findings and will be informed that a letter will also be sent to their GP. If the team is unable to make contact with the participant for the one-to-one appointment, the participant and GP will both still receive a letter regarding their mental health scores. The participant's letter will inform the participant that their GP will receive a letter.

If case level mental health disorder is identified on a follow up questionnaire and the participant has previously discussed their mental health scores with a team member, they will be a sent letter asking they seek GP advice and informing their GP will also receive a letter.

If case level mental health disorder is identified on a follow up questionnaire and the participant has *not* previously discussed their mental health scores with a team member, a separate call with a member of the REGAIN team will be arranged. The participant will be advised to make an appointment with their GP to discuss the findings and they will be informed their GP will also receive a letter. This will be relayed to the participant on the call and also in a letter. If the team is unable to make contact with the participant for this call the participant and GP will both still receive a letter regarding their mental health scores. The participant's letter will inform the participant that their GP will receive a letter.

Participants will be provided with contact details for the REGAIN trial team based at UHCW should they wish to report any concerns.

Decline/withdrawal: Participants will have the option to withdraw from the study and/or the intervention at any time, if for any reason they change their mind without giving reasons and without prejudice to any further treatment. This will be recorded on a withdrawal form. The right of a potential participant to refuse participation without giving reasons will be respected and recorded on the withdrawal form. A reason will be documented if participant is willing to offer one.

Willingness to continue in the study will also be monitored and recorded throughout the intervention period by practitioners conducting the interventions.

2.6.1 Consent for qualitative interviews

At the beginning of the study, participants will be asked for their consent to be contacted at a later stage about an interview with a researcher. Although consent will be recorded for everyone on entry to the REGAIN study, only some participants will be contacted for interview. If they are selected for interview, participants will receive an email inviting them to consider the interview study and be directed to the study database where they can read the interview PIS. If they do not respond to the invite, a member of the REGAIN team will make contact to answer any questions they may have and provide support with following links and with online forms where required. If the participant declines the interview and provides a reason as to why, this will be recorded. If they choose to participate, they can complete an online interview consent form linked to the study database. Following receipt of the consent form, a member of the WCTU study team will contact the participant to discuss the interview study, answer any questions they may have, and if they remain happy to proceed, arrange a date for the interview to take place. The interviewer will

confirm the consent form has been completed before the interview is conducted by telephone or video call.

2.6.2 Consent for photographs and video clips

At the beginning of the study, participants will be asked if they consent to have photographs or short video clips taken during the live exercise sessions and support sessions for use at conference presentations or for study publicity. If they consent to this, selected participants/groups will be approached by a study practitioner and an appropriate time and date will be arranged for photos/recordings to be taken.

2.6.3 Consent for qualitative practitioner interviews

The REGAIN study will be delivered online from a single central venue – UHCW NHS Trust. There will be 5 practitioners involved in delivery of the study (control and intervention). At the end of their time on the study, and with their consent, we will interview all practitioners involved in the study. If more practitioners are involved in delivery, we will also interview them. Interviews will be conducted by a qualitative research fellow from Warwick CTU.

2.7 Site Staff Training

Staff training will be documented on training logs held at WCTU and UHCW. Study responsibilities will be documented on delegation logs to be held at WCTU and UHCW. The CI will retain overall responsibility for conduct of the study.

Intervention practitioners: Practitioners delivering the REGAIN intervention will be Clinical Exercise Physiologists or Physiotherapists with appropriate professional registration, relevant continued professional development (CPD), and good clinical practice (GCP) training. All practitioners will be based at UHCW and an exercise lead will be responsible for ensuring study procedures are followed and standardised for intervention delivery.

REGAIN training: All intervention practitioners will undergo one day of REGAIN intervention training. This training will ensure an appropriate level of clinical knowledge and skills for exercise rehabilitation in COVID-19 patients. Training will be delivered by a health psychologist, to upskill practitioners on delivery of the psychological component of the intervention. Training will be supplemented with a comprehensive practitioner intervention manual. Access to health psychology expertise and support will be maintained and monitored throughout the duration of the study. The exercise lead at UHCW will be responsible for ensuring additional practitioners are appropriately trained and familiarised with the manual. Full training will be provided by the REGAIN research fellow and health psychologist for new staff, as needed. Should any clinical (physical or mental) issue require escalation, REGAIN practitioners will follow the appropriate local clinical guidelines.

REGAIN Practitioner manual: This detailed manual will guide practitioners through each component of the intervention, with graphics, flowcharts and detailed written instructions. It will also include general information about the study, key components of GCP, and contact details of the study team. The content will reflect information delivered during the training for REGAIN intervention practitioners.

Exercise intervention: To enhance practitioners' knowledge of exercise prescription, ensuring intervention efficacy and safety, the manual will provide an overview of key evidence and exercise

guidance. To provide a level of standardisation, parameters within which the exercise intervention should be delivered and progressed will be detailed.

Psychological support intervention: The manual will give a detailed description of each psychological topic, with hints and tips of questions to ask, and the aims of each session. The content will map onto the intervention participant manual, allowing the practitioner to tailor the discussion.

2.8 Randomisation

2.8.1 Randomisation

Pre-randomisation eligibility checks will be carried out to ensure that potential participants meet the eligibility criteria and are not randomised in error. Consent for entry into the study must have been completed prior to randomisation. Subjects will be randomised once they have been registered as eligible for randomisation on the web-based system and completed their baseline questionnaire.

Participants with case level mental health disorder, identified from baseline HADS and IES-6 questionnaires (screening scales for anxiety, depression, PTSD) will be directed to their GP for treatment. This will be included in the PIS. They will continue in the study intervention as long as the REGAIN practitioner and/or the participant consider that their mental health does not preclude engagement with interventions.

Randomisation will be undertaken automatically by the system following completion of the baseline questionnaire using a computer-generated randomisation sequence, performed by minimisation and stratified by:

1. age (i. <65; ii. ≥65),
2. level of hospital care (i. ICU/HDU; ii. ward),
3. case level mental health disorder (i. IES-6 PTSD score ≥11/24 **or** HADS Anxiety sub-score ≥11/21 **or** HADS Depression sub-score ≥11/21; ii. IES-6 PTSD score <11/24 **and** HADS Anxiety sub- score <11/14 **and** HADS Depression sub- score <11/21).

Participants will be randomised strictly sequentially at study level.

2.8.2 Post-randomisation withdrawals and exclusions

Participants may decline to continue involvement in the study at any time, without prejudice. This will not affect the standard of care they receive. For participants withdrawing from the study, data obtained prior to the point of withdrawal, will be retained for the final analysis unless explicitly withdrawn at the participant’s request. For participants who withdraw, a withdrawal CRF will be completed.

Participants may be withdrawn from the study, at any time, at the discretion of the chief investigator, practitioners based at UHCW, or the Trial Steering Committee due to safety concerns.

2.9 Study interventions

2.9.1 **Study treatment(s) / intervention(s)**

The REGAIN study will be delivered online from a single central venue – UHCW NHS Trust. There will be approximately 5 REGAIN practitioners (Clinical Exercise Physiologist/Physiotherapists) based at UHCW who will deliver the study interventions as described below. Additional REGAIN practitioners may be identified during the course of the study.

2.9.2 **Best practice usual care (Control) intervention**

A thirty-minute, on-line, one-to-one consultation with a REGAIN practitioner, trained and supported by a Health Psychologist during the study. All study participants will be provided with a 'Your Covid recovery guide' which incorporates some components of freely available on-line information and advice published by NHS England (https://www.yourcovidrecovery.nhs.uk/) as well as directing participants to other relevant online resources.

If case level mental health disorder (depression/anxiety/PTSD) is identified from baseline questionnaires, participants will be advised to contact their GP for treatment/advice.

Participants with suspected mental health disorders who do not attend their one-to-one consultation will be contacted by email or letter and advised to see their GP. GPs will receive a letter via email or post for participants where case level mental health disorder is identified, specifying scores reported on the questionnaires. The letter will not indicate a diagnosis, rather, will present the questionnaire data allowing the GP to decide on appropriate treatment.

We recognise the challenge of recruiting to studies where the usual care arm receives no additional treatment or care, despite understanding issues around equipoise. Our patient partners consistently raise this issue. Therefore, for our control arm, the intervention can be described as 'best-practice usual care', in the form that is currently recommended by the NHS (yourcovidrecovery.nhs.uk) and also an individual practitioner consultation, with general advice on safe and effective physical activity. A 30-minute consultation will allow practitioners to discuss individualised ways in which participants can undertake physical activity at home. Participants will not be provided with a structured exercise plan, rather they will be advised on ways in which physical activity can be safely and effectively incorporated into their everyday lives as well as being directed to reputable freely available on-line resources. No specific psychological techniques will be used to support this. Doing this allows us to offer the usual care group a standardised form of best current practice, whilst retaining the aim of the study comparing outcomes in people who receive comprehensive support, with people who do not. This approach of comparing two study interventions also reduces the risk of resentful demoralisation in the control group which might introduce bias [35].

2.9.3 **REGAIN Intervention**

The REGAIN intervention has three components:

1. **Individual assessment:** One-hour, on-line, one-to-one assessment with a REGAIN practitioner (Clinical Exercise Physiologist/Physiotherapist), who will be trained and supported by a health psychologist during the study, to holistically assess participant needs, introduce the programme, and provide individualised exercise advice. All participants will also be directed to freely available on-line programmes published by NHS England (https://www.yourcovidrecovery.nhs.uk/).

Participants with case level mental health disorders (depression/anxiety/PTSD), as identified from baseline questionnaires (IES-6 score ≥11; HADS Anxiety score ≥11; HADS Depression score ≥11), will be directed to their GP for treatment/advice. These symptomatic patients will continue in the study intervention as long as the practitioner considers their mental health problems would not preclude engagement.

Participants with suspected mental health disorders who do not attend their first treatment session will be contacted by email or letter and advised to see their GP. GPs will receive a letter via email or post for participants where case level mental health disorder was met specifying screening scores for HADS and/or IES-6.

2. **On-line, home-based, exercise rehabilitation:** Up to 30 minutes exercise two to three times per week for eight weeks; individualised and progressive multi-modality exercise at a manageable intensity (regulated with breathlessness and perceived exertion scales).

   Participants will be encouraged to attend one live on-line group exercise session every week for eight weeks led by a REGAIN practitioner, using equipment-free exercise to improve cardiovascular fitness, strength, balance, and co-ordination. These sessions will be undertaken in discrete groups. Participants will remain in the same group for the 8 week programme. Where possible, some groups will be single sex.

   For the remaining 1-2 exercise sessions per week, participants will access online, pre-recorded sessions of different intensity levels and exercise types=.

3. **Psychological support:** Over the eight-week intervention period, participants will attend six on-line group sessions each lasting for up to one hour, led by a REGAIN practitioner who will be trained and supported by a health psychologist during the study. Core theoretical principles used to inform the psychosocial content, structure and delivery include the bio-psychosocial model of behaviour change [28, 29], Michie's behaviour change wheel and taxonomy [30], Michie's COM-B model (Capability, Opportunity and Motivation), and psychological theories of self-efficacy (perceived confidence in ability to engage and implement the strategies learnt) [31], cognitive behaviour-change, and motivational interviewing [32]. The logic model for the psychological intervention can be found in Appendix 1.

The group support element draws on social learning principles promoting behaviour change through peer support. It is necessary to engage participants in the thought processes needed to interpret their own experiences of COVID-19 by providing time for discussion and reflection and then a summary of key information to promote cognitive functioning. We will explore expectations which may include the meaning of recovery, impact on social networks and relationships, including family and friends and goals to rebuild life to promote executive functioning (Capability). These sessions will allow participants to engage in the programme from the safety and convenience of their chosen location.

Each week will cover different topics providing strategies to help recovery from the effects of Covid-19. We will incorporate motivational interviewing techniques to promote direct behaviour change through awareness and management of emotional responses to participants' own experiences of COVID-19 which may include fear, stress and low mood. Education will be combined with cognitive behavioural approaches to action management and change, with online worksheets between

sessions to consolidate learning. For the mental health disorders likely to be common in this group of patients (depressive and anxiety disorders including PTSD), this provides a rational intervention for an established mechanism of symptom development.

Informed by the British Psychological Guidance [33] for management of those recovering from COVID-19 and PPI input, the sessions will cover the following topics:

1: Introduction, expectations, motivation and goal setting

2: Fear avoidance and pacing

3: Management of emotions (perceived stigma, mood/unhelpful thoughts)

4: Recovery and sleep, sleep management strategies

5: Understanding stress and anxiety and management strategies

6: Managing setbacks and long-term behaviour change and future goals

Each session will include a facilitated group discussion, with interactive components. To prevent on-line fatigue, sessions will last up to 60 minutes. Participant resources will include a professionally produced workbook highlighting key topics and providing the opportunity for reflection and learning between sessions. This will be supplemented with pre-recorded on-line short video content available to participants via the study online video platform.

Participants will be provided with a participant workbook which includes general information about the study, guidance on how the study intervention will be delivered, and instructions on how to exercise safely, space for recording booked exercise/support sessions and completed exercise and worksheets to supplement the psychological support sessions.

REGAIN practitioner training: We have extensive experience of training and upskilling staff to deliver rehabilitation and behaviour change programmes (SPHERe, I-WOTCH, PULSE) [34, 35]. We will adapt and combine experiences from these trials to deliver a physical and psychological intervention for COVID-19 survivors.

Safety: All supervised sessions will be led by staff experienced in assessment, prescription and delivery of exercise for multi-morbid clinical populations. Pre-exercise session online poll questions will be completed by participants to capture any adverse events since the previous supervised session. Staff will monitor for adverse events during the sessions and will ask if anyone has anything to report at the end of the session to remain online after the session or to call/email any concerns.

If a participant does not attend two consecutive intervention appointments, a REGAIN practitioner will attempt to contact them via telephone or email in order to ascertain their welfare.

Exercise carries a very small risk of complications. All participants will be assessed for any underlying health conditions or severe complications related to COVID-19. Participants will be excluded from the study at the eligibility stage where exercise is clearly contraindicated, as assessed by a clinical member of the research team. A further assessment will be undertaken by the REGAIN practitioner, through discussion with the patient about their current health, at the time of the initial online intervention assessment. Any additionally identified contra-indications at this stage will result in withdrawal from the study intervention.

All participants will be advised to have another person nearby for the initial exercise sessions. We will encourage this wherever possible.

The REGAIN practitioner will advise on an exercise regime appropriate for each participant's ability. Participants may contact the REGAIN practitioners or the study team via email or telephone with any queries regarding their exercise regime, progress, changes to health or any adverse events or alternatively they may raise these queries before or after a class.

Emergency procedure: Participants will undertake live exercise and support sessions in discrete groups of up to 10 people. In advance of each session, the practitioner will have access to contact details for each participant. During the sessions, the practitioner will be able to see each participant individually on a large screen. In the event of an emergency, the practitioner will alert the designated 'co-pilot' for the session who will be able to communicate directly with the participant in question (via the live call or telephone) outside of the group, and alert the emergency services if required.

Intervention/control delivery: The REGAIN study will be delivered nationwide from a single central study 'hub'. The UHCW community exercise rehabilitation centre delivers NHS cardiac, pulmonary, vascular, heart failure, cancer and other long-term condition rehabilitation services for Coventry and surrounding areas. Practitioners trained in the REGAIN intervention will be able to deliver the programme to groups of participants anywhere in the country using pre-recorded and live exercise and psychological support sessions.

2.9.4 **Compliance**

Compliance with REGAIN intervention: Attendance at live online exercise sessions and the psychological support sessions will be logged by the online platform for each participant every week. Participants will be identified using their email address. Data will also be recorded detailing the number of times an individual has clicked onto an online video and the amount of time they have spent viewing each video. The completion of intervention (individual assessment, online live exercise sessions, psychological support sessions and online pre-recorded exercise sessions) and control sessions will be recorded as one measure of compliance.

Definition of compliance with intervention: The impact of compliance on outcomes will be assessed using a CACE (compliers average causal effect) analysis. A detailed statistical analysis plan will be written and approved by the Data Monitoring Committee (DMC) including definitions of full and partial compliance for the intervention group.

Fidelity: The majority of live exercise sessions and psychological support sessions will be recorded to reduce the risk of those delivering the intervention behaving differently when being recorded. The psychological support sessions will be recorded and scored against criteria. From these sessions, a purposively selected subset (10%) of recordings will be analysed across relevant intervention sessions by the REGAIN process evaluation team. This will enable assessment of fidelity, and an understanding of areas and issues that generated discussion.

The control group and intervention group individual practitioner appointments will also be recorded and scored against criteria.

2.10 Concomitant illness and medication

2.10.1 Concomitant illness

At the start of the study, potential participants will be screened during their eligibility assessment for any concomitant illnesses. If the illness influences the potential participant's eligibility to continue in the study (e.g. serious mental health problems that preclude participation in a group intervention) the investigator will be informed and they will not be eligible to participate.

2.10.2 Concomitant medication

Participants will be asked to record any medications they are taking, for COVID related problems, at each follow-up time point (three, six and 12 months).

2.11 Co-enrolment into other trials

Co-enrolment of REGAIN participants onto other interventional studies will be considered where there is no conflict with the REGAIN study objectives. A list of appropriate and agreed studies will be produced at a national level to guide co-enrolment. In addition, the CI will review the protocols for other studies and will consider co-enrolment in conjunction with the Trial Management Committee where appropriate.

2.12 End of study

The study will end when all participants have completed their 12-month follow-up. As part of the process evaluation, n=25 participants in the control arm and n=25 from the intervention arm will be interviewed **after** their three-month follow-up.

The study will be stopped prematurely if:

- Mandated by the Ethics Committee
- Following recommendations from the Data Monitoring Committee (DMC)
- Funding for the study ceases

The Research Ethics Committee will be notified in writing within 90 days when the study has been concluded or within 15 days if terminated early.

3. METHODS AND ASSESSMENTS

3.1 Schedule of delivery of intervention and data collection

Table 1. Study assessments

	**Pre-randomisation**		**Post-randomisation**			
**Online assessment**	**1**	**2**	**3**	**4**	**5**	**6**
**Assessment time point**	**Screening**	**Enrolment/ randomise (Baseline)**	**Intervention Delivery**	**3m (± 2w)**	**6 m (± 1 m)**	**12 m (± 1 m)**
Invitation letter and flyer posted	✓					
Initial Eligibility Assessed	✓					
Concomitant Illnesses		✓				
Eligibility check* (telephone)		✓				
Informed consent		✓				
Patient Demographics		✓				
Medication Use				✓	✓	✓
PROMIS® 29+2 Profile v2.1 (PROPr)		✓		✓	✓	✓
PROMIS dyspnoea		✓		✓	✓	✓
PROMIS Neuro-QoL		✓		✓	✓	✓
EQ-5D-5L		✓		✓	✓	✓
IPAQ-SF		✓		✓	✓	✓
IES-R		✓		✓	✓	✓
HADS		✓		✓	✓	✓
Work status		✓		✓	✓	✓
Intervention			✓			
Adverse events			✓			
Overall health		✓		✓	✓	✓
Death						✓

	Pre-randomisation		Post-randomisation			
**Online assessment**	**1**	**2**	**3**	**4**	**5**	**6**
**Assessment time point**	**Screening**	**Enrolment/ randomise (Baseline)**	**Intervention Delivery**	**3m (± 2w)**	**6 m (± 1 m)**	**12 m (± 1 m)**
Health and Social Care resource use				✓	✓	✓
Semi-structured interviews (Process evaluation)				✓		

* Eligibility check will be performed in person over the telephone by a clinical member of the REGAIN team. All other assessments and information will be completed by the participant online.

3.2 Long term follow-up assessments

Long-term follow-up: Consent will be sought from participants to keep their personal data. Consent will also be taken to request a copy of the participant's medical record from their GP, should they not respond to the 12-month follow-up questionnaire, or have died at the end of the study follow-up period. This will provide information on GP consultations and include copies of any hospital discharge letters allowing us to accurately cost in-patient care costs. Mortality data will be gathered from GP records at 12 months.

3.3 Symptoms sub-study

Study sites (Pic sites and UHCW) will be asked to send information to WCTU on recorded duration of hospital stay and type of ventilation received. These data will be sent to WCTU in a pseudonymised format with the participant identified using the screening ID number.

These data will not be collected from those patients entering the study via the self-referral route or those invited by NHS Digital.

3.4 Embedded process evaluation

Semi-structured interviews with participants: Information about interviews will be provided to all participants during study recruitment. Participants will be asked to consent (or not) to being contacted around three months after they have entered the study to share their views and experiences of the intervention or control. Participant interviews will be completed by phone or video call by a qualitative Research Fellow from WCTU or appropriately trained member of the REGAIN team. Online consent will be taken prior to the interview taking place.

Pilot study

Interviews with up to five people in each arm recruited to the internal pilot to check intervention acceptability, and identify obstacles or facilitators to participation, uptake and completion. We will use this internal pilot to optimise recruitment and retention by identifying challenges, and solutions which will be discussed with our patient partners. The model used for interviews in the pilot study will differ from the main study in that participants will be interviewed within three months of randomisation rather than after three months.

Main Study
Intervention and control participants will be interviewed to investigate their experiences, contextualise quantitative findings, and explore factors that helped or hindered participation, thus informing interpretation and wider implementation. Interviews will take place after the three month follow-up outcome data collection, so that the interview itself does not introduce bias to the analysis. A purposive sample of up to n=25 intervention and n=25 control participants will be interviewed at three months post randomisation to ensure a diverse range of perspectives are included. Our sample size of up to 25 per group follows guidance [36] indicating that while code saturation ('when researchers have *heard it all*') was reached at nine interviews, 16 to 24 interviews were needed to reach meaningful saturation ('*to understand it all*'). The interviews will use a topic guide that will include participant experiences of COVID-19, and any obstacles or enablers to participation, adherence, and recovery. We will explore what components were used/dropped/never used, and views on the guided home exercise content. The interviews will last about one hour and be recorded.

We will aim to include up to three participants per arm who do not speak English in our interviews. They will be interviewed by staff who have interviewing skills and relevant language skills. They will be interviewed in their first language; this will be transcribed verbatim. The transcript will then be translated into English, then back translated and the back translation compared to the original transcript. This approach will be informed by recent work in this area [37-42].

Practitioner interviews: At the end of the study, all consenting REGAIN practitioners will be interviewed (~n=5) about their experiences of delivering the interventions/best usual care, what worked well, what helped, and what was challenging. These interviews will last up to one hour, be recorded, and piloted during the internal pilot. As the REGAIN trial will be delivered from a single central location, all practitioners involved in study delivery will be interviewed.

4. ADVERSE EVENT MANAGEMENT

4.1 Definitions

4.1.1 Adverse Events (AE)

An Adverse Event (AE) is defined as any untoward medical occurrence involving a participant, which does not necessarily have a causal relationship with the intervention or study.

4.1.2 Serious Adverse Events (SAEs)

A SAE is any untoward medical occurrence that fulfils one or more of the following criteria:

- Results in death
- Is immediately life-threatening
- Requires hospitalisation or prolongation of existing hospitalisation

- Results in persistent or significant disability or incapacity
- Is a congenital abnormality or birth defect
- Intervention is required to prevent one of the above or is an important medical condition

4.2 Recording Adverse Events and Reporting Serious Adverse Events

4.2.1 Recording and reporting period

Intervention group: All AEs and SAEs that occur during or within 24 hours of a REGAIN live online session or an on-demand exercise session (supervised or unsupervised) should be recorded on the AE log by the exercise practitioner.

Usual care group: The usual care group will be asked about anything that might constitute a Serious Adverse Event at the time of their three-month follow-up. It will not be possible to collect a comparison dataset for the usual care group within this period without contaminating the control intervention. This is a pragmatic study and the participants will not be contacted during the intervention period, unlike the intervention group. It is important not to contact the usual care group more than is necessary so as not to introduce bias. We anticipate a low risk of adverse events arising from best practice usual care i.e. an NHS website and a single session of advice.

4.2.2 Recording Adverse Events

Participants in the intervention group will have the opportunity to indicate whether or not they have experienced AEs by completing pre- exercise session poll questions. Responses indicating the participant has potentially experienced an adverse event will be evaluated by the practitioner and the participant contacted to confirm the details. Practitioners should also monitor for any information volunteered by a participant at any time during a live exercise session and will ask if anyone has anything to report at the end of the session to remain online after the session or to call/email any concerns. Participants in the intervention group will also have contact details (generic email address and phone number) for the study team and practitioners. Whilst participants will not be actively encouraged to report AEs via this route, they may seek advice and help from the team which may result in AEs being disclosed/discussed. Any AEs will be recorded on the AE log unless they fulfil the criteria for a 'Serious Adverse Event' in which case they will be reported to WCTU via the SAE form (see section 4.2.3 below). The following will not be classed as AEs and will therefore not be recorded on the AE log:

- Participants' normal COVID-19 symptoms.
- Normal post exercise symptoms e.g. moderate levels of shortness of breath, tiredness, muscle and/or joint soreness/stiffness including delayed onset of muscle soreness (DOMS).
- Exacerbation of pre-existing musculoskeletal conditions (e.g. osteoarthritis) as long as not more than 72hrs in duration.

4.2.3 Reporting Serious Adverse Events

All AEs should be assessed by the research practitioner to determine if they meet the criteria to be reported as a 'serious adverse event' as defined in section 4.1.2. If any of the adverse events meet this criteria, they will be reported to WCTU by emailing WCTUQA@warwick.ac.uk using the Serious Adverse Event form within 24 hours of becoming aware of it. If the 3 month questionnaire for a

control group or REGAIN intervention group participant indicates events that may fulfil an SAE, then they should be contacted for further information and an SAE form completed if applicable.

A clinical assessment of whether the event has a causal relationship to the intervention should be made and recorded on the form by the practitioner. All SAEs should be reported irrespective of their relationship to the intervention unless they are exempt from reporting (see section 4.2.3.1).

For each **SAE** the following information will be collected:

- full details in medical terms and case description using CTCAE V5.0
- event duration (start and end dates, if applicable)
- action taken
- outcome
- seriousness criteria
- causality (relatedness to intervention), in the opinion of the practitioner

4.2.3.1 SAEs that are exempt from reporting

The following events that would usually fulfil the criteria for 'serious' do **not** need to be reported as per section 4.2.3:

- Treatment, which was elective or pre-planned, for a pre-existing condition, not associated with any deterioration in condition
- General care, not associated with any deterioration in condition
- Planned hospital admissions.

4.2.4 Determination of causality and expectedness for SAEs

Two independent causality assessments will be performed (i.e. relationship to study intervention). The practitioner will submit an assessment of their clinical opinion on causality upon submission of the SAE report using the classifications in SAE table 2 below. The CI will then do a separate causality assessment on reported events on behalf of the sponsor. These two assessments should be independent of each other.

If either party suspect there is a possibility that the event is related to the intervention then a delegate on behalf of the Sponsor will assess whether or not this is expected using the information in 4.2.4.1 below. For any related and unexpected serious adverse events, WCTU will report this to the REC within 15 days of receipt.

4.2.4.1 Expected Serious Adverse Events

Due to the limited knowledge of the long-term health problems in COVID-19 survivors, there are no Serious Adverse Events that would be expected in exercise intervention for the population included in this study.

Table 2. SAE Causal relationship

Relationship to study medication	Description
Unrelated	There is no evidence of any causal relationship
Unlikely to be related	There is little evidence to suggest there is a causal relationship (e.g. the event did not occur within a reasonable time after administration of the study intervention). There is another reasonable explanation for the event (e.g. the patient's clinical condition, other concomitant treatment).
Possible relationship	There is some evidence to suggest a causal relationship (e.g. because the event occurs within a reasonable time after administration of the study intervention). However, the influence of other factors may have contributed to the event (e.g. the patient's clinical condition, other concomitant treatments).
Probable relationship	There is evidence to suggest a causal relationship and the influence of other factors is unlikely.
Definitely related	There is clear evidence to suggest a causal relationship and other possible contributing factors can be ruled out.

4.2.5 **Follow-up of reported SAEs**

Practitioners will monitor for changes to unresolved SAEs via intervention poll responses or through close contact with the participant. If a practitioner becomes aware of any change of condition or other follow-up information it should be emailed to WCTUQA@warwick.ac.uk on the Serious Adverse Event Follow-Up form as soon as it is available or at least within 24 hours of the information becoming available. Events will be followed up until the event has resolved or a final outcome has been reached where possible.

4.3 Responsibilities

Practitioners:

Checking for AEs when participants attend for exercise session or via pre -exercise poll responses:

1. Using clinical judgement in assigning seriousness and causality
2. Ensuring that all SAEs are recorded and reported to the Sponsor via Warwick QA within 24 hours of becoming aware of the event and provide further follow-up

information as soon as available. Ensuring that reported SAEs are chased with WCTU if a record of receipt is not received within 2 working days of initial reporting.

Chief Investigator (CI) / delegate or independent clinical reviewer:

1. Clinical oversight of the safety of patients participating in the study, including an ongoing review of the risk / benefit.
2. Using clinical judgement in assigning causality
3. Immediate review of all related and unexpected SAEs
4. Review of specific SAEs in accordance with the study risk assessment and protocol as detailed in the Trial Monitoring Plan.
5. Production and submission of annual reports to the relevant REC.

Sponsor or delegate:

1. Central data collection and verification of SAEs, according to the study protocol.
2. Expectedness assessment of related SAEs
3. Reporting safety information to the CI, delegate or independent clinical reviewer for the ongoing assessment of the risk / benefit according to the Trial Monitoring Plan.
4. Reporting safety information to the independent oversight committees identified for the study (Data Monitoring Committee (DMC) and / or Trial Steering Committee (TSC)) according to the Trial Monitoring Plan.
5. Expedited reporting of related and unexpected SAEs to the REC within required timelines.
6. Notifying Investigators of related and unexpected SAEs that occur within the study.

Trial Steering Committee (TSC):

In accordance with the Trial Terms of Reference for the TSC, periodically reviewing safety data and liaising with the DMC regarding safety issues.

Data Monitoring Committee (DMC):

In accordance with the Trial Terms of Reference for the DMC, periodically reviewing unblinded overall safety data to determine patterns and trends of events, or to identify safety issues, which would not be apparent on an individual case basis.

4.4 Notification of deaths

All deaths, when they are identified, will be reported to the sponsor by the REGAIN practitioner, overseen by the CI, irrespective of whether the death is related to disease progression, the intervention, or an unrelated event.

Staff at the UHCW community exercise rehabilitation centre and WCTU may become aware of deaths that occur during the study however the majority of deaths will be identified by accessing GP records for those non-responders at 12 months post-randomisation.

4.5 Reporting urgent safety measures

If any urgent safety measures are taken the CI/Sponsor shall immediately and in any event no later than 3 days from the date the measures are taken, give written notice to the relevant REC of the measures taken and the circumstances giving rise to those measures.

5. DATA MANAGEMENT

Personal data collected during the study will be handled and stored in accordance with the General Data Protection Regulation and Data Protection Act 2018.

Personal identifying information will be collected via the online database and stored electronically at WCTU. Subsequently, a letter will be sent to the participants GP at baseline and follow-up time points if potential case level mental health disorders are identified. Participant details will be stored and accessed by staff at WCTU and UHCW via the online database to: confirm eligibility and consent; allow postage of participant manuals; contact participants during the study; allow delivery of intervention and control procedure; contact for qualitative interviews; and to request a copy of the participant's medical record from their GP. Handling of personal and confidential data will be clearly documented in the participant information sheet and consent obtained.

REGAIN practitioners at UHCW will also keep paper records of participant contact details and medical health information for those participants randomised to the REGAIN intervention. This is required for study delivery to ensure participants are exercising at the appropriate level and to be used if a medical emergency occurs. These paper records will be stored securely in locked filing cabinets only accessible to study staff. These records will not be passed onto WCTU.

Disclosure of confidential information will only be considered if there is an issue which may jeopardise the safety of the participant or another person, according to Warwick SOPs (Warwick SOP 15 part 1) and the UK regulatory framework. There is no reason to expect this situation to occur in this study more than any other.

5.1 Data collection and management

The CRFs and questionnaires will be developed by the Trial Manager in consultation with the CI, Statistician, Health Economist and other relevant members of the study team to collect all required study data.

All data will be entered directly by participants, UHCW staff, REGAIN practitioners or WCTU study team members onto a secure online study database hosted by WCTU as outlined in the data management plan and in accordance with the Warwick SOPs. Data entered onto the online study database will be source data. This will be stored safely and securely. On all study-specific documents, other than the completed consent form, the participant will be referred to by the study participant number, not by name.

Various methods will be used to chase missing data including phone, text and email. Participants will receive a reminder to complete the online questionnaires at each study time point. If a participant has not completed a study questionnaire following the reminder, the REGAIN study team will contact the participant to encourage them to complete the questionnaire online, and to

provide support where required. If data still remain missing following this chase, the REGAIN study team will contact the participant to attempt to collect the outcome measurements with priority on the core measures (PROPr and EQ5DL). Where necessary a bilingual researcher will assist with the collection of missing data from participants who are non-English speakers. The procedures for managing this will be outlined in the data management plan and appropriate consent will be sought to contact participants.

Data will still be collected for participants who discontinue or deviate from the intervention protocol, unless they withdraw their consent (section 2.9.2). For any participants who do not respond to the 12 month follow-up questionnaire or who are known to have died, WCTU will request a copy of the participant's medical record from their GP. Identifiable data will be deleted 12 months after study completion (last follow-up for last participant).

5.2 Database

The database will be developed by the Programming Team at WCTU and all specifications (i.e. database variables, validation checks, screens) will be agreed between the programmer and appropriate study staff.

5.3 Online video platform

An external online video platform (Beamfeelgood) will be used for the REGAIN study interventions. This platform will enable live streaming of intervention sessions and the hosting of on-demand, pre-recorded content. A Data Protection Impact Assessment has been completed and approved by UHCW NHS Trust in order to identify and minimise associated risks. The online platform is GDPR compliant and Organisation for Review of Care and Health Apps (ORCHA) accredited. Any data that is stored, including the participant's email address, will be encrypted in accordance with NHS Digital guidance and storage will be NHS cloud compliant, conforming to ISO 9001/27001/27017/27018 standards and the G-Cloud (UK Government) standard.

Private groups will be created on the online platform and administrative access to these given only to UHCW/UoW approved staff. Admin users will authorise participant's access to the private groups and participants will be asked for their consent to share utilisation metrics with the group admin. Participants may choose to use a nickname on the online platform to remain anonymous to other members.

This online video platform will collect and store data on participants attendance at classes, the amount of time participants have spent watching on demand videos and answers to any pre- or post-exercise session online poll questions. This information will be stored against the participants name and email address on google data studio until the end of the study. The REGAIN team (WCTU and UHCW) will be given access to this data as required to monitor attendance, safety and for analysis of compliance.

5.4 One-to-one consultation platform

All one-to-one consultations between the REGAIN practitioner and a study participant will take place on an online video platform (MS Teams or similar) supported and approved by UHCW Trust. This will include the best practice usual care advice consultation in the control group and the individual assessment component of the REGAIN intervention.

5.5 Data storage

All essential documentation and study records will be stored at WCTU in conformance with the applicable regulatory requirements and access to stored information (electronic and paper) will be restricted to authorised personnel. Electronic data will be stored on password protected university computers. All data will be stored in a designated storage facility within the WCTU and UHCW.

5.6 Data access and quality assurance

The majority of data will be received directly from participants who will enter their data into the online study database. Following the completion of the initial screen form (which includes an initial eligibility check and provision of contact details) participants will be contacted using the contact details that they have provided to confirm eligibility. Participants will complete an online consent form. After the collection of the baseline demographic data for each participant and following randomisation all data will be pseudonymised. Confidentiality will be strictly maintained and names, addresses or personal identifiable information will not be disclosed to anyone other than the staff involved in running the study. All electronic participant-identifiable information will be held on a secure, password-protected database accessible only to essential personnel. Paper forms will be held in secure, locked filing cabinets within a restricted area of WCTU. Participants will be identified by a participant number only on the paper forms. Direct access to source data (online study database) will be available for study-related monitoring or audit by UHCW or WCTU for internal audit or regulatory authorities. The PI must arrange for retention of study records on site in accordance with GCP and local Trust's policies.

Direct access to source data/documents will be required for study-related monitoring. For quality assurance, the data and results will be statistically checked. A full data management plan will be produced by the study manager and statistician to outline the data monitoring checks required.

5.7 Data Shared with Third Parties

Requests for data sharing will be managed in accordance with University of Warwick SOP 15 Part 3. The datasets generated during and/or analysed during the current study are/will be available upon request after publication of the main study results. The publication of a study protocol, study results and study data will comply with the NIHR standard terms and will follow Warwick SOP 22: Publication & Dissemination.

5.8 Archiving

Study documentation and data will be archived for at least ten years after completion of the study. Study documentation and data held by NHS PIC sites will be stored in line with their local trust policy.

6. STATISTICAL ANALYSIS

6.1 Power and sample size

We have no data on which to base a sample size estimation. There are no normative data for the PROPr quality of life scores in this population and no external indication of what might be a worthwhile benefit from the intervention on quality of life outcomes for this population. American values for the general population in the USA are a mean score of 50 (1-100 scale) with an SD of 10. Whilst not our preferred practice, we will use the approach of looking for a small to moderate standardised mean effect size of 0.3. Allowing for a clustering effect in the intervention arm, we assume that a group size will consist of a maximum of eight patients. Then assuming an intra cluster coefficient of 0.01, 90% power and type I error rate of 5%, with a 10% loss to follow-up, we require 535 participants. This equates to 272 participants in the intervention arm across up to 34 groups and 263 patients in the control arm (control:intervention = 1:1.03), using computations recommended by Moerbeek [43].

To account for a higher than expected drop out rate the study will over recruit until sufficient participants meet the primary end point. Recruitment will be within approximately 10% of the original target and end of recruitment will be determined by the trial statistician in consultation with the trial management team/TMG as appropriate.

6.2 Statistical analysis of efficacy and harms

6.2.1 Statistics and data analysis

A detailed statistical analysis plan will be written and approved by the Data Monitoring Committee (DMC).

Data will be summarised and reported as per CONSORT, using intention-to-treat analyses.

For the primary outcome measures, treatment effects (with 95% Confidence Intervals) will be estimated using hierarchical linear regression models, both unadjusted and adjusted (for stratification variables and important patient-level covariates) will be presented. We will estimate and adjust for site effects as a random variable in the model. Other secondary outcomes which are continuous will be analysed in a similar way. Secondary outcomes which are categorical will be analysed using logistic regression models. We will assess compliance using Compliers Average Causal Effect (CACE) analysis. In the case of missing outcome data, we will compute sensitivity analyses using imputation techniques to examine the impact of missingness.

There are no formal interim analyses for this study.

6.2.2 Planned recruitment rate

A minimum recruitment rate of 67 participants per month will be required, based on a recruitment target of 535 participants over 8 months. Patients will be identified from roughly 20 sites (UHCW NHS Trust and PIC sites) in addition to patients identified via self-referrals. The target recruitment

rate for the study has been discussed with and agreed by the Trial Management Group (TMG). We are unable to estimate the numbers of self-referrals to the study at this stage. We will have data on proportion of self-referrals after the internal pilot study.

6.3 Subgroup analyses

Pre-specified, exploratory sub-group analysis will include age, need for critical care support, depression, anxiety, PTSD and ethnicity. The sub-group effects will be assessed using regression modelling with the interaction term of sub-group and treatment. As the sub-groups are not powered, the results will be reported using 95% confidence intervals.

6.4 Health economic evaluation

A prospectively planned economic evaluation will be conducted from a NHS and personal social services perspective, according to the recommendations of the NICE reference case [44].

The costs associated with implementing the intervention and control will be captured by the trial team. Additionally, participants' health service contacts will be recorded at three, six and 12 months, this includes: healthcare, local authority-provided day care and NHS residential services. Time lost from work (paid/unpaid) and patient-borne health costs (e.g. wheelchair by type, home adaptations, feeding aids, walking aids, home-help, support from relatives) will also be recorded to examine a broader social perspective. Healthcare resource use will be costed using most recently available published national reference costs, reflated to a common year [45, 46].

Generic health-related quality-of-life will be assessed at baseline, three, six, and 12 months using the EQ-5D-5L questionnaire. EQ-5D-5L scores will be converted to health status scores using the UK value set recommended by NICE guidance at the time of analysis [47]. Using the trapezoidal rule, the area-under-the-curve of health status scores will be calculated, providing patient-level QALY estimates. Reflecting the one year timeframe, costs and QALYs will be undiscounted.

Mechanisms of missingness of data will be explored and multiple imputation methods will be applied where appropriate to impute missing data. Imputation sets will be used in bivariate analysis of costs and QALYs, using the STATA MI framework. Within-study (12 month) incremental cost per QALY estimates and confidence intervals will be estimated [48-51]. Findings will be analysed and visualised in the cost-effectiveness plane, as cost-effectiveness acceptability curves, net monetary benefit and value of information analysis. At the time of writing no method is available to analyse one-arm clustering within a bivariate regression framework. Ignoring clustering may result in some over-precision of findings if the clustering effect is significant, although have limited scope to systematically bias findings. The importance of clustering will be explored within a hierarchical univariate sensitivity analysis of net monetary benefit (NMB) at varying thresholds of willingness to pay. If incremental costs and benefits are non-convergent within the study follow-up then extrapolated modelling will be considered.

6.5 Qualitative data analysis

The semi-structured interviews with ~n=25 intervention group, ~n=25 control and ~n=5 practitioner's will be recorded, subject to the permission of each participant/practitioner,

pseudonymised, and transcribed verbatim. Framework analysis will be used to analyse the data [52]. This will involve:

- Data familiarisation: listening to digital recordings, reading transcripts, and re-reading field notes;
- Identifying a thematic framework: key issues and themes identified and an index of codes is developed;
- Indexing: this index is applied to all data;
- Charting: a summary of each passage of text is transferred into a chart to allow more overall and abstract consideration of index codes across the data set and by each individual;
- Mapping and interpretation: understanding the meaning of key themes, dimensions and broad overall picture of the data and identifying and understanding the typical associations between themes and dimensions. We will remain vigilant for any new themes emerging from the data as we progress. The computer package NVivo 12 will be used to organise the data.

The charting process provides an opportunity to code data from numerous perspectives. The computer package NVivo 11 will be used to organise the analysis.

The findings of the qualitative work will be reported as a separate chapter in the final report but will also be incorporated in the discussion to bring together a synthesis of all the results, thus helping to explore and explain the overall 'value' of the interventions. Quantitative and qualitative data will be integrated using a mixed methods matrix' where quantitative responses can be compared to interview data and recorded on a matrix. This is particularly useful to reveal gaps between quantitative and qualitative insights.

From the intervention delivery recordings (initial practitioner assessment, the exercise familiarisation session and the psychological support sessions) and control (1:1 session) recordings, a purposively selected subset (10%) of recordings will be analysed, with a checklist to assess fidelity and using the qualitative approach detailed above to help understand which areas generated discussion and what issues were discussed. Intervention fidelity will be assessed using the tenets highlighted by Mars et al.

7. STUDY ORGANISATION AND OVERSIGHT

7.1 Sponsorship and governance arrangements

University Hospitals Coventry and Warwickshire NHS Trust will act as Sponsor for the study and undertake the responsibilities as defined by the UK Policy Framework For Health and Social Care Research and Good Clinical Practice guidelines. An authorised representative of the Sponsor has approved the final version of this protocol with respect to the study design, conduct, data analysis and interpretation and plans for publication and dissemination of results.

Study management will be undertaken at Warwick Clinical Trials Unit, the University of Warwick. A sub-contract agreement is in place between UHCW and WCTU who will provide full research management services. This will specify whose SOPs will be adhered to for each aspect of the study.

PIC agreements will also be in place between the Sponsor and each research site, with clear delegation of roles and responsibilities.

7.2 Ethical approval

All ethical approvals will be sought using the Integrated Research Application System. The study will be conducted in accordance with relevant regulations and guidelines. Before enrolling people into the study, each study site must ensure that the local conduct of the study has the agreement of the relevant NHS Trust Research & Development (R&D) department. Sites will not be permitted to send out invitation letters for the study until written confirmation of R&D agreement is received by the co-ordinating team. Substantial protocol amendments (e.g. changes to eligibility criteria) will be communicated by the study team to relevant parties i.e. investigators, participants, NHS Trusts and study registries once approved. Annual reports will be submitted to the REC within 30 days of the anniversary date on which the favourable opinion was given, and annually until the study is declared ended. The REC and sponsor will be notified of the end of the study (whether the study ends at the planned time or prematurely). The CI will submit a final report to the required authorities with the results, including any publications, within one year ending the study.

7.3 Peer Review

This study was peer reviewed by NIHR COVID-19 Recovery and Learning cross programme commissioning board.

7.4 Study Registration

The study will be registered on the International Standard Randomised Controlled Trial Number (ISRCTN) Register.

7.5 Notification of serious breaches to GCP and/or study protocol

A "serious breach" is a breach which is likely to effect to a significant degree –

(a) the safety or physical or mental integrity of the subjects of the study; or
(b) the scientific value of the study

The sponsor will be notified immediately of any case where the above definition applies during the study conduct phase and will notify the licensing authority in writing of any serious breach of

(a) the conditions and principles of GCP in connection with that study; or
(b) the protocol relating to that study, as amended from time to time, within 7 days of becoming aware of that breach

7.6 Indemnity

NHS indemnity covers NHS staff, medical academic staff with honorary contracts, and those conducting the study. NHS bodies carry this risk themselves or spread it through the Clinical Negligence Scheme for Trusts, which provides unlimited cover for this risk. The University of Warwick has Public Liability and Clinical Trials insurance cover in place to cover its own legal

liabilities arising from the Study, including for any harm caused to participants by the design of the research protocol.

7.7 Study timetable and milestones

	Month	Recruitment
Set-up	0* – 1	n/a
Intervention development phase	1 – 2	n/a
Pilot study	2 – 3	35
Main recruitment	2 – 15	500
Primary Outcome	5 – 18	n/a
Follow-up	5 – 22	n/a
Process Evaluation	3 – 16	n/a
Analysis	14 – 17 / 23 – 24	n/a

**Month 0 estimated to commence 01st September 2020.*

7.8 Administration

The study co-ordination will be based at WMS/WCTU, University of Warwick.

7.9 Trial Management Group (TMG)

The Trial Management Group, consisting of the project staff and co-investigators involved in the day-to-day running of the study, will meet regularly throughout the project. Significant issues arising from management meetings will be referred to the Trial Steering Committee or Investigators, as appropriate.

The full remit and responsibilities of the TMG will be documented in the Charter which will be signed by all members.

7.10 Trial Steering Committee (TSC)

The study will be guided by a group of respected and experienced personnel and trial methodologists as well as at least one 'lay' representative. The TSC will have an independent chair-person. Meetings will be held at regular intervals determined by need but not less than once a year. Routine business is conducted by email, post or teleconferencing.

The Steering Committee, in the development of this protocol and throughout the study will take responsibility for:

- Major decisions such as a need to change the protocol for any reason
- Monitoring and supervising the progress of the study
- Reviewing relevant information from other sources

- Considering recommendations from the DMC
- Informing and advising on all aspects of the study

The membership of the TSC is shown on page 5.

The full remit and responsibilities of the TSC will be documented in the Committee Charter which will be signed by all members.

7.11 Data Monitoring Committee (DMC)

The DMC will consist of independent experts with relevant clinical research, and statistical experience. The DMC meeting frequency will be guided by the DMC chair, but will be suggested to be three months into the recruitment phase and regularly thereafter, as directed by the DMC chair. Confidential reports containing recruitment, protocol compliance, safety data and interim assessments of outcomes will be reviewed by the DMC. The DMC will advise the TSC as to whether there is evidence or reason why the study should be amended or terminated. The membership of the DMC will be approved and appointed by the NIHR.

DMC meetings may also be attended by the CI and Trial Manager (for non-confidential parts of the meeting) and the trial statistician.

The full remit and responsibilities of the DMC will be documented in the Committee Charter which will be signed by all members.

7.12 Essential Documentation

A Trial Master File will be set up in accordance to Warwick SOP 11 - 'Essential Documentation' and held securely at WCTU. Investigator Site Files will be prepared electronically and the content for the investigator site files will be uploaded to the study website (https://warwick.ac.uk/regain) for sites to download. UHCW will hold and maintain a Sponsor oversight file.

7.13 Financial Support

The study has been funded by a grant from NIHR Recovery and Learning programme further to a commissioned call (NIHR: 132046).

8. MONITORING, AUDIT AND INSPECTION

The study will be monitored by the Research and Development Department at UHCW as representatives of the lead Sponsor, and by the Quality Assurance team at WCTU as representatives of the study coordinating centre and academic lead, to ensure that the study is being conducted as per protocol, adhering to Research Governance and GCP. The approach to, and extent of, monitoring will be specified in a study monitoring plan determined by the risk assessment undertaken prior to the start of the study. A Trial Monitoring Plan will be developed and agreed by the TMG and TSC based on the study risk assessment, including on site monitoring if applicable.

Processes to be considered in the monitoring plan will include participant enrolment, consent, eligibility, and allocation to study groups; adherence to study interventions and policies to protect participants, including reporting of harm and completeness, accuracy, and timeliness of data collection. The plan will be available from the study coordination centre and will also be lodged with the sponsor. Whilst the monitors work in the same institution as the study team (WCTU), they will act independently in this role.

If the UHCW community exercise rehabilitation centre are persistently late in reporting SAEs, or there is evidence that the study protocols and procedures are not being adhered to (as assessed by the CI or the TMG) an on-site monitoring visit may be triggered where this is possible. The sponsor will ensure investigator(s) and/or institutions will permit study-related monitoring, audits and REC review, providing direct access to source data/documents as required. Monitoring will be performed by exploring the study dataset or performing central monitoring procedures and/or site visits, as defined in the study monitoring plan. Staff at WCTU and UHCW community exercise rehabilitation centre are obliged to assist the sponsor in monitoring the study. These may include hosting site visits, providing information for remote monitoring, or putting procedures in place to monitor the study internally.

9. PATIENT AND PUBLIC INVOLVEMENT (PPI)

We are setting up a reference group of COVID-19 survivors (>3/12 post-discharge) from the recently established COVID-19 follow-up clinic at UHCW. The PPI group will advise on intervention content, study processes and outcomes. As part of setting up this group, we will identify two further COVID-19 survivors to join the Trial Management Group and Steering Committee.

Our lay co-applicants will sit on the trial management group (TMG), initially meeting monthly and subsequently quarterly, and will have a pivotal role in steering the conduct of the study. They will review the ethics application to ensure that study documentation e.g. participant information sheet, is user appropriate. They will be given the opportunity to engage in study publicity and the dissemination of findings through appropriate channels i.e. social media, lay conferences, public engagement events, service provider events, newsletter articles. They will be viewed as members of the research team, with experience and skill that can contribute fully to the successful conduct of the study, and will be asked to be involved in measuring and reporting research impact. A role description and terms of reference for lay co-applicants has been produced in collaboration with our lay partners and the UHCW Patient and Public Research Advisory Group (PRAG). This will ensure that both parties understand the nature and extent of the collaboration, and their expectations of each other.

In addition to reviewing ethics documentation, we will ask our lay partners to work closely with the research team, acting as critical reviewers, in finalising the resources for REGAIN - practitioner manual, the home exercise guidance material, and the control group information. This is essential to ensure creation of feasible, acceptable and participant friendly resources. They will also help develop the interview topic guide and will contribute to the interpretation of qualitative data analysis.

Lay co-apps and partners will be supported by the Chief Investigator, study coordination team, and through the peer support of lay partners on existing clinical trials. Comprehensive training and support will be provided by UHCW NHS Trust R&D department who run regular lay seminars, group

training and social events through the PRAG, with governance from PALS. All activity will be appropriately reimbursed at INVOLVE rates, for which there is adequate provision in the budget. Lay partners will also benefit from training and support from Warwick CTU's existing one-day face-to-face training programme for patient and public partners which was developed in collaboration with a patient partner from another study who suggested the original need for, and content of, the course.

10. DISSEMINATION AND PUBLICATION

We will publish the primary analysis on three months outcomes as soon as possible after these are available to ensure they immediately inform practice. Full results of the study will be prepared by the research team and lay partners and submitted to funders as a final report. Findings will be submitted to peer-reviewed journals and disseminated to the medical and exercise rehabilitation communities. We will publish papers in open-access journals describing the development and refinement of the REGAIN intervention, and the study protocol, as per recommended guidance for transparent reporting, the Consolidated Standards of Reporting Trials (CONSORT) guidelines (www.consort-statement.org), the NIHR standard terms, and Warwick SOP 22: Publication & Dissemination. UHCW NHS Trust as Sponsor will review and approve all publications. We will submit abstracts to national and international conferences.

The REGAIN intervention will be fully manualised and available for public access once the study has completed. If appropriate, we will develop a practitioner training programme to support the implementation of REGAIN.

Our lay partners will help prepare the final report and assist with dissemination of study results. We will produce a lay summary for participants and the hospitals/centres involved. Results will be publicised via the study website and social media. At the end of the study, we will host a joint investigator and participant event to promote key findings. The REGAIN study will be relevant to the NHS thus outputs will follow the usual route into the NHS system and wider society.

HRA guidance on information for participants at the end of a study will be followed:

https://www.hra.nhs.uk/about-us/consultations/closed-consultations/guidance-participant-information-end-study-consultation/

11. REFERENCES

1. National Health Service. After-care needs of inpatients recovering from COVID-19. Available at: https://www.england.nhs.uk/coronavirus/publication/after-care-needs-of-inpatients-recovering-from-covid-19/: NHS England, HM Government: Crown Copyright,; 2020.
2. Lopez M, Bell K, Annaswamy T, et al. COVID-19 Guide for the Rehabilitation Clinician: A Review of Non-Pulmonary Manifestations and Complications. American journal of physical medicine & rehabilitation. 2020.
3. Holmes EA, O'Connor RC, Perry VH, et al. Multidisciplinary research priorities for the COVID-19 pandemic: a call for action for mental health science. Lancet Psychiatry. 2020;7(6):547-60.
4. National Health Service. COVID-19 Hospital Discharge Service Requirements. Available at: https://www.england.nhs.uk/coronavirus/publication/covid-19-hospital-discharge-service-requirements/: NHS England, HM Government: Crown Copyright,; 2020.
5. De Biase S, Cook L, Skelton DA, et al. The COVID-19 Rehabilitation Pandemic. Age and Ageing. 2020.
6. Herridge MS, Tansey CM, Matté A, et al. Functional disability 5 years after acute respiratory distress syndrome. N Engl J Med. 2011;364(14):1293-304.
7. Ñamendys-Silva SA. ECMO for ARDS due to COVID-19. Heart Lung. 2020;49(4):348-9.
8. Hui DS, Joynt GM, Wong KT, et al. Impact of severe acute respiratory syndrome (SARS) on pulmonary function, functional capacity and quality of life in a cohort of survivors. Thorax. 2005;60(5):401.
9. Ngai JC, Ko FW, Ng SS, et al. The long-term impact of severe acute respiratory syndrome on pulmonary function, exercise capacity and health status. Respirology. 2010;15(3):543-50.
10. Wu KK, Chan SK, Ma TM. Posttraumatic stress, anxiety, and depression in survivors of severe acute respiratory syndrome (SARS). J Trauma Stress. 2005;18(1):39-42.
11. Moldofsky H, Patcai J. Chronic widespread musculoskeletal pain, fatigue, depression and disordered sleep in chronic post-SARS syndrome; a case-controlled study. BMC Neurology. 2011;11(1):37.
12. Public Health England. Beyond the data: Understanding the impact of COVID-19 on BAME groups. Available at: https://www.gov.uk/government/publications/covid-19-understanding-the-impact-on-bame-communities: NHS England, HM Government: Crown Copyright,; 2020.
13. McCarthy B, Casey D, Devane D, et al. Pulmonary rehabilitation for chronic obstructive pulmonary disease. Cochrane Database Syst Rev. 2015(2):CD003793.
14. Lau HM, Ng GY, Jones AY, et al. A randomised controlled trial of the effectiveness of an exercise training program in patients recovering from severe acute respiratory syndrome. Aust J Physiother. 2005;51(4):213-9.
15. Liu K, Zhang W, Yang Y, et al. Respiratory rehabilitation in elderly patients with COVID-19: A randomized controlled study. Complement Ther Clin Pract. 2020;39:101166.
16. Whiteford HA, Harris MG, McKeon G, et al. Estimating remission from untreated major depression: a systematic review and meta-analysis. Psychol Med. 2013;43(8):1569-85.
17. RECOVERY trial team. Low-cost dexamethasone reduces death by up to one third in hospitalised patients with severe respiratory complications of COVID-19 Available at: https://www.recoverytrial.net2020 [
18. Moher D, Schulz KF, Altman DG. The CONSORT statement: revised recommendations for improving the quality of reports of parallel-group randomised trials. Lancet. 2001;357(9263):1191-4.

19. Hays RD, Spritzer KL, Schalet BD, et al. PROMIS(®)-29 v2.0 profile physical and mental health summary scores. Qual Life Res. 2018;27(7):1885-91.
20. Rose AJ, Bayliss E, Huang W, et al. Evaluating the PROMIS-29 v2.0 for use among older adults with multiple chronic conditions. Qual Life Res. 2018;27(11):2935-44.
21. Cella D, Choi SW, Condon DM, et al. PROMIS(®) Adult Health Profiles: Efficient Short-Form Measures of Seven Health Domains. Value Health. 2019;22(5):537-44.
22. Herdman M, Gudex C, Lloyd A, et al. Development and preliminary testing of the new five-level version of EQ-5D (EQ-5D-5L). Qual Life Res. 2011;20(10):1727-36.
23. Craig CL, Marshall AL, Sjöström M, et al. International physical activity questionnaire: 12-country reliability and validity. Med Sci Sports Exerc. 2003;35(8):1381-95.
24. Hosey MM, Bienvenu OJ, Dinglas VD, et al. The IES-R remains a core outcome measure for PTSD in critical illness survivorship research. Critical Care. 2019;23(1):362.
25. Needham DM, Sepulveda KA, Dinglas VD, et al. Core Outcome Measures for Clinical Research in Acute Respiratory Failure Survivors. An International Modified Delphi Consensus Study. Am J Respir Crit Care Med. 2017;196(9):1122-30.
26. Weiss D, Marmar C. Assessing psychological trauma and PTSD - The Impact of Event Scale-Revised. New York: The Guilford Press; 1997.
27. Somaini G, Hasler ED, Saxer S, et al. Prevalence of Anxiety and Depression in Pulmonary Hypertension and Changes during Therapy. Respiration. 2016;91(5):359-66.
28. Engel GL. The need for a new medical model: a challenge for biomedicine. Science. 1977;196(4286):129-36.
29. Alonso Y. The biopsychosocial model in medical research: the evolution of the health concept over the last two decades. Patient Educ Couns. 2004;53(2):239-44.
30. Michie S, van Stralen MM, West R. The behaviour change wheel: a new method for characterising and designing behaviour change interventions. Implement Sci. 2011;6:42.
31. Bandura A. Self-efficacy: toward a unifying theory of behavioral change. Psychol Rev. 1977;84(2):191-215.
32. Copeland L, McNamara R, Kelson M, et al. Mechanisms of change within motivational interviewing in relation to health behaviors outcomes: A systematic review. Patient Education and Counseling. 2015;98(4):401-11.
33. British Psychological Society. Guidance for the public on the mental health and wellbeing aspects of coronavirus (COVID-19),. Available at: https://www.bps.org.uk/coronavirus-resources2020.
34. McGregor G, Bruce J, Ennis S, et al. Supervised pulmonary hypertension exercise rehabilitation (SPHERe): study protocol for a multi-centre randomised controlled trial. BMC Pulm Med. 2020;20(1):143.
35. Ennis S, Sandhu H, Bruce J, et al. Development of a complex exercise rehabilitation intervention for people with pulmonary hypertension: The Supervised Pulmonary Hypertension Exercise Rehabilitation (SPHERe) Trial. BMC Pilot and Feasibility Studies. 2020;Under review.
36. Hennink MM, Kaiser BN, Marconi VC. Code Saturation Versus Meaning Saturation: How Many Interviews Are Enough? Qual Health Res. 2017;27(4):591-608.
37. Chen HY, Boore JR. Translation and back-translation in qualitative nursing research: methodological review. J Clin Nurs. 2010;19(1-2):234-9.
38. Plumridge G, Redwood S, Greenfield S, et al. Involving interpreters in research studies. Journal of Health Services Research & Policy. 2012;17(3):190-2.
39. Regmi K, Naidoo J, Pilkington P. Understanding the Processes of Translation and Transliteration in Qualitative Research. International Journal of Qualitative Methods. 2010;9(1):16-26.

40. Santos HP, Jr., Black AM, Sandelowski M. Timing of translation in cross-language qualitative research. Qual Health Res. 2015;25(1):134-44.
41. Temple B, Young A. Qualitative Research and Translation Dilemmas. Qualitative Research. 2004;4(2):161-78.
42. van Nes F, Abma T, Jonsson H, et al. Language differences in qualitative research: is meaning lost in translation? Eur J Ageing. 2010;7(4):313-6.
43. Moerbeek M, Wong WK. Sample size formulae for trials comparing group and individual treatments in a multilevel model. Stat Med. 2008;27(15):2850-64.
44. NICE. Guide to the methods of technology appraisal. Available at: https://www.nice.org.uk/process/pmg9/chapter/the-reference-case2013.
45. Curtis L, Burns A. Unit Costs of Health and Social Care 2019. Personal Social Services Research Unit, University of Kent, Canterbury2018.
46. Health Measures. Department of Health. Reference Costs 2015-16 In: Health Do, editor. Available at: https://assets.publishing.service.gov.uk/government/uploads/system/uploads/attachment_data/file/577083/Reference_Costs_2015-16.pdf2016.
47. NICE. Position statement on use of the EQ-5D-5L value set for England. Available at: https://www.nice.org.uk/about/what-we-do/our-programmes/nice-guidance/technology-appraisal-guidance/eq-5d-5l2019.
48. Manca A, Hawkins N, Sculpher MJ. Estimating mean QALYs in trial-based cost-effectiveness analysis: the importance of controlling for baseline utility. Health Econ. 2005;14(5):487-96.
49. Sterne JAC, White IR, Carlin JB, et al. Multiple imputation for missing data in epidemiological and clinical research: potential and pitfalls. BMJ. 2009;338:b2393.
50. White IR, Horton NJ, Carpenter J, et al. Strategy for intention to treat analysis in randomised trials with missing outcome data. Bmj. 2011;342:d40.
51. White IR, Royston P, Wood AM. Multiple imputation using chained equations: Issues and guidance for practice. Statistics in Medicine. 2011;30(4):377-99.
52. Gale NK, Heath G, Cameron E, et al. Using the framework method for the analysis of qualitative data in multi-disciplinary health research. BMC Med Res Methodol. 2013;13:117.

12. APPENDICES

12.1 Appendix 1 - Logic model for the REGAIN psychological intervention.

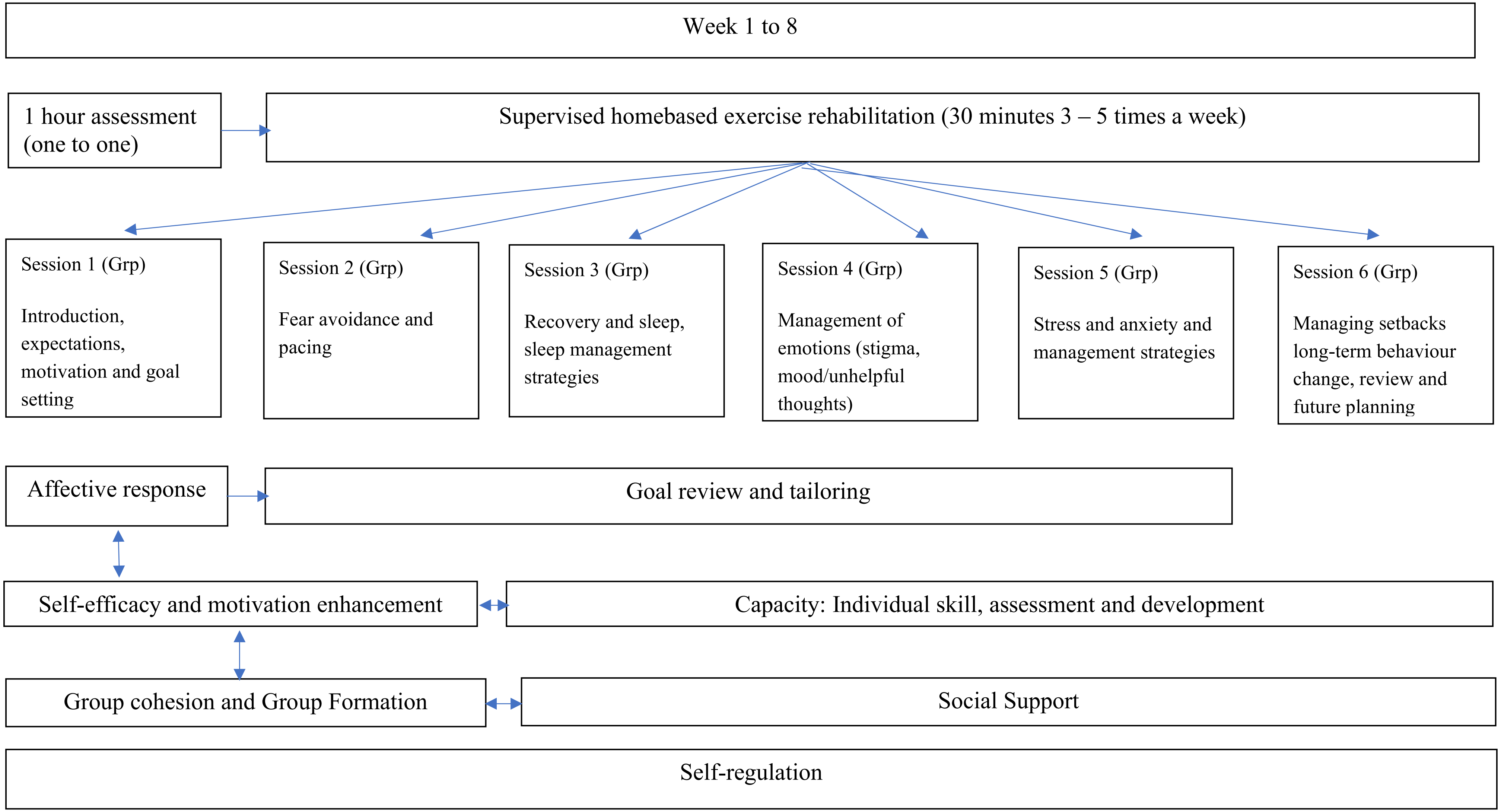

Statistical Analysis Plan

Version 2.0

Date: 25 October2022

Protocol version: 7.0, 18 Jan 2022

IRAS No: 288362

REC and Approval date: Cambridge South REC (08 Dec 2020)

REC ref: GM497120

ISRCTN: 11466448

Version	Date	Edited by	Comments/Justification	Timing in relation to unblinded interim monitoring	Timing in relation to unblinding of Trial Statisticians
0.1	08/01/21	CJ	First draft.	Prior	Prior
0.2	30/07/21	KB	First draft.	Prior	Prior
0.3	29/09/21	KB	First draft.	Prior	Prior
0.4	09/06/22	MR	First draft	Prior	Prior
1.0	10/08/22	MR	First draft	Prior	Prior
2.0	25/10/2022	MR	First draft	Prior	Prior

APPROVAL

Lead Trial Statistician	Name: Prof Ranjit Lall, Warwick Clinical Trials Unit	
	Signature:	Date:07/11/2022
Chief Investigator	Name: Dr Gordon McGregor, UHCW	
	Signature:	Date: 03/11/2022
Senior Statistician Data Monitoring Committee	Name: Prof Dawn Teare, Newcastle University	
	Signature:	Date: 04/11/2022
Senior Statistician Trial Steering Committee	Name: Prof Reuben Ogollah, University of Nottingham	
	Signature:	Date: 03/11/2022

1 Table of Contents

Abbreviations 5
List of authors and reviewers 6
Roles and responsibilities 6
1 INTRODUCTION 7
2 BACKGROUND INFORMATION 7
2.1 Rationale for trial 7
2.2 Objectives of the trial 8
2.2.1 *Primary objective* 8
2.2.2 *Secondary objectives* 8
2.3 Trial design 8
2.4 Eligibility 8
2.4.1 *Inclusion criteria* 8
2.4.2 *Exclusion criteria* 9
2.5 Interventions 9
2.5.1 *Study Interventions* 9
2.6 Definitions of primary and secondary outcomes 10
2.6.1 *Primary outcome* 10
2.6.2 *Secondary clinical outcomes* 10
2.6.3 *Symptoms sub-study* 13
2.7 Hypothesis framework 13
2.8 Sample size 13
2.9 Randomisation 14
2.10 Data collection schedule 14
2.11 Data monitoring and interim analysis 15
2.12 Trial reporting 16
3 ANALYSIS 16
3.1 Subject population 16
3.1.1 Intention-to-treat 16
3.1.2 Missing data 16
4 DESCRIPTIVE ANALYSES 17
4.1 Participant throughput 17
4.2 Baseline comparability of randomised groups 17
4.3 Losses to follow-up 17

4.4 Adherence to treatment 17
5 COMPARATIVE ANALYSES 17
5.1 Primary analysis 18
5.1.1 *Primary outcome* 18
5.2 Secondary analyses 18
5.3 Subgroup analyses 18
5.4 Sensitivity analyses 19
5.4.1 Additional covariate analysis 19
5.4.2 Imputed analysis 19
5.4.3 CACE analysis 19
5.5 Significance levels and adjustments of p-values for multiplicity 19
5.6 Statistical software employed 19
6 SAFETY DATA 20
7 ADDITIONAL EXPLORATORY ANALYSIS 20
8 DEVIATION FROM ANALYSIS DESCRIBED IN PROTOCOL 20
9 REFERENCES 20
9.1 Trial documents 20
9.2 Other references 20

Abbreviations

Abbreviation	Explanation
AE	Adverse Event
CI	Confidence Interval
COM-B	Capability, Opportunity and Motivation
CONSORT	Consolidated Standards of Reporting Trials
COVID-19	Coronavirus disease
CRF	Case report form
DMC	Data Monitoring Committee
GCP	Good Clinical Practice
HADS	Hospital Anxiety and Depression Scale
HRA	Health Research Authority
HRQoL	Health-Related Quality of Life
ICF	Informed Consent Form
IES-6	Impact of Event Scale – 6
IES-R	Impact of Event Scale – Revised
IPAQ-SF	International Physical Activity Questionnaire short form
IQR	Interquartile range
ITT	Intention-to-treat
MET	Metabolic equivalent task
MI	Multiple Imputation
NIH	National Institute for Health
PIS	Patient Information Sheet
PP	Per protocol
PROMIS	Patient-Reported Outcomes Measurement Information System
PTSD	Post-traumatic stress disorder
QoL	Quality of Life
SAE	Serious Adverse Event
SAP	Statistical analysis plan
SOP	Standard Operating Procedure
TSC	Trial Steering Committee
TBC	To be confirmed
USA	United States of America
WCTU	Warwick Clinical Trials Unit

List of authors and reviewers

Authors

Chen Ji, Trial Statistician, Warwick Clinical Trials Unit (WCTU), University of Warwick

Katie Booth, Trial Statistician, Warwick Clinical Trials Unit (WCTU), University of Warwick

Mariam Ratna, Trial Statistician, Warwick Clinical Trials Unit (WCTU), University of Warwick

Reviewers

Professor Ranjit Lall, Lead Trial Statistician, Warwick Clinical Trials Unit (WCTU), University of Warwick

Dr Gordon McGregor, Chief Investigator (CI), Department of Cardio-pulmonary rehabilitation, University Hospitals Coventry & Warwickshire

Prof Dawn Teare, DMC Chair and Statistician, Newcastle University

Prof Reuben Ogollah, University of Nottingham (TSC committee member)

Roles and responsibilities

Trial Statisticians

Professor Ranjit Lall, Dr Chen Ji, Katie Booth, Dr Mariam Ratna

Role: To develop the statistical analysis plan and conduct the final analysis.

Data Monitoring Committee (DMC)

Prof Dawn Teare (Chair), Newcastle University

Prof Nicholas Hart, St Thomas' Hospital

Prof Christopher Armitage, University of Manchester

1 INTRODUCTION

This document details the proposed presentation and analysis for the main results from the multi-centre randomised controlled trial REGAIN (ISRCTN 11466448) which aims to investigate the clinical and cost-effectiveness of an intensive, on-line, supervised, group, home-based rehabilitation programme (REGAIN) vs best practice usual care, to support the long-term physical and mental health recovery of people discharged from hospital (more than three months) after COVID-19 infection.

The results reported in the funder report and main paper(s) will follow the strategy set out here. Any subsequent analysis of a more exploratory nature will not be bound by this strategy and will be detailed in a separate statistical analysis plan (SAP). Suggestions for subsequent analyses by oversight committees, journal editors or referees, will be considered carefully in line with the principles of this analysis plan.

Any deviations from the final, approved SAP will be described and justified in the final report to the funder. The statistical analysis will be carried out by an identified, appropriately qualified and experienced medical statistician, who will ensure the integrity of the data during their processing.

2 BACKGROUND INFORMATION

2.1 Rationale for trial

Early data from COVID-19 survivors shows that a proportion of people experience persistent cognitive impairment, and pulmonary hypertension in those with thromboembolic problems [1]. For the 45% of people hospitalised with COVID-19 in the UK who are estimated to require prolonged support from health and social care [2], a multitude of physical, psychological and social needs have been identified [1]. For hospitalised patients, long-term physical and psychological consequences are also prominent [3]. A further feature is the disproportionate infection rate and progression to severe illness in Black, Asian and minority ethnic groups [4]. We have no data on whether ethnicity affects the prevalence or pattern of long-term sequelae from COVID-19.

To tackle the multiple long-term physical and mental health consequences of COVID-19, it is clear that a complex, multi-disciplinary, physical and psychological rehabilitation intervention should be tested. Importantly, this must be delivered at the appropriate point in the recovery timeline. It must also be cost-effective and deliverable at scale whilst adhering to continued general population infection control measures. Further, it must address ethnic and cultural health inequalities.

Please refer to latest version of the protocol: presently 7.0, 18 January 2022.

2.2 Objectives of the trial

2.2.1 *Primary objective*

The primary objective of this study is to determine whether the online, supervised, group REGAIN rehabilitation intervention improves health-related quality of life (HRQoL) at three months post-randomisation compared to best-practice usual care in patients with ongoing COVID-19 symptoms.

2.2.2 *Secondary objectives*

Secondary objectives of the study are to determine if the REGAIN intervention compared to best-practice usual care in patients with ongoing COVID-19 symptoms impacts on the following outcomes over 12 months. Outcomes listed below are measured at three, six and 12 months:

1. HRQoL (**primary at three months)**
2. Dyspnoea
3. Cognitive function
4. Health utility
5. Physical activity
6. PTSD symptom severity
7. Depressive and anxiety symptoms
8. Work status
9. Health and social care resource use
10. General health
11. All-cause mortality.

2.3 Trial design

REGAIN is a multi-centre, randomised controlled trial (RCT) testing the clinical and cost-effectiveness of the REGAIN intervention vs. best practice usual care. The trial design includes:

1. An intervention development phase, to confirm feasibility, refine online intervention delivery and manualised practitioner training, and prepare study set-up.
2. An internal pilot phase, with formative process evaluation, to test recruitment and study procedures.
3. A main trial with embedded process evaluation.

2.4 Eligibility

2.4.1 *Inclusion criteria*

1) UK resident;
2) Aged ≥18 years;
3) ≥ 3 months after any hospital discharge related to COVID-19 infection, regardless of need for critical care or ventilatory support;
4) Substantial, as defined by the participant, COVID-19 related physical and/or mental health problems;
5) Access to, and ability/support to use, email, text message, internet video, including webcam and audio;
6) Ability to provide informed consent;

7) Able to understand spoken and written English, Bengali, Gujarati, Urdu, Punjabi, or Mandarin themselves or with support from family/friends.

2.4.2 *Exclusion criteria*

1) Exercise contraindicated*
2) Severe mental health problems preventing engagement**
3) Previous randomisation in the present study
4) Patient already engaging in, or planning to engage in a conflicting NHS delivered rehabilitation programme in the next 12 weeks
5) A member of the same household has previously been randomised in the present study

* As advised by a clinical member of the research team or REGAIN practitioner
** As judged by a clinical member of the research team or the REGAIN practitioner

2.5 Interventions

Patients are randomised into one of two groups: the REGAIN Intervention or best practice usual care.

2.5.1 *Study Interventions*

The REGAIN intervention has three components:

1. **Individual assessment:** up to one-hour, on-line, one-to-one assessment with a REGAIN practitioner (Clinical Exercise Physiologist/Physiotherapist), trained and supported by a health psychologist, to holistically assess participant needs, introduce the programme, and provide individualised exercise advice. All participants are directed to freely available on-line programmes published by NHS England (https://www.yourcovidrecovery.nhs.uk/).

   Participants with case level mental health disorders (depression/anxiety/PTSD), as identified from baseline questionnaires (IES-6 score ≥11; HADS Anxiety score ≥11; HADS Depression score ≥11), will be directed to their GP for treatment/advice. These symptomatic participants will continue in the study intervention as long as the practitioner considers their mental health problems would not preclude engagement.

2. **On-line, home-based, exercise rehabilitation:** Up to 30 minutes exercise two to three times per week for eight weeks; individualised and progressive multi-modality exercise at a manageable intensity (regulated with breathlessness and perceived exertion scales).

   Participants are encouraged to attend one live on-line group exercise session every week for eight weeks led by a REGAIN practitioner, using equipment-free exercise to improve cardiovascular fitness, strength, balance, and co-ordination. These sessions are

undertaken in discrete groups. Participants remain in the same group for the 8-week programme. If requested, some groups can be single sex.

For the remaining 1-2 exercise sessions per week, participants are encouraged to access online, pre-recorded video sessions, graded by ability and exercise modality.

3. **Psychological support:** Over the eight-week intervention period, participants attend six on-line group sessions each lasting for up to one hour, led by a trained REGAIN practitioner supported by a health psychologist.

Best practice usual care

A thirty-minute, on-line, one-to-one consultation with a REGAIN practitioner, trained and supported by a health psychologist. All study participants, in both intervention arms, are directed to freely available on-line programmes published by NHS England (https://www.yourcovidrecovery.nhs.uk/).

2.6 Definitions of primary and secondary outcomes

2.6.1 *Primary outcome*

Health-related quality of life measured using the PROMIS® 29+2 Profile v2.1 (PROPr) at three months post-randomisation. This measure is part of a portfolio of outcomes developed and validated by the National Institute for Health (NIH) (USA); the Patient-Reported Outcomes Measurement Information System. It is a reliable generic outcome measure validated for on-line use [6-8] generating a single overall score (from -0.2 to 1) plus physical function, anxiety, depression, fatigue, sleep disturbance, social roles/activities, pain interference, cognitive function and pain intensity sub-scales. A higher score indicates better quality of life.

Justification for timing of primary outcome

Long-term outcomes are important, however, any intervention effects will be maximal soon after completion of the intervention. We have set our short-term follow-up at three months as we are confident that those randomised to the REGAIN intervention will complete the eight-week treatment phase in this time period. If there is no evidence of effect at three months, then a meaningful effect at one year is unlikely. Assessing the primary outcome at three months after randomisation is more efficient than seeking an effect at one year, as attrition is likely to be lower.

2.6.2 *Secondary clinical outcomes*

The following outcomes will be measured at 3, 6 and 12 months post-randomisation.

1. HRQoL: PROMIS® 29+2 Profile v2.1 (PROPr) at 6 and 12 months post randomisation. This form includes all 29 items from PROMIS® 29 Profile v2.1, plus two Cognitive Function abilities items. The 29 items from PROMIS® 29 Profile v2.1 are from the following forms;

Items	Number of items
PROMIS SF v2.0 Physical Function	4
PROMIS SF v1.0 Anxiety	4
PROMIS SF v1.0 Depression	4
PROMIS SF v1.0 Fatigue	4
PROMIS SF v1.0 Sleep Disturbance	4
PROMIS SF v1.0 Ability to Participate in Social Roles and Activities	4
PROMIS SF v1.0 Pain Interference	4
Cognitive Function SF v2.0	2
PROMIS SF Pain Intensity	1

A single overall preference score ranging from -0.2 to 1 (perfect or ideal health) is generated using sub-scores from the multiple forms. Zero indicates as state equal to death and a negative value indicates worse than death. This outcome will be scored anonymously using the HealthMeasures Scoring Service (https://www.assessmentcenter.net/ac_scoringservice), as recommended by the PROMIS Adult Profile Scoring Manual.

2. Dyspnoea: PROMIS dyspnoea severity Short Form. Exertional dyspnoea is a commonly reported symptom in COVID-19 survivors, thus specific questions have been added [9]. The dyspnoea short form includes 10 questions, with each response ranging from 0 to 3, giving a total raw score ranging from 0 to 30 (higher scores indicate worse severity). This measure will be scored using the HealthMeasures Scoring Service (https://www.assessmentcenter.net/ac_scoringservice).

3. Cognitive function: Neuro-QoL Short Form v2.0 - Cognitive Function [13]. Given the high incidence of cognitive impairment in COVID-19 survivors we have added additional PROMIS questions, to obtain a measure of cognitive function. The Cognitive function short form has 8 items, with total raw scores ranging from 8 to 40 (higher scores indicate better cognitive function). This measure will be scored using the HealthMeasures Scoring Service (https://www.assessmentcenter.net/ac_scoringservice).

4. Health utility: Euroqol EQ-5D-5L [10]. Validated, generic HRQoL measure consisting of five dimensions, each with five levels. Each combination of answers can be converted into a health utility score. It has good test-retest reliability, is simple to use, and gives a single preference-based index value for health status that can be used for cost-effectiveness analysis. A statement by NICE highlighted serious concerns regarding the EQ-5D-5L tariffs published by Devlin et al [11]. For that reason, the crosswalk value set will be used to map from the EQ-5D-5L to EQ-5D-3L using previously used and more reliable tariff values. The EQ-5D score ranges from <0-1 where a higher score reflects better quality of life.

5. International Physical Activity Questionnaire (IPAQ short-form). The IPAQ is a well-established activity measure reported as metabolic equivalent task (MET)-minutes per week derived from duration of walking, moderate and vigorous exercise [12] The questionnaire will be scored using the IPAQ Scoring protocol (https://sites.google.com/site/theipaq/scoring-protocol).

6. PTSD symptom severity: The Impacts of Events Scale-Revised (IES-R) a 22 item self-report measure of difficulties people sometimes face after stressful life events. It has been widely used in studies of survivors of ICU admission, including COVID admissions. It is part of recommended outcomes for studies of respiratory failure survivors [13-15]. The IES-R is scored by summing the response to each of the 22 questions, which each range from 0 (not at all) to 4 (extremely), making a total score range of 0-88. A score of ≥11 on the IES-6, an abbreviated version extracted from the longer 22-item IES-R, will be taken to be indicative of case level disorder.

7. Depressive and anxiety symptoms: Hospital Anxiety and Depression Scale (HADS). A 14-item questionnaire from which anxiety and depression subscales can be derived. 7 item sub-score values ≥11 points identify case-level anxiety/depression. The HADs is widely used and a well validated measure in clinical populations [16]. The scores are simply summated to give an anxiety and depression score both ranging from 0-21 where a higher score reflects more severe symptoms of anxiety and depression.

8. Death measured using GP record. Data will be requested from general practices on completion of the trial.

Timing and format of data collection

Patient reported outcomes are collected online at baseline pre-randomisation, and at three months, six months and 12 months post-randomisation. Participants receive an email notification and/or text message to remind them to complete the online questionnaires at each follow-up time point. In the case of non-response to text messages, participants are contacted by telephone for collection of two core outcomes: the PROPr (primary outcome) and EQ-5D-5L.

Fluency in English is not an inclusion criterion for this study. For those not fluent in English, we will aim to collect all outcomes (or as many as possible) verbally at each follow-up. As a minimum, a core data outcome set including the PROMIS® 29+2 Profile v2.1 (PROPr) and EQ-5D-5L questionnaires will be collected orally by a bilingual researcher, where necessary, to ensure that those not fluent in English are able to contribute participant reported outcomes to the study. The EQ-5D-5L is well validated for verbal administration.

Long-term follow-up beyond 12 months: Consent will be sought from participants to hold their personal data, and at the end of the 12-month follow-up period, to request a copy of the participant’s medical record from their GP. This will only be requested if the participant has not responded to the 12-month follow-up or if we know the participant has died. This will provide information on GP consultations and include copies of any hospital discharge letters allowing us to accurately cost in-patient care costs. Where appropriate, we will triangulate data from GP records and any participant self-report to achieve a robust estimate of health service activity and mortality.

2.6.3 *Symptoms sub-study*

Study sites identifying patients will record information on patient hospital admission data including length of hospital stay and ventilation type. This will be pseudonymised using a screening ID number assigned to each patient by the study site. Any patients approached by a study site will provide their screening ID number and using this screening ID number, for those patients consenting to the study, WCTU will request pseudonymised data for that individual from the study site that approached the participant. Ongoing COVID-19 symptoms will be collected during the initial online eligibility assessment, supplemented by the external clinical expertise from the TSC and DMC. This will allow us to compare selected factors including patient characteristics and COVID-19 admission characteristics, and ongoing COVID-19 symptoms profile of those who take part in the study. Where possible, this sub-study will be undertaken.

2.7 Hypothesis framework

For each of the primary and secondary outcomes, the null hypothesis will be that there is no true difference in treatment effect between the intervention arms.

2.8 Sample size

We had no data on which to base a sample size estimation for the study. There are no normative data for the PROPr quality of life scores in this Covid-19 population and no external indication of what might be a worthwhile benefit from the intervention on quality of life outcomes for this population. American values for the general population in the USA are a mean score of 50 (1-100 scale) with an SD of 10. Whilst not our preferred practice, we have used the approach of looking for a small to moderate standardised mean effect size of 0.3. Allowing for a clustering effect in the intervention arm, we assume that a group size will consist of a maximum of eight participants. Then assuming an intra cluster coefficient (ICC) of 0.01, 90% power and type I error rate of 5%, with a 10% loss to follow-up, we require 535 participants. This equates to 272 participants in the intervention arm across up to 34 intervention groups and 263 participants in the control arm (control:intervention = 1:1.03), using computations recommended by Moerbeek [17].

The sample size was revised as requested by the data monitoring committee (DMC) in the DMC meeting (14 January 2022). However, following this meeting because of the change in recruitment strategy and thus the large recruitment from the NHS Digital mass mailouts there was no need to update the sample size. The recruitment of the trial was completed in few months following the meeting and finally 585 participants were recruited. We overrecruited to compensate the higher lost to follow-up (15%) in the observed data.

2.9 Randomisation

Randomisation is undertaken automatically by the WCTU system following completion of the baseline questionnaire using a computer-generated randomisation sequence, performed by minimisation and stratified by:

1. age (i. <65; ii. ≥65 years),
2. level of hospital care (i. ICU/HDU; ii. ward),
3. case level mental health disorder (i. IES-6 PTSD score ≥11/24 or HADS Anxiety sub-score ≥11/21 or HADS Depression sub-score ≥11/21; ii. IES-6 PTSD score <11/24 and HADS Anxiety sub- score <11/21 and HADS Depression sub- score <11/21).

Participants are randomised strictly sequentially at study level.

2.10 Data collection schedule

All data are entered directly by participants, UHCW staff, REGAIN practitioners or WCTU study team members onto a secure online study database hosted by WCTU as outlined in the data management plan and in accordance with the Warwick SOPs. Data entered onto the online study database are considered source data. This will be stored safely and securely. On all study-specific documents, other than the completed consent form, the participant will be referred to by the study participant number, not by name. Various methods will be used to chase missing data including phone, text and email. The procedures for managing this will be outlined in the data management plan and appropriate consent will be sought to contact participants. Data will still be collected for participants who discontinue or deviate from the intervention protocol, unless they withdraw their consent.

Table 1: Study assessments and data collected at Trial time points

	**Pre-randomisation**		**Post-randomisation**			
**Online assessment**	**1**	**2**	**3**	**4**	**5**	**6**
**Assessment time point**	**Screening**	**Enrolment (Baseline)**	**Intervention Delivery 0- 8 weeks (+/- 2 weeks)**	**3m (± 2w)**	**6 m (± 1 m)**	**12 m (± 1 m)**
Invitation letter and flyer posted	✓					
Initial Eligibility Assessed	✓					
Concomitant Illnesses		✓				
Eligibility check* (telephone)		✓				
Informed consent		✓				

	**Pre-randomisation**		**Post-randomisation**			
**Online assessment**	**1**	**2**	**3**	**4**	**5**	**6**
**Assessment time point**	**Screening**	**Enrolment (Baseline)**	**Intervention Delivery 0- 8 weeks (+/- 2 weeks)**	**3m (± 2w)**	**6 m (± 1 m)**	**12 m (± 1 m)**
Patient Demographics		✓				
PROMIS® 29+2 Profile v2.1 (PROPr)		✓		✓	✓	✓
PROMIS dyspnoea		✓		✓	✓	✓
PROMIS Neuro-QoL		✓		✓	✓	✓
EQ-5D-5L		✓		✓	✓	✓
IPAQ-SF		✓		✓	✓	✓
IES-R		✓		✓	✓	✓
HADS		✓		✓	✓	✓
Intervention			✓			
Adverse events			✓			
Overall health		✓		✓	✓	✓
Death						✓

* Eligibility check will be performed in person over the telephone by clinical member of the research team at UHCW. All other assessments and information will be completed by the participant online.

For long term follow-up assessments, consent will be sought from participants to keep their personal data. Consent will also be taken to request a copy of the participant's medical record from their GP, should they not respond to the 12-month follow-up questionnaire, or have died at the end of the study follow-up period. This will provide information on GP consultations and include copies of any hospital discharge letters allowing us to accurately cost in-patient care costs. Mortality data will be gathered from GP records at 12 months.

2.11 Data monitoring and interim analysis

All study data will be supplied in strict confidence to the independent DMC for independent assessment and evaluation. The DMC will consist of independent experts with relevant clinical research, and statistical experience. The DMC meeting frequency will be guided by the DMC chair but will be suggested to be six months into the recruitment phase and regularly thereafter, as directed by the DMC chair. Confidential reports containing recruitment, protocol compliance, safety data and informal interim assessments of outcomes will be

reviewed by the DMC. The DMC will advise the TSC as to whether there is evidence or reason why the trial should be amended or terminated. The membership of the DMC has been approved and appointed by the NIHR.

There are no formal interim analyses for this study.

2.12 Trial reporting

The trial will be reported in line with the CONSORT (Consolidated Standards *o*f *R*eporting *T*rials) statement[18].

3 ANALYSIS

3.1 Subject population

3.1.1 Intention-to-treat

The primary analysis and any secondary analyses will be applied to an all-randomised population on an intention-to-treat (ITT) basis. That is, any subject randomised into the trial, regardless of whether they received trial intervention and regardless of protocol deviations, unless specified.

3.1.2 Missing data

Whilst every effort will be made to ensure complete data collection, it is inevitable that some data may not be available due to voluntary withdrawal of participants, lack of completion of individual data items or general loss to follow-up. Where possible the reasons for data 'missingness' will be ascertained and reported. The nature and pattern of the missingness will be carefully considered, including whether data can be treated as missing completely at random. If judged appropriate, missing data will be imputed using the multiple imputation facilities available in statistical analysis software.

If imputation is undertaken, the resulting imputed datasets will be analysed, together with appropriate sensitivity analyses. Any imputation methods used for scores and other derived variable will be carefully considered and justified. Reasons for ineligibility, non-compliance, withdrawal, or other protocol violations will be stated, and any patterns summarised. If the missing data is considered missing at random or missing completely at random, we will use imputation techniques, such as Multiple Imputation by Chained Equations (MICE), to impute missing data. The number of imputed datasets will be determined by the proportion of missing data. Usually 10-20 datasets would be sufficient for 10-30% missing data [19].

More formal analysis, for example using logistic regression with 'protocol violation' as a response, may also be appropriate and aid interpretation.

4 DESCRIPTIVE ANALYSES

4.1 Participant throughput

Screening data will be checked to identify any characteristic differences between those individuals in the trial, those ineligibles, and those eligible but withholding consent. A CONSORT chart illustrating participant flow throughout the trial will also be produced which will describe the following: number of participants randomised, allocated to each intervention, delivered and not delivered intervention, lost to follow-up, and included in ITT analysis population at different time points.

4.2 Baseline comparability of randomised groups

Baseline data will be summarised to check comparability between treatment arms. The number and percentage will be presented for categorical variables. The mean and standard deviation or the median and the interquartile range (IQR) will be presented for continuous variables, or the range if appropriate. There will be no tests of statistical significance performed nor confidence intervals calculated for differences between randomised groups on any baseline variable.

4.3 Losses to follow-up

The number and percentage of participants lost to follow-up before 12 months post-randomisation will be reported for each randomised group.

4.4 Adherence to treatment

The number and proportion of patients who did and did not receive the intervention they were allocated to will be reported. Full compliance with the online, exercise and psychological REGAIN intervention will be defined as attending the initial assessment, plus attending four out of six psychological support sessions, AND five out of eight live exercise sessions.

5 COMPARATIVE ANALYSES

All analyses will be conducted as ITT unless otherwise specified.

The screening data collects; Date of admission, Date of discharge and ventilation support (highest level of ventilation support during admission: Invasive ventilation [highest level]/Non-invasive ventilation/CPAP [lowest level]). A CONSORT chart illustrating participant flow throughout the trial will also be produced. Standard statistical summaries will be presented for the primary outcome measure and all secondary outcome measures.

For continuous outcomes, mean treatment difference with 95% confidence interval (CI) will be reported. For categorical outcomes, odds ratio with 95% CI will be reported. Plots will also be produced for the primary outcome and some of the secondary outcomes to check the comparability between treatment arms.

5.1 Primary analysis

5.1.1 *Primary outcome*

The main analyses will be for overall treatment effect. The primary analysis will use a linear regression (heteroscedastic) model to estimate the treatment effects (95% confidence intervals (CI)), adjusted for baseline as well as adjusted for stratification variables, important patient-level covariates and group effect. The reason for using heteroscedastic model is the variance is different between the arms as the control arm is non-clustered and the intervention arm is clustered [20]. In the trial, the control arm receives individual therapy whereas the intervention arm receives group therapies. The groups or therapist effect will be treated here as random effects. If there is negligible group and centre effect, then the usual linear regression will be used for the analysis.
The main analysis will be carried out on the stratification variables such as:

- Age (continuous)
- Level of hospital care (i. ICU/HDU; ii. ward)
- Case level mental health disorder (i. IES-6 PTSD score ≥11/24 or HADS Anxiety sub-score ≥11/21 or HADS Depression sub-score ≥11/21; ii. IES-6 PTSD score <11/24 and HADS Anxiety sub- score <11/21 and HADS Depression sub- score <11/21)

5.2 Secondary analyses

For the primary outcome, we will also use a linear regression (heteroscedastic) to estimate the treatment effect, without adjustment of any covariates but adjusting the therapist random effects.

All secondary continuous outcomes (including the health economic outcomes, EQ-5D-5L index and VAS scores) will be analysed using the linear regression (heteroscedastic) specified in the primary analysis method, with the same adjustment. An unadjusted analysis will also be conducted for each outcome.

We will assess the impact of compliance on outcomes using a CACE (Compliers average causal effect) analysis for the primary outcome. CACE models evaluate the average effect of the intervention in participants who comply with their allocated treatment. This preserves randomisation groups and eliminates introducing any potential confounders introduced by PP analysis.

For categorical outcomes, odds ratio with 95% CI will be reported. We will also plot the data over time, with the mean and confidence intervals, for some of the secondary outcomes such as the International Physical Activity Questionnaire (IPAQ short-form).

5.3 Subgroup analyses

Exploratory subgroup analyses will examine the interaction of treatment assignment with the pre-specified subgroups, including:

1. Age group (<65 vs >=65)
2. Level of hospital care (Critical care vs Ward)

3. Depression (HADS depression score <11 vs >=11)
4. Anxiety (HADS anxiety score <11 vs >=11)
5. PTSD (IES-r <11 vs >=11)
6. Ethnicity (BAME vs non-BAME)
7. Wave of pandemic (1st Wave: March 2020, 2nd Wave: September 2020, 3rd Wave: TBC)
8. Method of recruitment (NHS digital mailouts vs others)

These subgroup analyses will be conducted in the observed dataset on the basis of ITT population. The analyses will use hierarchical linear regression model with adjustment for covariates in the primary analysis. The overall significance of the interaction will be reported. Estimated treatment difference in each subgroup will be reported with mean treatment difference with 95% CI.

5.4 Sensitivity analyses

5.4.1 Additional covariate analysis

The sensitivity analysis on the primary outcome will be carried out on the additional variables such as:

- Sex (Male, Female, Other, Prefer not to say)
- BMI (Continuous)
- Ethnicity (White, Black Caribbean, Black African, Black Other, Indian, Pakistani, Bangladeshi, Chinese, Mixed- White and Black Caribbean, Mixed- White and Black African, Mixed- White and Asian, Prefer not to say, Other)*

* Sex and *Ethnicity categories may be grouped together if numbers are too low*

5.4.2 Imputed analysis

The primary analysis will be replicated to analyse the imputed datasets. if imputation is deemed appropriate. The pooled results will be reported.

5.4.3 CACE analysis

Also, it is likely that non-compliance will occur (i.e. exercise sessions not attended or participant requests for treatment) during the trial. Careful monitoring of non-compliance will be conducted. If large numbers of treatment non-compliance are observed, Complier-Average Causal Effect (CACE) models or other appropriate methods will be used. The CACE analysis will also be replicated using different definitions of compliance.

5.5 Significance levels and adjustments of p-values for multiplicity

Treatment effects will be presented, with appropriate 95% confidence intervals, for both the unadjusted and adjusted analyses. Tests will be two-sided and considered to provide evidence for a significant difference if p-values are less than 0.05 (5% significance level). All analyses will be conducted as ITT unless otherwise specified. No adjustment of p-values in the analysis as there is no multiple comparison in the analyses.

5.6 Statistical software employed

All analyses will be conducted using Stata/SE version 17 (or later), SAS version 9 (or later), or R version 4 (or later).

6 SAFETY DATA

The frequency and percentage (%) of serious adverse events (SAE) and adverse events (AE) in the trial will be reported by treatment with the following details: the event type, severity assessment, expectedness and relatedness to intervention will also be summarised/analysed by treatment arm.

7 ADDITIONAL EXPLORATORY ANALYSIS

Any post-hoc analysis requested by the oversight committees, a journal editor or referees will be labelled explicitly as such. Any further future analyses not specified in the analysis protocol will be exploratory in nature and will be documented in a separate statistical analysis plan.

8 DEVIATION FROM ANALYSIS DESCRIBED IN PROTOCOL

None yet.

9 REFERENCES

9.1 Trial documents

Dummy tables can be found in the separate document.

9.2 Other references

1. National Health Service. *After-care needs of inpatients recovering from COVID-19*. 2020; Available from: https://www.cambscommunityservices.nhs.uk/docs/default-source/luton-adults-general/c0388_after_care_needs_of_inpatients_recovering_from_covid-19_5_june_2020.pdf.
2. National Health Service. *COVID-19 Hospital Discharge Service Requirements.* . NHS England, HM Government: Crown Copyright 2020; Available from: https://www.england.nhs.uk/coronavirus/publication/covid-19-hospital-discharge-service-requirements/.
3. Holmes, E.A., et al., *Multidisciplinary research priorities for the COVID-19 pandemic: a call for action for mental health science.* Lancet Psychiatry, 2020. **7**(6): p. 547-560.
4. Public Health England. *Beyond the data: Understanding the impact of COVID-19 on BAME groups*. 2020; Available from: https://www.gov.uk/government/publications/covid-19-understanding-the-impact-on-bame-communities.
6. Hays, R.D., et al., *PROMIS(®)-29 v2.0 profile physical and mental health summary scores.* Qual Life Res, 2018. **27**(7): p. 1885-1891.
7. Rose, A.J., et al., *Evaluating the PROMIS-29 v2.0 for use among older adults with multiple chronic conditions.* Qual Life Res, 2018. **27**(11): p. 2935-2944.
8. Cella, D., et al., *PROMIS(®) Adult Health Profiles: Efficient Short-Form Measures of Seven Health Domains.* Value Health, 2019. **22**(5): p. 537-544.
9. Cella, D., et al., *PROMIS((R)) Adult Health Profiles: Efficient Short-Form Measures of Seven Health Domains.* Value Health, 2019. **22**(5): p. 537-544.
10. Herdman, M., et al., *Development and preliminary testing of the new five-level version of EQ-5D (EQ-5D-5L).* Qual Life Res, 2011. **20**(10): p. 1727-36.
11. Devlin, N., et al., *Valuing Health-Related Quality of Life: An EQ-5D-5L Value Set for England. OHE Research Paper 16/01. London: Office of Health Economics*, 2016. 27(1): p.22-7
12. Craig, C.L., et al., *International physical activity questionnaire: 12-country reliability and validity.* Med Sci Sports Exerc, 2003. **35**(8): p. 1381-95.

13. Hosey, M.M., et al., *The IES-R remains a core outcome measure for PTSD in critical illness survivorship research.* Crit Care, 2019. **23**(1): p. 362.
14. Needham, D.M., et al., *Core Outcome Measures for Clinical Research in Acute Respiratory Failure Survivors. An International Modified Delphi Consensus Study.* Am J Respir Crit Care Med, 2017. **196**(9): p. 1122-1130.
15. Weiss, D.S. and C.R. Marmar, *The Impact of Event Scale—Revised*, in *Assessing psychological trauma and PTSD.* 1997, The Guilford Press: New York, NY, US. p. 399-411.
16. Somaini, G., et al., *Prevalence of Anxiety and Depression in Pulmonary Hypertension and Changes during Therapy.* Respiration, 2016. **91**(5): p. 359-66.
17. Moerbeek, M. and W.K. Wong, *Sample size formulae for trials comparing group and individual treatments in a multilevel model.* Stat Med, 2008. **27**(15): p. 2850-64.
18. Moher, D., K.F. Schulz, and D.G. Altman, *The CONSORT statement: revised recommendations for improving the quality of reports of parallel-group randomised trials.* Lancet, 2001. **357**(9263): p. 1191-4.
19. Graham, J.W., A.E. Olchowski, and T.D. Gilreath, *How many imputations are really needed? Some practical clarifications of multiple imputation theory.* Prev Sci, 2007. **8**(3): p. 206-13.
20. Roberts C, Roberts SA. Design and analysis of clinical trials with clustering effects due to treatment. Clinical trials. 2005 Apr;2(2):152-62.
